# PARP-1 improves leukemia outcomes by inducing parthanatos during chemotherapy

**DOI:** 10.1016/j.xcrm.2023.101191

**Published:** 2023-09-07

**Authors:** Bruktawit Maru, Alessandra Messikommer, Linhui Huang, Katja Seipel, Olivia Kovecses, Peter J.M. Valk, Alexandre P.A. Theocharides, Francois E. Mercier, Thomas Pabst, Maureen McKeague, Nathan W. Luedtke

**Affiliations:** 1 Department of Pharmacology and Therapeutics, McGill University, Montreal, QC, Canada; 2 Department of Chemistry, University of Zurich, Zurich, Switzerland; 3 Department of Medical Oncology, Inselspital, Bern University Hospital, Bern, Switzerland; 4 Department of Hematology, Erasmus University Medical Center, Rotterdam, the Netherlands; 5 Department of Medical Oncology and Hematology, University of Zurich and University Hospital Zurich, Zurich, Switzerland; 6 Division of Hematology and Experimental Medicine, Department of Medicine, McGill University, Montreal, QC, Canada; 7 Department of Chemistry, McGill University, Montreal, QC, Canada

**Keywords:** cancer biology, nucleoside analog, caspase-independent programmed cell death, acute myelomonocytic and monocytic leukemia, NAD+ ADP-ribosyltransferase 1, PARP-1, poly(ADP-ribose), PAR, apoptosis, prognostic blood test, precision medicine

## Abstract

Previous chemotherapy research has focused almost exclusively on apoptosis. Here, a standard frontline drug combination of cytarabine and idarubicin induces distinct features of caspase-independent, poly(ADP-ribose) polymerase 1 (PARP-1)-mediated programmed cell death “parthanatos” in acute myeloid leukemia (AML) cell lines (n = 3/10 tested), peripheral blood mononuclear cells from healthy human donors (n = 10/10 tested), and primary cell samples from patients with AML (n = 18/39 tested, French-American-British subtypes M4 and M5). A 3-fold improvement in survival rates is observed in the parthanatos-positive versus -negative patient groups (hazard ratio [HR] = 0.28–0.37, p = 0.002–0.046). Manipulation of PARP-1 activity in parthanatos-competent cells reveals higher drug sensitivity in cells that have basal PARP-1 levels as compared with those subjected to PARP-1 overexpression or suppression. The same trends are observed in RNA expression databases and support the conclusion that PARP-1 can have optimal levels for favorable chemotherapeutic responses.

## Highlights

• Parthanatos is a clinically relevant cell death pathway during frontline chemotherapy
• Drug treatment can induce parthanatos or apoptosis in primary white blood cells
• Parthanatos features provide an independent favorable risk factor for certain leukemias
• Optimal levels of PARP-1 can support favorable AML chemotherapeutic responses

## Graphical abstract

**Figure:**
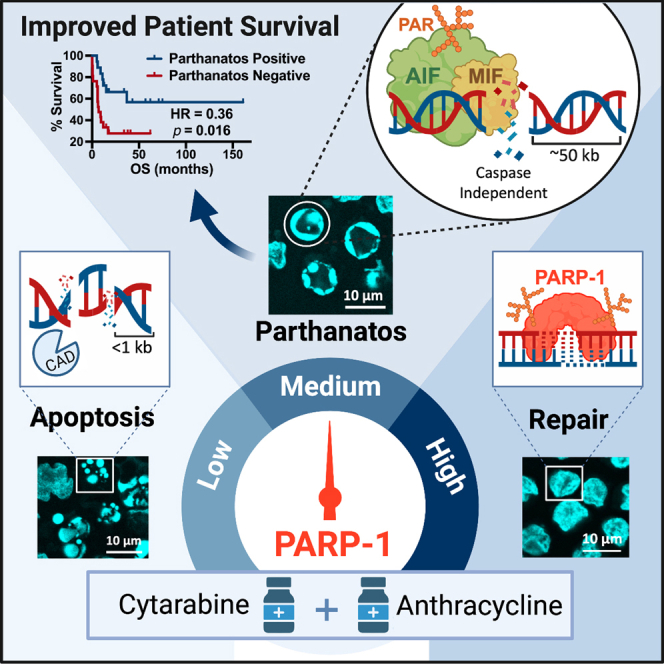

## Teaser

Maru et al. report that PARP-1-mediated programmed cell death, parthanatos, is associated with the successful frontline treatment of certain acute myeloid leukemias (AMLs). PARP-1 expression and cell death characteristics of drug-treated primary AML cells are prognostic, and they may motivate the future development of targeted therapies that inhibit parthanatos suppressors.

## Introduction

Acute myeloid leukemia (AML) is the most common and malicious form of leukemia in adults, with an approximate 30% 5-year overall survival rate for patients receiving curative therapies.^1^ The histology-based French-American-British (FAB) subclassification system helped identify acute promyelocytic leukemia (APL; subtype M3) as a separate and highly curable subtype of AML, but it only accounts for ∼10% of patients with AML.^2^ The remaining 90% of patients with AML are classified by a prognosis-based system established by the World Health Organization and European LeukemiaNet (ELN) that divides patients into three genetic risk groups (favorable, intermediate, and adverse) based on the presence of cytogenetic and mutational biomarkers. Despite growing numbers of new palliative and auxiliary AML therapies becoming available,^3^ the frontline, curative treatment of eligible patients from all three risk groups commences with a 7-day infusion of cytarabine (ara-C) that is typically augmented with an anthracycline for 3 days known as “7+3” induction chemotherapy.^4^ Even after 50 years of widespread clinical use, the mechanisms responsible for ara-C’s selective killing of AML cells remain poorly understood. Previous studies have focused on metabolic incorporation of ara-C into DNA and downstream apoptosis.^5^^,^^6^^,^^7^^,^^8^ However, recent reports suggest that ara-C incorporation into primer RNA is responsible for its therapeutic efficacy,^9^ and active killing of AML primary isolates by ara-C can be a caspase-independent process.^10^

DNA damage and stimulation of apoptosis have been focal areas of chemotherapeutic drug development for leukemia and other cancers for nearly 50 years.^11^ Cells that lose tumor-suppressor activities by p53 mutation^12^ can exhibit dysregulated apoptosis and resistance against standard chemotherapeutic drugs.^13^^,^^14^ Targeted therapies that sensitize cells toward apoptosis such as B cell leukemia/lymphoma-2 (Bcl-2) family inhibitors^15^ as well as p53 “reactivators”^16^ can overcome drug resistance. However, primary AML cells treated with ara-C can undergo caspase-independent programmed cell death,^10^ and caspase-3 activation trends failed to predict ara-C and anthracycline drug sensitivity of primary AML isolates from 42 patients.^17^ A potential explanation for these observations is that healthy and diseased white blood cells may undergo non-apoptotic forms of programmed cell death during chemotherapy such as autophagic cell death,^18^^,^^19^ necroptosis,^20^^,^^21^ pyroptosis,^22^ or parthanatos.^23^ As part of our ongoing mechanistic studies of ara-C,^9^^,^^24^ we discovered parthanatos features in the common AML cell line OCI-AML3 and therefore designed a study to evaluate the clinical relevance of parthanatos in 7+3 induction chemotherapy of AML.

Parthanatos has been recognized as a distinct mode of programmed cell death by the Nomenclature Committee on Cell Death since 2012.^25^^,^^26^ Poly(ADP-ribose) polymerase 1 (PARP-1) is the primary orchestrator of parthanatos,^27^ and its abnormal expression is associated with poor clinical outcomes in breast cancer^28^ and AML.^29^^,^^30^^,^^31^ PARP-1-mediated cell death was previously reported in white blood cells treated with reactive oxygen species.^23^^,^^32^ Parthanatos proceeds via activation of PARP-1 by chromatin stress,^33^ accumulation of poly(ADP-ribose) (PAR), and translocation of apoptosis-inducing factor (AIF) from the mitochondria into the nucleus, where it activates the nuclease “macrophage migration inhibitory factor” (MIF).^34^^,^^35^^,^^36^ Once activated, MIF selectively cleaves single-stranded DNA to generate large chromatin fragments of ∼50 kb.^34^ Parthanatos can proceed with retention of plasma membrane integrity and externalization to phosphatidylserine onto the outer surface of the cell. As such, parthanatos cannot be distinguished from apoptosis by standard assay kits with only annexin V and propidium iodide staining. Additional experiments are required to distinguish between parthanatos and apoptosis, such as chromatin fragment analysis,^37^^,^^38^ evaluating the impact of caspase inhibition,^39^ and measuring the impact of PARP-1 inhibition on drug sensitivity.^40^^,^^41^

Here, we report that parthanatos is associated with successful frontline treatment of a common cancer. The presence/absence of two key parthanatos features, PARP-dependent changes in drug sensitivity and characteristic nuclear “ring” morphologies, are highly correlated (χ^2^_(3)_ = 29.1, p ≤ 0.0001) in samples taken from 39 patients with AML of FAB subtypes M4+M5. The presence of both features (n = 18 of 39) was associated with 3-fold improvements in overall survival (hazard ratio [HR] = 0.356, p = 0.016) and event-free survival rates (HR = 0.365, p = 0.019). Multivariate analyses taking gender, age at diagnosis, ELN risk group, and cancer cell load as covariates suggest that the presence of parthanatos features is an independent, favorable risk factor for the overall survival of patients with M4/M5 AML treated with 7+3 chemotherapy (HR = 0.367, p = 0.046). Manipulation of PARP-1 expression in parthanatos-competent cells revealed that basal PARP-1 expression supports higher drug sensitivity as compared to cells subjected to PARP-1 overexpression, inhibition, or silencing. Analyses of mRNA expression databases revealed that near-median expression of *PARP1* by patients with FLT3 wild-type, M4/M5 AML receiving 7+3 chemotherapy is associated with a 2-fold lower death rate (HR = 0.56, p = 0.011). Together, these results demonstrate that PARP-1 can have optimal activity levels for chemotherapeutic responses in AML.

## Results

### Ara-C induces parthanatos in OCI-AML3 and apoptosis in OCI-AML2 cells

**Figure 1:**
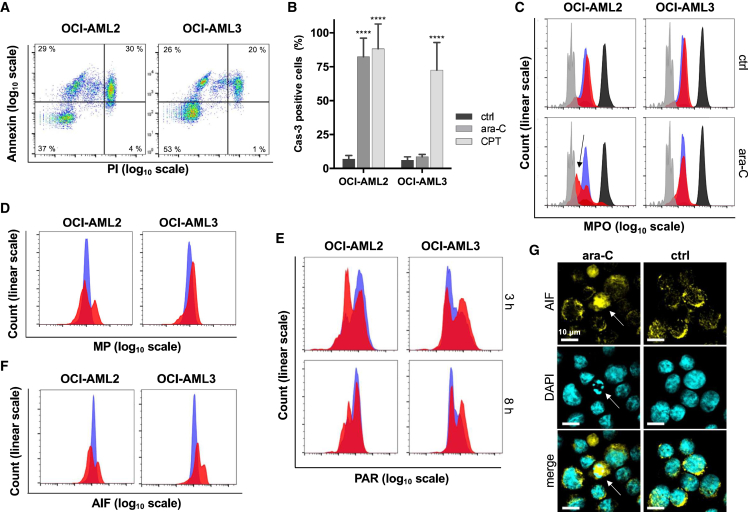
Cytarabine induces apoptosis in OCI-AML2 cells and parthanatos in OCI-AML3 cells (A) Phosphatidylserine exposure on the cell surface was detected by Annexin V staining, and membrane integrity was probed using propidium iodide (PI) following a 24-h treatment of OCI-AML2 cells (1 μM) or OCI-AML3 cells (10 μM) with ara-C for 24 h. Untreated (control) cells were >95% double negative for PI and Annexin V staining. (B) According to the cleavage of a fluorogenic substrate in live cells, ara-C treatment stimulated caspase-3 activity in OCI-AML2 cells (5 μM) but not OCI-AML3 cells (15 μM) after 8 h. Camptothecin (CPT) was used as positive control.^46^ Averaged values ± SD for n = 3 biological replicates are shown. (C) Fluorescence quenching revealed mitochondrial pore opening (MPO) in OCI-AML2 (black arrow), but not in OCI-AML3, cells according to the MitoProbe Transition Pore Assay and flow cytometry of OCI-AML2 cells treated with 1 μM ara-C and OCI-AML3 cells treated with 10 μM of ara-C for 24 h. Blue: untreated; red: ara-C treated; black: staining control calcein AM; gray: positive control ionomycin. (D) Mitochondrial membrane potentials (MPs) following ara-C treatment of OCI-AML2 cells (1 μM) and OCI-AML3 cells (10 μM) for 24 h. Blue: untreated; red: ara-C treated. (E) After 3 h of ara-C treatment, PAR accumulated in OCI-AML3 cells (10 μM), but was diminished in OCI-AML2 cells (1 μM), according to immunofluorescent staining. Blue: untreated; red: ara-C treated. (F) After 4 h of ara-C treatment, higher levels of AIF staining were observed in treated OCI-AML3 cells (10 μM) as compared with OCI-AML2 cells (1 μM). Blue: untreated; red: ara-C treated. (G) Immunostaining of AIF and microscopy revealed the release of AIF in OCI-AML3 cells treated with 10 μM ara-C for 4 h. The arrow highlights a cell with a characteristic “ring” structure of DNA and high abundance of released AIF. ∗∗∗∗p < 0.0001. Control (ctrl) samples were not treated with ara-C; n = 3 biological replicates (A–G).

Steady-state plasma concentrations of ara-C typically range from 0.3^42^ to 115 μM^43^ during initial “induction” and subsequent “consolidation” chemotherapy of AML. Here, ara-C was applied at concentrations ranging from 0.5 to 30 μM, which are near the EC_50_ values for OCI-AML2 and OCI-AML3 cell lines.^24^ These cell lines were derived from FAB subtype M4 patients (representing ∼25% of AML) and express wild-type *PARP1*^44^ and *TP53*.^45^ Ara-C treatment of OCI-AML2 and OCI-AML3 cells for 24 h caused phosphatidylserine exposure to the surfaces of both cell lines without loss of membrane integrity (Figure 1A). After 8-h incubations, caspase-3 activation was present in OCI-AML2 but absent in OCI-AML3 (Figure 1B). As a positive control, the topoisomerase inhibitor camptothecin (CPT)^46^ activated caspase-3 in both cell lines (Figure 1B). Consistent with the features of classical apoptosis, an opening of mitochondrial pores (black arrow, Figure 1C) and loss of mitochondrial membrane potentials (Figure 1D) were observed in OCI-AML2 cells upon treatment with ara-C. In contrast, OCI-AML3 cells did not form mitochondrial transition pores, and hyperpolarization of the mitochondrial membranes was observed after a 24-h treatment. These changes, together with the concurrent maintenance of cell membrane integrity, demonstrate that ara-C induces neither classical apoptosis nor necrosis in OCI-AML3 cells. To evaluate the possibility that OCI-AML3 cells undergo parthanatos upon ara-C treatment, we quantified changes in PAR accumulation^47^ and AIF translocation.^48^ OCI-AML3 and not OCI-AML2 cells rapidly accumulated PAR polymers after a 3-h treatment with ara-C (Figure 1E) and released AIF after 4 h (Figures 1F and 1G).

### Different modes of programmed cell death are reflected in distinctive nuclear morphologies

**Figure 2:**
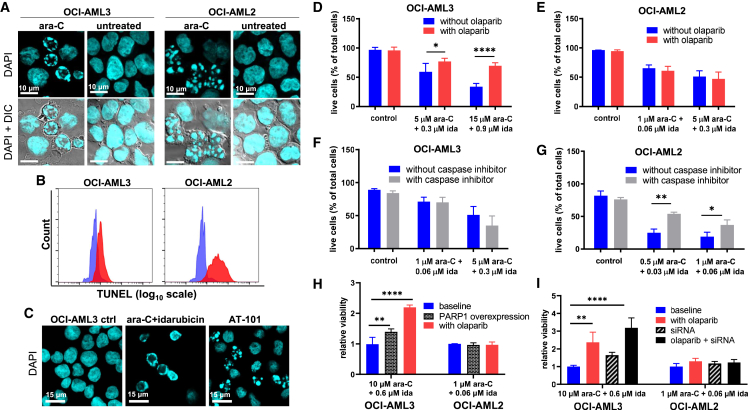
Additional parthanatos features in OCI-AML3 cells (A) DAPI staining revealed globular chromatin fragments of highly variable sizes throughout the nuclei of OCI-AML2 cells treated with 1 μM ara-C for 24 h, whereas a relatively small number of large chromatin fragments are distributed in a ring-shaped pattern at the nuclear periphery of OCI-AML3 cells treated with 10 μM of ara-C for 24 h. ara-C concentrations were selected based on EC_50_ values for toxicity in each cell line. (B) The TUNEL assay confirmed a relatively broad distribution of small DNA fragments in OCI-AML2 as compared with OCI-AML3 cells following treatment with 1 and 10 μM ara-C, respectively, for 24 h. Blue: untreated; red: ara-C treated. (C) Nuclear morphologies in OCI-AML3 cells following 24-h treatment with a 17:1 mixture of ara-C and idarubicin as compared with 30 μM of the BH3 mimetic AT-101 (Gossypol) that selectively stimulates apoptosis. (D) Live/dead staining and flow cytometry analysis of OCI-AML3 cells treated with 1 μM of the PARP inhibitor olaparib for 24 h prior to treatment with a 17:1 mixture of ara-C and idarubicin (ida) for 24 h. (E) Live/dead staining and flow cytometry analysis of OCI-AML2 cells treated with 1 μM of the PARP inhibitor olaparib for 24 h prior to treatment with ara-C and ida for 24 h. (F) Trypan blue exclusion test of cell viability for OCI-AML3 cells treated with 10 μM of the caspase inhibitor Z-VAD-FMK 24 h prior to treatment with ara-C and ida for 24 h. (G) Trypan blue test of cell viability for OCI-AML2 cells treated with 10 μM of the caspase inhibitor Z-VAD-FMK 24 h prior to treatment with ara-C and ida for 24 h. Ctrl samples were not treated with ara-C or ida. ∗p < 0.05, ∗∗p < 0.01, and ∗∗∗∗p < 0.0001; DIC, differential interference contrast image; n = 3 biological replicates (A–C). Data are represented as mean ± SEM for n = 3 technical replicates of 3 biological replicates (D–G). (H) Relative viability (number of live cells in each experimental sample/control sample) of OCI-AML3 and OCI-AML2 cells receiving pre-treatment with a PARP-1-overexpressing plasmid for 72 h and/or 1 μM olaparib for 24 h prior to addition of ara-C and ida for 24 h. (I) Relative viability of OCI-AML3 and OCI-AML2 cells receiving pre-treatment with siRNA for 72 h and/or 1 μM olaparib for 24 h prior to addition of ara-C and ida for 24 h. Error bars: SEM; statistical tests for (H) and (I) were conducted using two-way ANOVA with Tukey’s multiple comparison test. ∗p < 0.03, ∗∗p < 0.002, ∗∗∗p < 0.0002, and ∗∗∗∗p < 0.0001. n = 3 technical replicates of 2 biological replicates (H and I).

DAPI staining and microscopy revealed the formation of globular chromatin fragments in OCI-AML2 cells treated with ara-C for 24 h (Figure 2A). In contrast, chromatin fragments in a distinctive “ring-shape” pattern were observed at the nuclear periphery of treated OCI-AML3 cells (Figure 2A). We speculated that this unique chromatin morphology is associated with the cleavage of chromatin into a relatively small number of large fragments in late parthanatos.^37^^,^^38^ To evaluate this possibility, the TUNEL assay was used to compare the relative number of DNA cleavage sites in OCI-AML2 and OCI-AML3 cells treated with ara-C (Figure 2B). Indeed, a much larger number of DNA strand breaks was detected in treated OCI-AML2 versus OCI-AML3 cells, consistent with a higher frequency of DNA cleavage events in late apoptosis versus parthanatos. When treated with a clinically relevant, 17:1 ratio mixture of ara-C and the anthracycline drug idarubicin,^49^ the OCI-AML3 cells again exhibited ring-shaped chromatin patterns (Figure 2C). In contrast, globular nuclear morphologies, consistent with the formation of apoptotic bodies, were observed in OCI-AML3 cells treated with the BH3 mimetic AT-101 (Gossypol), which activates apoptosis.^50^^,^^51^ These results suggested that OCI-AML3 cells can undergo either parthanatos or apoptosis—depending on the specific drug(s) being added—and that characteristic changes in nuclear morphologies reflect the type of programmed cell death mechanism in operation.

### PARP inhibition, but not caspase inhibition, protects OCI-AML3 cells from ara-C and idarubicin-induced cell death

To further evaluate the presence of parthanatos, we applied 1 μM of the PARP inhibitor olaparib^52^ for 24 h, followed by escalating doses of ara-C (1–15 μM) and idarubicin (0.06–0.9 μM) in a fixed ratio of 17:1.^49^ This olaparib concentration (1 μM) had no measurable impact on the viability of OCI-AML2 or OCI-AML3 cells, and it is approximately 10-fold below the peak plasma concentration reached in humans receiving oral olaparib (Lynparza).^53^ However, olaparib pre-treatment caused an 18%–36% increase in the number of living OCI-AML3 cells treated with ara-C/idarubicin as compared with those receiving no olaparib pre-treatment (Figure 2D). In contrast, olaparib caused a small decrease in the number of viable OCI-AML2 cells when treated with the same mixture (Figure 2E). To evaluate the presence/absence of caspase-mediated cell death, cells were pre-treated with the irreversible pan-caspase inhibitor Z-VAD-FMK for 24 h, followed by ara-C and idarubicin for 24 h, and then we counted them for the percentage of viable cells.^39^ Caspase inhibition had no measurable impact on the sensitivity of OCI-AML3 cells toward ara-C/idarubicin treatment (Figure 2F), whereas Z-VAD-FMK caused OCI-AML2 cells to become more resistant toward ara-C/idarubicin (Figure 2G). These results confirm that OCI-AML3 cells undergo caspase-independent parthanatos, whereas OCI-AML2 cells undergo apoptosis upon addition of ara-C and idarubicin.

### Both PARP-1 overexpression and interference lower the drug sensitivity of OCI-AML3 cells

To evaluate the relationships between PARP-1 expression and drug sensitivity, we evaluated changes in cell-killing activities of ara-C/idarubicin mixtures following PARP-1 overexpression by plasmid, PARP inhibition by olaparib, or PARP-1 silencing by small interfering RNA (siRNA). Transfection of a PARP-1-expressing plasmid into OCI-AML2 and OCI-AML3 cell lines achieved a ∼2-fold increase in *PARP1* expression according to qRT-PCR analysis of isolated RNA and PARP-1 protein according to western blot (Figures S1A–S1C). Following PARP-1 overexpression, a reduction in drug sensitivity toward ara-C/idarubicin was observed in OCI-AML3 cells but not in OCI-AML2 cells (Figure 2H). To evaluate the impact of PARP-1 suppression on drug sensitivity, siRNA was transfected and/or the PARP inhibitor olaparib was added to the cells 24 h prior to ara-C/idarubicin. siRNA transfection caused a transient, 2-fold reduction in *PARP1* RNA expression and PARP-1 protein quantities according to western blot (Figures S2A–S2C). PARP-1 silencing by siRNA and/or the addition of olaparib caused a reduction in drug sensitivity of OCI-AML3 cells but not OCI-AML2 cells (Figure 2I). Together, these results suggested that PARP-1 can have optimal levels for favorable chemotherapeutic responses in parthanatos-competent cells.

### Multiple AML cell lines exhibit parthanatos features upon treatment with ara-C and idarubicin

**Figure 3:**
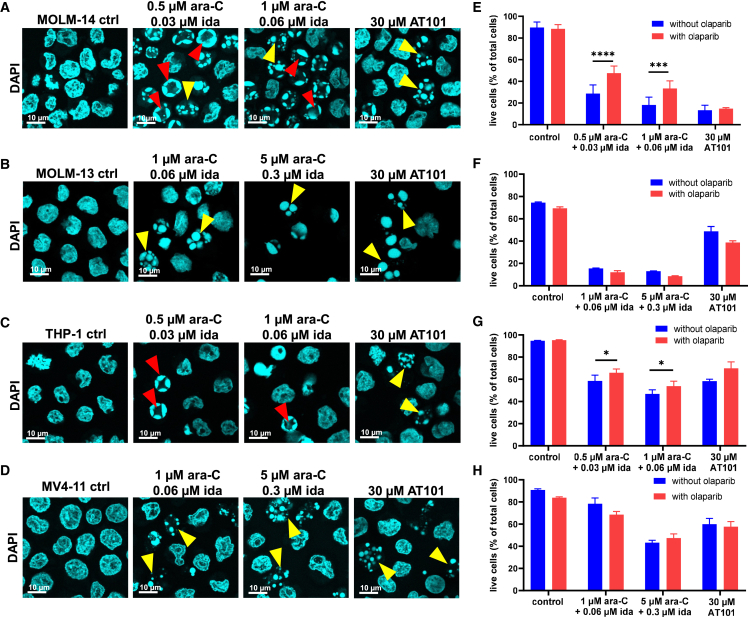
Analyses of four common AML cell lines for parthanatos features following treatment with ara-C and ida (A–D) Nuclear fragmentation pattern analysis was conducted using DAPI staining, cytospin centrifugation, and confocal microscopy of cells 8 h following addition of ara-C and ida: (A) MOLM-14 (FAB M5); (B) MOLM-13 (FAB M5); (C) THP-1 (FAB M5); and (D) MV4-11 (FAB M5). Red arrowheads indicate ring-shaped nuclear fragmentation. Yellow arrowheads indicate globular nuclear fragmentation. (E–H) Percentage of live cells quantified by flow cytometry after 1 μM olaparib pre-treatment for 24 h and a 17:1 mixture of ara-C/ida treatment for 24 h: (E) MOLM-14 (FAB M5); (F) MOLM-13 (FAB M5); (G) THP-1 (FAB M5); and (H) MV4-11 (FAB M5). Statistical analysis was conducted using two-way ANOVA with Sidak multiple comparisons test; data are represented as mean ± SD for n = 3 replicates. ∗p < 0.03, ∗∗p < 0.002, ∗∗∗p < 0.0002, and ∗∗∗∗p < 0.0001.

Commonly used AML cell lines were evaluated for two characteristic parthanatos features: the formation of chromatin ring structures (Figures 3A–3D) and the impact of PARP inhibition by olaparib on cell viability (Figures 3E–3H) upon treatment with ara-C and idarubicin. Among the eight additional AML cell lines evaluated, MOLM-14 and THP-1 (FAB subtypes M5) exhibited both characteristic parthanatos features (Figures 3A, 3C, 3E, and 3G). The remaining six cell lines MOLM-13 (M5), MV4-11 (M5), ME-1 (M4), ML-2 (M4), PL-21 (M3), and HL-60 (M2) exhibited neither feature (Figures 3 and S3). An examination of mutational profiles revealed no obvious differences between the parthanatos-positive versus -negative groups according to the Cancer Dependency Map.^54^ This is highlighted by MOLM-13 (parthanatos-negative) and MOLM-14 (parthanatos-positive) cell lines that were derived from the same patient and share similar mutations including MLL-AF9 fusion and FLT3-ITD.^55^ In contrast, MOLM-13 cells exhibit antigen staining (CD13^−^, CD14^−^, CD64^−^, CD87^−^) consistent with a lesser degree of hematopoietic maturation as compared with MOLM-14 cells (CD13^+^, CD14^+^, CD64^+^, CD87^+^).^55^ OCI-AML2, MV4-11, and MOLM13 (parthanatos-negative) cell lines exhibited more CD34 staining (marker for immaturity) as compared with OCI-AML3 cells (parthanatos positive).^56^ Here, two parthanatos-positive (OCI-AML3 and THP-1) and two parthanatos-negative (OCI-AML2 and MV4-11) cell lines were stained for the myeloid differentiation markers CD11b and CD14. Only the parthanatos-positive cells were stained by both CD11b and CD14 immunofluorescence (Figure S4A). Upon addition of ara-C and idarubicin, CD11b expression was increased in OCI-AML2, but not OCI-AML3, cells, and olaparib pre-treatment had no measurable impact on staining either cell line (Figure S4B). Together, these results suggest that AML cells that have a higher degree of hematopoietic maturation are more likely to exhibit both parthanatos features.

### PBMCs from healthy human donors exhibit parthanatos features upon treatment with ara-C and idarubicin *ex vivo*

**Figure 4:**
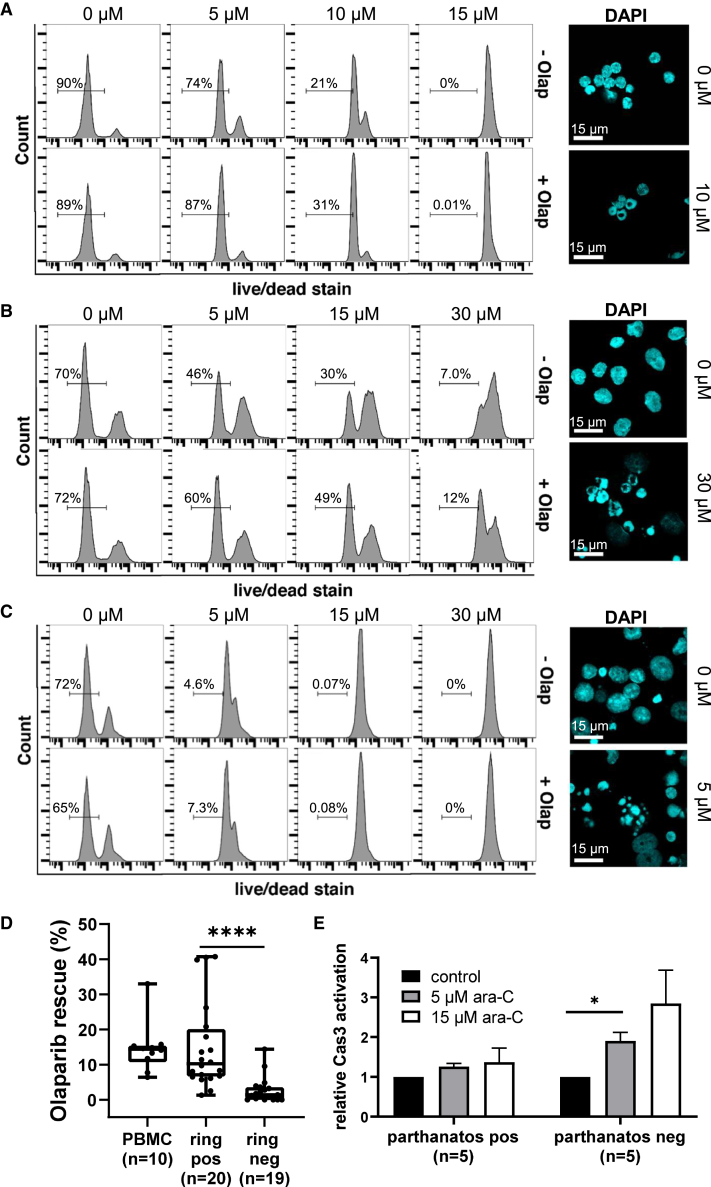
Analysis of primary AML blasts and healthy PBMCs for two parthanatos features upon treatment with ara-C and ida *ex vivo* (A) PBMCs from healthy human donor (#7) treated with 1 μM of the PARP inhibitor olaparib (Olap) for 24 h prior to treatment with ara-C and ida for 24 h. Toxicity rescue was determined using a differential staining cytotoxicity assay,^57^ and nuclear fragmentation patterns were analyzed using DAPI staining, cytospin centrifugation, and confocal microscopy. Semi-quantitative image analyses of data from all 10 donor samples (Data S1) indicate that the ring-shaped nuclear patterns were observed in 14%–46% of the treated PBMCs as compared with only 0.3%–2.7% of the untreated PBMCs. Cells treated with either ara-C or ida alone also exhibited the characteristic DNA “ring” morphologies (Data S2). (B) Primary cells from a patient with AML (ID 17-008) exhibiting both parthanatos features following treatment with ara-C and ida for 24 h (see Data S4 for all 18 examples). Concentrations are given in terms of ara-C. (C) Primary cells from a patient with AML (ID 04-015) exhibiting neither parthanatos feature following treatment with ara-C and ida for 24 h. See Data S5 for all 21 examples of primary AML isolates exhibiting zero or one parthanatos feature. (D) Summary of percentage of cells rescued from ara-C/ida toxicity by Olap pre-treatment of PBMCs from healthy donors (n = 10), of primary AML cell samples exhibiting nuclear ring structures (ring positive [pos], n = 20), or of primary AML cells lacking ring structures (ring negative [neg], n = 19). (E) Caspase-3 activation in primary AML blast samples taken from parthanatos-pos and parthanatos-neg patient groups following 8-h incubations with ara-C and ida according to the cleavage of a fluorogenic caspase-3 substrate in live cells. Error bars: SD; statistical analysis was done by one-way ANOVA with Tukey’s multiple comparison test. ∗p < 0.03.

Peripheral blood mononuclear cells (PBMCs) from healthy donors (n = 10) were treated with the PARP inhibitor olaparib (1 μM, 24 h) prior to ara-C and idarubicin for 24 h. The number of live cells was counted using differential staining^57^ and flow cytometry. Olaparib pre-treatment caused a 6.4%–33% increase in the number of live PBMCs, and all 10 samples exhibited ring-shaped nuclear morphologies (Figure 4A; Data S1; Table S1). To evaluate the ability of the PBMCs to exhibit variable types of programmed cell death characteristics, additional samples from five donors were treated with ara-C, idarubicin, or AT-101 for 24 h and subjected to DAPI staining and microscopy. Nuclear morphologies consistent with parthanatos were observed in all five samples treated with an ara-C/idarubicin mixture or with ara-C or idarubicin alone. In contrast, globular chromatin morphologies consistent with apoptosis were observed in all five PBMC samples treated with AT-101 (Data S2).

### Parthanatos morphologies in the blood cells of patients with AML while undergoing chemotherapy with ara-C and idarubicin *in vivo*

A preliminary evaluation of cell death characteristics during frontline AML chemotherapy was conducted by microscopic analysis of PBMCs collected from eight patients with AML (M4 and M5) at 6–24 h after the onset of standard 7+3-day continuous intravenous (i.v.) infusion of ara-C at 200 mg/m^2^ and idarubicin at 12 mg/m^2^.^49^ DAPI staining revealed characteristic parthanatos chromatin ring patterns in samples from all eight patients with AML tested (Data S3).

### The presence of parthanatos features and an absence of caspase activation are observed in ∼50% of primary M4/M5 AML

All studies involving primary AML samples were conducted in a single-blind fashion, where all bioanalyses and assignments were made before the lead authors received any information about the diagnostic profile or clinical outcome of each patient (Table S2). To facilitate comparisons with the results obtained from the M4/M5 parthanatos-positive and -negative AML cell lines, M4 and M5 FAB subgroup patients were the main focus of this study. Primary isolates from 39 M4/M5 patients were collected from blood (n = 33) or bone marrow (n = 6) at the time of diagnosis. The samples were purified using a Ficoll gradient and frozen in media containing 10% DMSO until use. To help mimic *in vivo* growth conditions, the thawed cells were co-cultured with a monolayer of human HS-5 stromal cells for 24 h.^58^ The co-cultured cells were treated with olaparib (1 μM, 24 h) or carrier only (DMSO). On the second day after thawing, the cells were treated with a 17:1 mixture of ara-C (1–30 μM) and idarubicin (0.06–1.8 μM) for an additional 24 h. The samples were counted for viability using differential staining^57^ and flow cytometry. The cancerous AML “blast” cells were analytically distinguished from other cells present using side light scattering and CD45 immunostaining.^58^ In parallel, the same samples were analyzed for parthanatos-associated nuclear morphologies by DAPI staining, cytospin centrifugation, and fluorescence microscopy. Approximately half of the AML patient samples (n = 18) exhibited both toxicity rescue by olaparib as well as ring-shaped nuclear morphologies characteristic of parthanatos (Figure 4B; Data S4; Table S2). The other half exhibited neither feature (n = 16) or only one feature (n = 5) (Figure 4C; Data S5; Table S2). These results demonstrate a highly significant correlation (χ^2^_(3)_ = 29.1, p ≤ 0.0001) between the presence/absence of toxicity rescue by olaparib and the presence/absence of the DNA ring morphologies in these samples (Figure 4D). Nearly all parthanatos-double-negative AML samples (n = 14 of 16 total; Data S5) exhibited morphologies consistent with PBMCs undergoing apoptosis following AT-101 treatment (Figure 2C; Data S2). To evaluate the presence/absence of caspase activation in primary AML cells following treatment with ara-C/idarubicin, we analyzed 10 primary AML blast samples (M4/M5) from parthanatos-positive (n = 5) and parthanatos-negative (n = 5) patient subgroups for changes in caspase activation following 8-h incubations with ara-C and idarubicin. The parthanatos-positive blast samples exhibited little to no caspase activation, whereas the parthanatos-negative blast samples displayed significant caspase activation (Figure 4E). These results suggest that parthanatos versus apoptosis are two distinct and competing forms of programmed cell death in primary AML treated with ara-C and idarubicin.

### The presence of parthanatos features in M4/M5 AML is associated with improved survival

**Figure 5:**
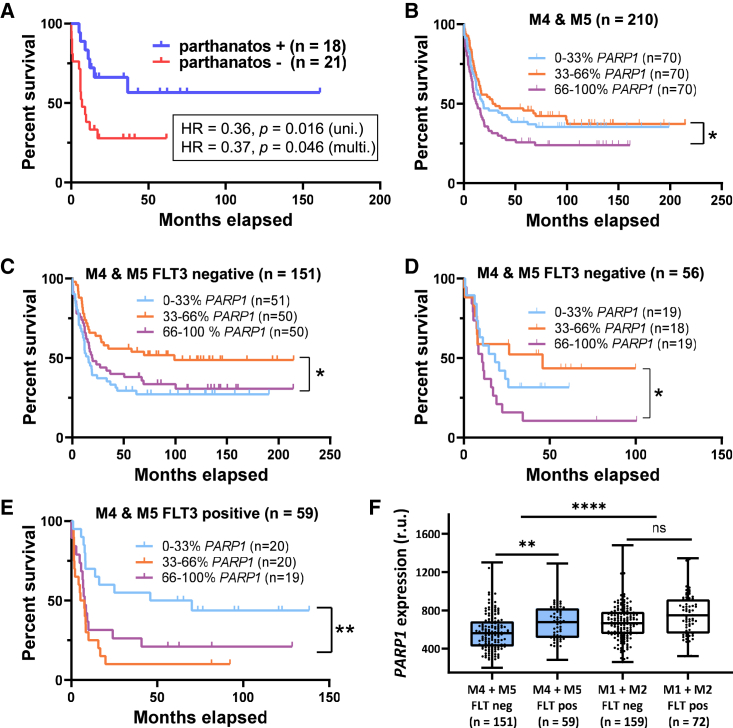
Kaplan-Meier survival estimates of parthanatos-pos (+) versus -neg (−) subgroups, and *PARP1* mRNA expression versus overall survival (A) Overall percentage of survival (OS) versus time of parthanatos+/− subgroups according to assignment 1 (Table 1). See Figure S5 for survival analyses of assignments #2 and #3 and Figure S6 for event-free survival analyses. (B) OS analysis of 210 patients with M4/M5 *de novo* AML (≤60 years old) following 7+3 induction chemotherapy with ara-C and ida versus *PARP1* mRNA quantities measured pre-treatment according to the GEO: GSE6891 microarray dataset. (C) OS analysis of M4/M5 AML FLT3 wild-type (i.e., FLT−) subgroup (n = 151) versus *PARP1* mRNA quantities according to the microarray dataset. (D) OS analysis of M4/M5 AML FLT3 wild-type subgroup (n = 56) versus *PARP1* mRNA quantities according to microarray RNA-seq (TCGA) dataset. (E) OS curves for M4/M5 FLT3-mutated AML subgroup versus *PARP1* mRNA quantities prior to treatment according to the microarray dataset. Patients were grouped into three equal groups based on the relative mRNA expression (low: 0%–33%; middle: 33%–66%; and high: 66%–100%), and OSs were plotted using Kaplan-Meier survival estimates. (F) Boxplot with whiskers (25%–75% interquartile range) illustrating higher relative *PARP1* mRNA levels in patients with FLT3 mutation (FLT3+) versus wild-type (FLT3−) according to microarray dataset. See Figure S6 for OS analyses of M1/M2 AML versus *PARP1*, *PARP2*, *PARG*, and *ARH3*. ∗p < 0.05, ∗∗p < 0.01, ∗∗∗p < 0.001, and ∗∗∗∗p < 0.0001.

Approximately half of all AML patient samples (n = 18 of 39 tested) exhibited both toxicity rescue by olaparib as well as ring-shaped nuclear morphologies characteristic of parthanatos. The remaining samples exhibited neither feature (n = 16) or only one feature (n = 5). Given the ambiguous assignment of patients exhibiting only one feature (n = 5), three separate survival analyses were conducted, where the five patients were considered either parthanatos negative or positive or were excluded from the analysis. In all three univariate analyses, the parthanatos-positive subgroup exhibited a 3-fold higher overall survival rate (Table 1; Figures 5A and S5; HR = 0.28–0.36, p = 0.002–0.016) and event-free survival (Figure S6; HR = 0.27–0.37; p = 0.002–0.019) as compared with the parthanatos-negative group. The inclusion/exclusion of the four patients who were too weak to receive intensive chemotherapy did not impact these conclusions (Figures S5 and S6). Notably, all four of these older patients (69–79 years old) were parthanatos negative according to both assays (Table S2). To test if parthanatos features following exposure to ara-C/idarubicin *ex vivo* are an independent variable for patient outcome, we conducted a multivariate analysis with four well-established risk factors as co-variates: age, gender, genetic risk group, and blast load in the bone marrow (Table 2). The full cohort of this study (n = 39) exhibited the same well-known trends in risk factors associated with increasing age, male gender, ELN adverse risk group, and increasing blast load in the bone marrow (Table 2),^4^^,^^59^ and it was large enough to reach statistical significance for both age (HR = 1.05 per year, p = 0.028) and parthanatos features (HR = 0.367, p = 0.046). The results are consistent with the hypothesis that parthanatos is an independent variable of patient outcome for AML subtypes M4 and M5 treated with ara-C and idarubicin.

**Table 1:** Samples from 39 patients with AML (FAB subtypes M4 and M5) were tested for the presence of chromatin fragmentation “ring” morphologies and PARP-dependent changes in drug sensitivity upon addition of olaparib

Sample type	Total	Exhibited both parthanatos features	Exhibited one feature	Exhibited neither feature
Blood	33	15	4	14
Bone marrow	6	3	1	2
Total	39	18	5	16

**Assignment 1**		**Positive**	**Negative**	

Assigned patients (n)		18	21	
Average age (years)		53 ± 16	59 ± 13	
Gender (% female)		61	29	
ELN genetic risk group	adverse (%)	33	33	
	intermediate (%)	28	24	
	favorable (%)	39	43	
2-year overall survival (%)		66	28	
Log-rank and Mantel-Cox tests: hazard ratio (HR) and p value		0.356, 0.0156		

**Assignment 2**		**Positive**		**Negative**

Assigned patients (n)		23		16
Average age (years)		51 ± 15		63 ± 9
Gender (% female)		61		25
ELN genetic risk group	adverse (%)	35		31
	intermediate (%)	26		25
	favorable (%)	39		44
2-year overall survival (%)		67		17
Log-rank (Mantel-Cox) test: HR and p value		0.291, 0.002		

**Assignment 3**		Positive		Negative

Assigned patients (n)		18		16
Average age (years)		53 ± 16		63 ± 9
Gender (% female)		61		25
ELN genetic risk group	adverse (%)	33		31
	intermediate (%)	28		25
	favorable (%)	39		44
2-year overall survival (%)		66		17
Log-rank (Mantel-Cox) test: HR and p value		0.275, 0.002		

Five of the 39 samples exhibited only one feature in the absence of the other. Assignment of these five patients as being parthanatos positive (assignment 1) or parthanatos negative (assignment 2) or as being excluded from the analysis (assignment 3) had little to no impact on the conclusions drawn in this study.

**Table 2:** Multivariate survival analysis of 39 patients with AML (FAB subtypes M4 and M5) tested for the presence of chromatin fragmentation “ring” morphologies and PARP1-dependent changes in drug sensitivity

Overall survival by feature	Multivariate analysis			Univariate analysis		
	HR	95% CI	p	HR	95% CI	p
Parthanatos positive	0.367	0.138–0.981	0.046	0.356	0.153–0.827	0.016
Age (per year)	1.054	1.006–1.105	0.028	1.030	0.995–1.067	0.092
Gender (female)	0.471	0.172–1.292	0.144	0.616	0.267–1.423	0.248
ELN adverse risk group	2.561	0.960–6.836	0.060	1.503	0.612–3.694	0.334
Blast cells (per %)	1.006	0.981–1.031	0.640	0.998	0.976–1.020	0.838

Samples that exhibited both features were assigned as parthanatos positive (assignment 1). Kaplan-Meier survival estimates were compared by univariate analysis by a Mantel-Cox test and log-rank hazard ratios. The multivariate analysis was conducted using a Cox proportional hazard model and four well-established risk factors as co-variates: age, gender, genetic risk group, and blast load in the bone marrow. CI, confidence interval.

### Preliminary evaluation of FAB subtypes M1/M2 and CD34^+^ hematopoietic stem cells (HSPCs) suggests that more differentiated AMLs undergo parthanatos

Primary samples from eight patients with AML with FAB subtypes M1 or M2 were analyzed for chromatin “ring” fragmentation morphologies and PARP-dependent changes in drug sensitivity as before. An impact of PARP inhibition on drug sensitivity was observed in 6 of 8 of the M1/M2 AML samples, but the characteristic chromatin ring morphologies were observed in only one of eight samples (Table S3). This is in contrast to the near-perfect correlation of these two features observed in primary isolates from patients with M4/M5 AML (Table S2). The absence of ring structure morphologies is likely related to the lower extent of hematopoietic differentiation of M1/M2 subtypes (mostly CD34^+^) as compared with M4/M5 subtypes (∼50% CD34^−^). To evaluate this possibility, we tested normal CD34^+^ HSPCs for parthanatos features according to the presence of ring structures and olaparib rescue. As observed for the M1/M2 AML samples, the HSPCs exhibited a pro-survival impact of PARP inhibition but no ring structure morphologies upon addition of ara-C/idarubicin (Figures S4C and S4D). These results suggest that primary white blood cells having a greater degree of differentiation (M4/M5 AML, PBMCs) exhibit both parthanatos features upon addition of ara-C and idarubicin, whereas less differentiated cells (M1/M2 AML, HSPCs) do not.

### *PARP1* expression is a potential biomarker for intensive chemotherapy of M4 and M5 AML, depending on FLT3 status

Given the low frequency of *PARP1* mutations in hematopoietic and lymphoid blood cancers (41 of 5,212 currently tested^44^) together with the ability of both PARP-1 interference and PARP-1 overexpression to decrease drug sensitivity (Figures 2H and 2I), we hypothesized that median levels of *PARP1* expression can be associated with improved M4/M5 AML survival. To explore this possibility, we analyzed a large mRNA expression dataset (n = 441) from clinical trials organized by the Haemato Oncology Foundation for Adults in the Netherlands (HOVON).^60^ mRNA expression data from patients with AML with FAB subtypes M1 and M2 (n = 231) and M4 and M5 (n = 210) receiving curative treatment including 7+3 chemotherapy with ara-C and idarubicin were stratified into three equal groups based on their relative expression levels of *PARP1* mRNA (low: 0%–33%; middle: 33%–66%; and high: 66%–100%). Patients with M4/M5 AML with the lowest expression of *PARP1* (0%–66%) exhibited a 50% improved overall survival (OS) rate (HR = 0.66, p = 0.016) as compared with the highest expressing (66%–100%) group (Figure 5B). This result is consistent with previous analyses of smaller AML datasets split into high/low expressing groups.^29^^,^^30^^,^^61^ Since FLT3 mutation in AML is known to be associated with *PARP1* overexpression,^29^ here, we separated the FLT3-positive (n = 59) and FLT3 AML-negative (n = 151) subgroups and repeated the analysis. Remarkably, the near-median expressers (middle 33%–66% group) of *PARP1* in FLT3-negative (wild-type) M4/M5 AML exhibited an 85% improved OS rate (HR = 0.56, p = 0.011) as compared with both the highest and lowest expressing groups (Figure 5C). The same trend was also apparent in an RNA sequencing (RNA-seq) database (Figure 5D). In contrast, the FLT3-positive group exhibited a different trend, with the lowest 33% expression group having increased survival as compared with the middle and highest expressing groups (Figure 5E; HR = 0.38, p = 0.004). These differences likely reflect FLT3 mutation-driven overexpression of PARP-1.^29^ Consistent with the different phenotypes of M1/M2 versus M4/M5 AML blasts *ex vivo*, FLT3 status had no apparent impact on *PARP1* expression by M1/M2 AML (Figure 5F), and no correlation between *PARP1* expression (or other known parthanatos-mediating genes) and survivability was observed for M1/M2 AML (Figure S7).

## Discussion

For nearly 50 years, chemotherapy-induced cell death studies and associated drug designs have focused on the induction of apoptosis.^11^ The translational importance of such studies is evidenced by the development of new drugs such as venetoclax that inhibit Bcl-2 family proteins to initiate apoptosis. While effective in palliative AML care in combination with a hypomethylating agent,^62^ venetoclax has not displaced the use of ara-C in the frontline, curative chemotherapy of AML.

The primary mechanism of cell death by ara-C treatment has long been thought to be its incorporation into DNA and induction of apoptosis.^5^^,^^6^^,^^7^^,^^8^ However, active killing of AML primary isolates by ara-C can be caspase independent,^10^ and caspase-3 activation trends failed to predict ara-C and anthracycline drug sensitivity of primary AML isolates from 42 patients.^17^ Overlapping features of apoptosis and parthanatos, including externalization of phosphatidylserine with retention of plasma membrane integrity (Figure 1A), has likely resulted in a historical overuse of the term apoptosis to describe the active killing of cancer cells by ara-C and other chemotherapeutic drugs that can selectively induce parthanatos.^40^^,^^41^

The frontline drug combination of ara-C and idarubicin can induce either parthanatos or apoptosis, depending on the specific AML cells being evaluated. Results with OCI-AML2 cells are consistent with previous studies^7^ showing that certain leukemia cell lines can undergo classical apoptosis following ara-C treatment. However, OCI-AML3 cells exhibited caspase-independent cell death (Figures 1B and 2F) and distinctive parthanatos features, including rapid PAR accumulation (Figure 1E), AIF translocation (Figures 1F and 1G), and the production of large DNA fragments at the nuclear periphery (Figures 2A and 2B). We also observed a lack of mitochondrial transition pores and hyperpolarization of mitochondrial membranes (Figures 1C and 1D). These two features have not been previously reported during parthanatos and are in stark contrast to the opening of mitochondrial pores and loss of mitochondrial membrane potentials associated with apoptosis. The induction of parthanatos in OCI-AML3 cells was a specific result of ara-C and idarubicin treatment because the addition of CPT or AT-101 resulted in classical apoptosis features including caspase-3 activation and globular nuclear morphologies (Figures 1B and 2C). Likewise, PBMCs from healthy human donors also exhibited either parthanatos or apoptosis features upon addition of ara-C/idarubicin or AT-101, respectively (Data S2). These results demonstrate that white blood cell death mechanisms are not “hardwired” but rather depend on the specific drug(s) being added. In the future, more complete killing of leukemic stem cells and lower incidences of drug-resistant/relapsed AML cases^63^ may be achieved by regimens that stimulate both apoptosis and parthanatos. For example, the development of new targeted drugs that directly activate parthanatos by inhibiting putative parthanatos suppressors such as PARG or ARH3^35^^,^^64^^,^^65^^,^^66^ could be combined with venetoclax.^62^

Previous clinical interest in parthanatos has been mostly focused on neurodegenerative conditions, diabetes, heart disease, liver toxicity, ischemic-reperfusion injury, and stroke^39^^,^^67^ where inhibition of parthanatos is desirable.^68^ In the context of cancer, only a small handful of recent studies have evaluated the stimulation of parthanatos by chemotherapeutic drugs, including oxaliplatin in oral squamous cell carcinoma cell cultures^40^ and alkylating agents in HeLa cells.^41^ Here, we report that among the 10 AML cell lines tested, three of the cell lines (OCI-AML3, MOLM-14, and THP-1) exhibited distinctive parthanatos features upon incubation with ara-C and idarubicin. These three cell lines generally consistent with a greater extent of hematopoietic differentiation as compared with the seven cell lines lacking parthanatos features (Figure S4A).^55^^,^^56^ The same trend was observed when comparing M1/M2 versus M4/M5 primary AMLs, as well as HSPCs (CD34^+^) versus PBMCs (CD34^−^) from healthy donors. Together, our results suggest that white blood cells with a greater extent of hematopoietic differentiation are more likely to exhibit both parthanatos features upon incubation with ara-C and idarubicin.

We hypothesized that the presence/absence of parthanatos features in individual patients with AML could be associated with different clinical outcomes following intensive 7+3 chemotherapy. After assessing numerous assays for detecting unique features of parthanatos in primary AML cells that remain viable for only ∼3 days *ex vivo*, we selected cell nuclei morphologies and toxicity rescue by olaparib for conducting a prospective study of primary AML patient samples in a single-blind fashion, where we made parthanatos assignments before receiving any clinical information about each patient. The first selected feature, measuring the impact of PARP-1 on drug sensitivity, is upstream of programmed cell death signaling.^27^ The second feature, ring-shaped cleaved chromatin, is an outcome of MIF-mediated cleavage of single-stranded DNA^34^ to give a relatively small number of large chromatin fragments (Figure 2B). Our results are consistent with an early report showing that primary leukemia cells treated with various nucleoside-based drugs failed to give apoptotic nucleosomal ladders but rather gave large-molecular-weight DNA fragments.^38^ Our findings are further supported by studies of human breast epithelial cell lines MCF-10A and SKBR-3 undergoing parthanatos, which also exhibited DNA fragments distributed in a ring-shaped pattern.^69^ These same types of patterns were also observed in primary blood samples taken from eight patients with M4/M5 AML undergoing a standard 7+3-day continuous i.v. infusion of ara-C at 200 mg/m^2^ and idarubicin at 12 mg/m^2^ *in vivo* (Data S3).

Here, we report that primary white blood cells from approximately 50% of M4/M5 AML (18 of 39 tested) exhibited two distinctive parthanatos features upon treatment with ara-C and idarubicin *ex vivo*. The parthanatos-positive patients with AML had an overall 2-year survival of 66%–67% in contrast to the 17%–28% survival of the parthanatos-negative subgroup (Table 1). Multivariate analyses are consistent with the hypothesis that parthanatos is an independent risk factor for patient outcome (Table S2). Our cohort (n = 39) exhibited the same well-known trends for risk factors associated with age, gender, ELN risk group, and blast load as reported by previous studies,^4^^,^^59^ and it was large enough to reach statistical significance for parthanatos status (HR = 0.367, p = 0.046). Surprisingly, the primary isolates from the parthanatos-positive group exhibited the same or even lower average drug sensitivity *ex vivo* (EC_50_ = 13 ± 8 μM [−olaparib], EC_50_ = 21 ± 14 μM [+olaparib]) as compared with the parthanatos-negative subgroup (EC_50_ = 11 ± 6 μM [−olaparib], EC_50_ = 11 ± 7 μM [+olaparib]; Table S2). These results suggested that the mechanism of cell death can have more prognostic value than the absolute drug sensitivity exhibited by primary isolates *ex vivo*.

The presence/absence of olaparib rescue and the presence/absence of the nuclear DNA ring morphologies for the 39 M4/M5 AML isolates tested *ex vivo* were highly correlated with one another (χ^2^_(3)_ = 29.1, p ≤ 0.0001) and thus may serve as a robust *ex vivo* prognostic assay for ara-C administration. Patients with newly diagnosed AML who are predicted to have poor responsiveness toward intensive 7+3 chemotherapy could be prescribed alternative therapies such as a hypomethylating agent,^70^^,^^71^ Bcl-2 inhibitor,^62^ and/or FLT3 kinase inhibitor.^72^ Improved AML patient stratification is especially important given the devastating side effects of intensive chemotherapy including “tumor lysis syndrome,” which occurs in ∼20% of treated patients and is responsible for approximately 2%–5% of AML mortalities.^73^ Such treatment-related mortality further highlights the heterogeneity of the disease and the need for new diagnostic strategies that can identify which patients are unlikely to respond to intensive chemotherapy.

The diagnostic profiling of *PARP1* may provide future means for patient stratification. Previous studies reported that *PARP1* overexpression predicts poor AML patient survival.^29^^,^^30^^,^^61^ Here, we observe this same trend only in FLT3-mutated M4/M5 AML (Figure 5E). FLT3 mutation is a well-known prognostic marker associated with especially poor survival in ∼25% of AML cases,^74^ and it is known to drive *PARP1* overexpression.^75^ PARP inhibitors such as olaparib are currently being used to successfully treat solid tumors that are “addicted” to PARP-1 activity and therefore exhibit synthetic lethality upon PARP-1 inhibition.^76^ Our results further support the clinical evaluation of PARP inhibitors in FLT3-mutated AML. However, our results also contraindicate the use of PARP inhibitors during 7+3 chemotherapy since olaparib exhibited antagonistic effects toward the killing of both M1/M2 and M4/M5 AML cancer cells by ara-C and idarubicin (Tables S2 and S3).

Analyses of mRNA expression databases of AML patients receiving curative treatments with ara-C and idarubicin^60^ revealed that both low- and high-*PARP1*-expressing subgroups with FLT3 wild-type M4/M5 AML had inferior survival as compared with the median-expressing group (Figure 5C). Data from a smaller TCGA RNA-seq gene expression database for FLT3 wild-type M4/M5 AML (n = 56) reflected this same trend (Figure 5D). To evaluate the causal relationships between PARP-1 expression and drug sensitivity, we evaluated the impact of *PARP1* overexpression, RNA silencing, and PARP inhibition on drug sensitivity of OCI-AML2 and OCI-AML3 cells. Results for the OCI-AML3 cell line (FLT3 wild type, parthanatos competent) are consistent with the trends observed in the expressional databases and together provide evidence for our new hypothesis that basal/median expression levels of PARP-1 can support higher chemotherapeutic sensitivity as compared with PARP-1 overexpression or inhibition. This “double-edged sword” of PARP-1 activity was previously proposed for carcinogenesis.^77^ The presence of similar trends in other cancer types is currently unknown, but a recent report by Yang et al. demonstrated that PARP-1 activity is required for DNA alkylating agent-induced cell death of HeLa cell cultures,^41^ suggesting that parthanatos during cancer treatment is not a unique phenomenon.

### Limitations of the study

Some of the ring-shaped nuclei in cells taken from patients with AML being treated with ara-C and idarubicin may reflect non-cancerous cells (Data S3). Due to limited sample availability, the caspase-3 activation studies were conducted using only 10 primary isolates (Figure 4E) rather than the entire cohort of 39. Likewise, PARP-1 protein quantities versus patient survival could not be independently evaluated in the current study using western blot analyses; however, previous studies have demonstrated that *PARP1* mRNA quantities serve as a good proxy of PARP-1 protein quantities in both AML cell lines and primary AML cells.^29^ We also observed a correlation between *PARP1* mRNA and protein quantities in OCI-AML2 and OCI-AML3 cells treated with siRNA or a PARP1-expressing plasmid (Figures S1 and S2).

## STAR★Methods

### Key resources table

**Table:** 

REAGENT or RESOURCE	SOURCE	IDENTIFIER
**Antibodies**		

Biotin anti-mouse/human CD11b antibody (1:200)	BioLegend	Cat# 101203, RRID: AB_312786
Hu CD14 BUV496 MphiP9 50ug (1:200)	BD Biosciences	Cat# 741200, RRID: AB_2870760
Streptavidin APC-Cy™7 (1:200)	BD Biosciences	Cat# 554063, RRID: AB_10054651
Monoclonal rabbit anti-AIF antibody (1:50)	Abcam	Cat# 1020-1, RRID: AB_289587
Monoclonal mouse APC CD45 conjugate (1:50)	BioLegend	Cat# 982304, RRID: AB_2650648
Monoclonal mouse anti-PAR antibody (10H, 1:300)	AdipoGen	CAT# AG-20T-0001-C050
Goat-*anti*-mouse Alexa Fluor 488 antibody conjugate (1:100)	Thermo Fisher Scientific	Cat# A-21121, RRID: AB_2535764
Goat-*anti*-rabbit Alexa Fluor 555 antibody conjugate (1:50)	Thermo Fisher Scientific	Cat# A-21429, RRID: AB_2535850
Recombinant Anti-PARP1 antibody [E102] (rabbit, monoclonal, 1:1000)	Abcam	Cat# ab32138, RRID: AB_777101
Rabbit HRP-conjugated secondary antibody (1:2000)	Cell Signaling Technology	Cat# 7074, RRID: AB_2099233

**Biological Samples**		

Patient derived primary blasts	University Hospital Inselspital, Bern; University Hospital Zurich, Zurich; Erasmus University Medical Center, Rotterdam; Lady Davis Institute, Jewish General Hospital	N/A
Peripheral Blood Mononuclear Cells (PBMC) from healthy donors	University Hospital Inselspital, Bern; Lady Davis Institute, Jewish General Hospital	N/A
Human Bone Marrow CD34^+^ Progenitor Cells, Cryopreserved, 100,000	Lonza Biosciences	Cat# 2M-101

**Chemicals, peptides, and recombinant proteins**		

Cytosine β-D-arabinofuranoside	Sigma-Aldrich	Cat# C3350000
Idarubicin	Sigma-Aldrich	Cat# 1335701
AT101	Sigma-Aldrich	Cat# SML0433
Camptothecin (CPT)	Sigma-Aldrich	Cat# PHL89593
4′,6-diamidino-2-phenylindole (DAPI)	Sigma-Aldrich	Cat# D9542
Dimethyl sulfoxide (DMSO)	Sigma-Aldrich	Cat# D8418
Olaparib	Selleck Chemicals	Cat# S1060
Human TruStain FcX™	BioLegend	Cat# 422301
PBS	Thermo Fisher Scientific	Cat# 10010002
Bovine Serum Albumin (BSA)	Sigma-Aldrich	Cat# A7906
Paraformaldehyde	Alfa Aesar	Cat# J61899-AK
Triton X-100	Sigma-Aldrich	Cat# 93443
ProLong Gold Antifade mountant	Thermo Fisher Scientific	Cat# P10144
Z-VAD-FMK Caspase Inhibitor	Sigma-Aldrich	Cat# 627610
0.4% trypan blue stain	Gibco	Cat# 15250061
Mitotracker Red CMXRos staining solution	Thermo Fisher Scientific	Cat# M7512
RPMI	Sigma-Aldrich	Cat# R8758
MEM non-essential amino acid solution	Sigma-Aldrich	Cat# M7145
Penicillin-Streptomycin	Sigma-Aldrich	Cat# P4333
Fetal Bovine Serum (FBS)	Thermo Fisher Scientific	Cat# 10082147

**Oligonucleotides**		

PARP1 dsiRNA (human)	Integrated DNA Technologies	Design ID: hs.Ri.PARP1.13
Negative control dsiRNA	Integrated DNA Technologies	Cat# 51-01-14-03
Recombinant DNA		
Plasmid: pCMV3-untagged Negative Control Vector	Sino Biological	Cat# CV011
PARP cDNA ORF Clone, Human, untagged	Sino Biological	Cat# HG11040-UT

**Critical commercial assays**		

LIVE/DEAD fixable green dead cell stain kit	Thermo Fisher Scientific	Cat# L23101
Alexa Fluor 488 Annexin V/Apoptosis	Thermo Fisher Scientific	Cat# R37174
NucView 488 Caspase-3 Assay	Biotium	Cat# 30029
CF™ 488 TUNEL Assay	Biotium	Cat# 30063
MycoFluor™ Mycoplasma Detection Kit	Invitrogen	Cat# M7006
MitoProbe Transition Pore Assay	Thermo Fisher Scientific	Cat# M34153
Monarch Total RNA Miniprep Kit	New England Biolabs	Cat# T2010S
GoScript™ Reverse Transcription Mix	Promega	Cat# A2791
2x TaqMan™ Fast Advanced Master Mix	Thermo Fisher Scientific	Cat# 4444556
20x PARP1 TaqMan Expression Assay Hs00242302_m1 (FAM-MGB: S - 250 rxns)	Thermo Fisher Scientific	Cat# 4331182
20x HPRT TaqMan Expression Assay Hs02800695_m1 (FAM-MGB: S – 250 rxns)	Thermo Fisher Scientific	Cat# 4331182
Pierce BCA Protein Assay kit	Thermo Fisher Scientific	Cat# 23227
Pierce™ ECL Western Blotting Substrate	Thermo Fisher Scientific	Cat# 32106

**Deposited data**		

GSE6891 Microarray dataset	Haemato Oncology Foundation for Adults in the Netherlands	Walter et al., 2015,^60^ dataset GSE6891
TGCA RNAseq dataset	Cancer Genome Atlas (TGCA) data portal	https://tcga-data.nci.nih.gov/tcga

**Experimental models: Cell lines**		

Human: OCI-AML2 cells	DSMZ	DSMZ Cat# ACC 99, RRID: CVCL_1619
Human: OCI-AML3 cells	DSMZ	DSMZ Cat# ACC-582, RRID: CVCL_1844
Human: HS-5 stromal cells	LGC Standards	ATCC Cat# CRL-11882, RRID: CVCL_3720
Human: MOLM-14 cells	DSMZ	DSMZ Cat# ACC 777, RRID: CVCL_7916
Human: MOLM-13 cells	DSMZ	DSMZ Cat# ACC 554, RRID: CVCL_2119
Human: THP-1 cells	ATCC	ATCC Cat# TIB-202, RRID: CVCL_0006
Human: PL-21 cells	DSMZ	DSMZ Cat# ACC 536, RRID: CVCL_2161
Human: HL-60 cells	ATCC	ATCC CAT# CCL-240, RRID: CVCL_0002
Human: MV4-11 cells	DSMZ	DSMZ Cat# ACC 102, RRID: CVCL_0064
Human: ME-1 cels	DSMZ	DSMZ Cat# ACC 537, RRID: CVCL_2110
Human: ML-2 cells	DSMZ	DSMZ Cat# ACC 15, RRID: CVCL_1418

**Walter Software and algorithms**		

GraphPad Prism 9.2.0	GraphPad	RRID: SCR_002798https://www.graphpad.com/scientific-software/prism/
Fiji	PMID: 22743772	Schindelin et al., 2012;^78^ RRID: SCR_002285https://imagej.net/software/fiji/
LAS AF 2.6.0	Leica Microsystems	RRID: SCR_013673 https://www.leica-microsystems.com/products/microscope-software/p/leica-las-x-ls/
FlowJo 10.0.8	FlowJo LLC	RRID: SCR_008520 https://www.bdbiosciences.com/en-ca/products/software/flowjo-v10-software
ImageJ 1.47c	National Institutes of Health	https://imagej.nih.gov/ij/

**Other**		

Lymphoprep™	Axis-Shield	Cat# 1114545
Thermo Shandon Cytospin 3 Centrifuge	Marshall Scientific	Cat# TH-CYTO3
Leica SP5 Mid UV-VIS	Leica Microsystems	RRID: SCR_018714
TCS Leica SP8 Multiphoton (63× oil immersion objective, NA 1.4)	Leica Microsystems	RRID: SCR_018852
Spark microplate reader	Tecan	N/A
LSR II Fortessa	BD Biosciences	RRID: SCR_018655
4D-Nucleofector X Unit	Lonza Biosciences	Cat# AAF-1003X
Biopectrometer Fluorescence	Eppendorf	N/A
CFX96™ Real-Time System	Bio-Rad	Cat# 1845097
C1000 Touch™ Thermal Cycler	Bio-Rad	Cat# 1851196
Amersham™ Imager 600 system	GE Healthcare Life Sciences	N/A

### Resource availability

#### Lead contact

Further information and requests for resources and reagents should be directed to and will be fulfilled by Nathan W. Luedtke (nathan.luedtke@mcgill.ca).

#### Materials availability

This study did not generate new unique reagents.

### Experimental model and subject details

#### Human subjects

Primary cell samples and later, following analysis, the corresponding patient data were obtained from Francois E. Mercier (Department of Medicine, McGill University), Peter J. M. Valk (Department of Hematology, Erasmus University Medical Center), Alexandre P. A. Theocharides (Department of Medical Oncology and Hematology, University of Zurich) and Thomas Pabst (Department of Medical Oncology, University Hospital Inselspital). This study was approved by a decision of the local ethics committee in Berne, Switzerland (decision number #207/14), and Zurich, Switzerland (EK-ZH-NR: 2009-0062/1 and BASEC-NR: 2018-00539). All experiments were performed in accordance with the regulations of the Institutional Review Board of the Jewish General Hospital [11–047]. All donors provided written, informed consent.

#### Primary and stem cell cultures

Mononuclear cells were isolated from the peripheral blood of AML patients at the time of diagnosis or from healthy donors using a Lymphoprep (Axis-Shield) and were frozen in media with 10% DMSO as a cryoprotectant and stored in liquid nitrogen. AML cells were purified from the bone marrow using Ficoll density gradient centrifugation and cryopreserved in media containing 10% fetal calf serum (FCS) and 10% DMSO. For experiments, primary cell samples were thawed and transferred to a 50 mL falcon tube containing 200 μL of PBS/DNase (2000 U/mL) solution (Sigma-Aldrich). The cryotube was rinsed with 1 mL of thawing medium (5% FBS/PBS/DNase (20 U/mL)). Afterward, 18 mL of thawing media was added in a stepwise manner over 4 min. The cells were centrifuged for 15 min at 300 x g and resuspended in 5 mL media. Cell viability was determined with Trypan Blue staining (Sigma-Aldrich) and analyzed by Cell Counter Model R1 (Olympus). For the olaparib rescue experiments, blood cells were co-cultured with the feeder cell line HS-5.^58^ Accordingly, the HS-5 cells were seeded in 24-well plates at a density of 1 x 10^5^/500 μL media per well 24 h prior to seeding of the primary blood cells at a density of 2–6 x 10^5^/500 μL media per well. Human bone marrow CD34^+^ progenitor cells (Lonza Biosciences) were thawed, transferred to a 50 mL falcon tube containing 9 mL of thawing medium (10% FBS/PBS/DNase (20 U/mL)) and centrifuged at 300 x g at room temperature for 10 min. Cells were resuspended in culture media and incubated overnight before application in downstream experiments.

#### Cell lines

OCI-AML3, OCI-AML2, MOLM-13, MOLM-14, PL-21, MV4-11, ME-1, and ML-2 cell lines were obtained from the Leibniz Institute DSMZ-German Collection of Cell Cultures GmbH. THP-1 and HL-60 cell lines were obtained from the American Type Culture Collection (ATCC). HS-5 stromal cells were obtained from LGC Standards. All cell lines were monthly tested for mycoplasma infections using the MycoFluor Mycoplasma Detection Kit (Invitrogen). AML cell lines and primary cells were cultured in RPMI at 37°C in a humidified incubator with 5% CO_2_. The media was supplemented with 1% MEM non-essential amino acid solution (Sigma-Aldrich), 50,000 units of penicillin and 50 mg of streptomycin per liter (Sigma-Aldrich). Additionally, 10% FBS (Thermo Fisher Scientific) was added to the media for OCI-AML2, MOLM-13, MOLM-14, MV4-11 and THP-1 cells while 20% was supplemented for OCI-AML3, ME-1, ML-2, PL-21, HL-60, HS-5, and primary cells. Cell lines were grown to confluency and passaged every 2–4 days. Cell viability was determined with Trypan Blue staining (Sigma-Aldrich) and analyzed by Cell Counter Model R1 (Olympus).

### Method details

#### Differential staining cytotoxicity assay

Cells were seeded in 6-well plates at a density of 5 x 10^5^/2 mL media per well. After incubation with relevant doses of drugs, cells were transferred to centrifuge tubes, washed with PBS, and stained for live and dead cells with the LIVE/DEAD fixable green dead cell stain kit (Thermo Fisher Scientific) according to the instructions of the manufacturer. In the case of primary AML isolates, the cells were stained at the same time with the APC conjugated surface marker antibody CD45 after a 5 min blocking step with Human TruStain FcX solution (20x, BioLegend) diluted in 1% BSA/PBS. Fixation was performed in 300 μL paraformaldehyde (cell lines: 3.7% in PBS, primary cells: 2% in PBS) for 15 min at RT. Cells were washed with PBS and 1% BSA/PBS. As a next step, cells were permeabilized in 200 μL 0.2% Triton X-100/PBS for 15 min on ice and washed with PBS. Afterward, cells were blocked with 1% BSA/PBS for 15 min at RT. Primary antibodies were diluted in 1% BSA/PBS to their appropriate concentrations and cells were incubated with the solution for 2 h at RT. Cells were washed with PBS, 1% BSA/PBS and finally incubated in 50 μL of 1% BSA/PBS containing the appropriate secondary antibodies for 30 min at RT. After two more washing steps with PBS, the cells were resuspended in 500 μL DAPI solution (5 μM in PBS) and incubated overnight at 4°C. Cell suspensions were analyzed by LSR II Fortessa (BD Biosciences) and the results were evaluated with the FlowJo software (version 10.0.8, FlowJo LLC).

#### Cell imaging

For further analysis with confocal microscopy, the cells were transferred to microscope slides using a Thermo Shandon Cytospin 3 Centrifuge, coverslipped with ProLong Gold Antifade mountant (Thermo Fisher Scientific) and analyzed by confocal laser scanning microscopy using a Leica SP5 Mid UV-VIS or TCS Leica SP8 Multiphoton (63× oil immersion objective, NA 1.4) or Leica SP8 LSM (Leica HC PL CS2 63X/1.4 NA oil objective). Images were analyzed with LAS AF 2.6.0 (Leica Microsystems) and ImageJ 1.47c (National Institutes of Health).

#### Annexin V staining

Cells were seeded in 6-well plates at a density of 1.2 x 10^6^/2.5 mL media per well. After incubation with 1 μM (OCI-AML2) or 10 μM (OCI-AML3) of ara-C for 24 h, phosphatidyl serine externalization was determined with Dead Cell Apoptosis Kit with Annexin V Alexa Fluor 488 and propidium iodide (Thermo Fisher Scientific) according to the instructions of the manufacturer.

#### Caspase-3 assay

Cells were seeded in 6-well plates at a density of 5 x 10^5^/2 mL media per well and incubated with 1 μM (OCI-AML2) or 10 μM (OCI-AML3) of ara-C for 8 h. Activity of caspase-3 was detected with NucView 488 Caspase-3 Assay kit (Biotium) according to the instructions of the manufacturer.

#### Caspase inhibition assay

OCI-AML2 (passage range of p7 to p9) and OCI-AML3 (passage range of p2 to p4) cells were passaged 48 h prior to experiments. On day one, cells were seeded on 24-well plates (Sarstedt) in a five (treatment group) x three (replicate) design at a density of 1 x 10ˆ5 cells/500 μL culture media per well. All wells were then directly treated with 10 μM/well of Z-VAD-FMK Caspase Inhibitor (Sigma-Aldrich) dissolved in DMSO (Bio Basic) and incubated for 24 h at 37°C, 5% CO_2_. Subsequently, 1 mM of cytarabine (Sigma-Aldrich) dissolved in ultra-pure water (Bio Basic) and 100 μM idarubicin (Sigma-Aldrich) dissolved in DMSO (Bio Basic) were directly added to each well in the five treatment groups, to final concentration/well of: 1) 0 : 0 μM; 2) 0.5 : 0.03 μM; 3) 1 : 0.06 μM; 4) 5 : 0.3 μM; 5) 15 : 0.9 μM. Cytarabine and Idarubicin treated cells were then incubated for 24 h at 37°C, 5% CO_2_. Trypan blue staining was used to measure cell viability using Spark microplate reader and dual chamber disposal cell chip (Tecan) by adding equal volumes of 0.4% trypan blue stain (Gibco) and sample. Percent viability was analyzed using GraphPad Prism 9.2.0. Three biological replicates were performed for each experiment.

#### Mitochondrial membrane potential assay

Cells were seeded in 6-well plates at a density of 5 x 10^5^/2 mL media per well and incubated with 1 μM (OCI-AML2) or 10 μM (OCI-AML3) of ara-C for 24 h. Cells were transferred to centrifuge tubes and stained with 500 μL Mitotracker Red CMXRos staining solution (200 nM in PBS, Thermo Fisher Scientific) for 30 min at 37°C. After one washing step with PBS, cells were fixed with 300 μL paraformaldehyde (3.7% in PBS) for 15 min at RT. The cells were washed twice with PBS and resuspended in 500 μL DAPI solution (5 μM in PBS) and incubated overnight at 4°C. Cell suspensions were analyzed by LSR II Fortessa.

#### MitoProbe Transition Pore Assay

Cells were seeded in 6-well plates at a density of 5 x 10^5^/2 mL media per well and incubated with 1 μM (OCI-AML2) or 10 μM (OCI-AML3) of ara-C for 24 h. Mitochondrial permeability transition pore opening was measured using MitoProbe Transition Pore Assay Kit (Thermo Fisher Scientific) according to the instructions of the manufacturer.

#### TUNEL assay

Cells were seeded in 6-well plates at a density of 5 x 10^5^/2 mL media per well and incubated with 1 μM (OCI-AML2) or 10 μM (OCI-AML3) of ara-C for 24 h. The CF 488 TUNEL Assay (Biotium) was performed according to the instructions of the manufacturer.

#### *PARP1* overexpression and siRNA silencing

OCI-AML2 (passage range of p7 to p9) and OCI-AML3 (passage range of p2 to p6) cells were seeded 48 h prior to experiments. On first day of experiment, 200,000 cells were spun down and resuspended in 90 μL of full culture media for each condition on a 96-well plate (Sarstedt). Experiments consisted of pCMV3-untagged Negative Control Vector plasmid (Sino Biological) and PARP cDNA ORF Clone, Human, untagged plasmid (Sino Biological) treatment groups for each cell line with three replicates per treatment group. For each well, 0.1 μg of plasmid DNA (Sino Biological), 0.8 μL of ViaFect reagent (Promega) and 10 μL of Opti-MEM 1x (Gibco) was incubated for 15 min at room temperature. Subsequently, 10 μL of this DNA/reagent mix was added to the 90 μL of cells in each well. The cells were then incubated for 24 h at 37°C, 5% CO_2_. The next day, cells were transferred to 12 well plates (Sarstedt) with 1 mL culture media per well and incubated for an additional 48 h at 37°C, 5% CO_2_. Transfected cells were then analyzed by RT-qPCR and Western blot.

#### Nucleofection of *PARP1* siRNA

PARP1 dicer-substrate siRNA (dsiRNA) and scrambled non-targeting negative control dsiRNA (Integrated DNA Technologies) were introduced into 1 × 10^6^ cells in the logarithmic growth phase using nucleofection (Lonza 4D-Nucleofector X Unit: AAF-1003X, 16-well nucleocuvette strips). Cells were nucleofected according to the manufacturer’s guidelines; SF solution and program DN-100 were used for the nucleofection of OCI-AML2 cell line, and SF solution and program EH-100 was used for the nucleofection of OCI-AML3 cell line. Nucleofected cells were validated for gene silencing using RT-qPCR and Western blot. Furthermore, nucleofected cells were treated with escalating doses of ara-C and Idarubicin in a 17:1 ratio for 24 h and assessed for cell viability using the LIVE/DEAD fixable green dead cell stain kit (Thermo Fisher Scientific) and flow cytometry according to the instructions of the manufacturer.

#### RNA extraction and RT-qPCR

Total RNA was extracted from transfected OCI-AML2 and OCI-AML3 cells with Monarch Total RNA Miniprep Kit (New England BioLabs) and quantified using a BioSpectrometer Fluorescence (Eppendorf) according to manufacturer’s instruction. GoScript Reverse Transcription Mix, Oligo(dT) (Promega) was used for reverse transcription of 5 ng/μL of RNA to cDNA in a 20 μL reaction. 2x TaqMan Fast Advanced Master Mix (Thermofisher Scientific) and 20x TaqMan Gene Expression Assays (Thermofisher Scientific) were used for real-time quantitative PCR analysis to quantify expression levels of *PARP1* and *HPRT1*. qPCR was conducted using CFX96 Real-Time System (Bio-Rad), and C1000 Touch Thermal Cycler (Bio-Rad). ΔΔCt values were calculated to compare the expressions by the (2^-ΔΔCt^) method.

#### Western Blot

Cells were lysed using ice-cold RIPA buffer supplemented with protease inhibitor and centrifuged for 15 min at 16,000 xg at 4°C. Pierce BCA Protein Assay kit (Thermofisher Scientific) was used to quantify protein according to the manufacturer’s protocol. Equal volume of 2x Laemmli sample buffer was mixed with protein sample and heated at 70°C for 10 min. 20 μg of total protein was loaded onto 10% separating and 6% stacking SDS-PAGE gel. Protein expression was confirmed by 4°C overnight incubation of PVDF membrane containing protein of interest with Recombinant Anti-PARP1 antibody [E102] (rabbit, monoclonal, 1:1000; Abcam) followed by 1 h incubation with anti-rabbit HRP-conjugated secondary antibody (1:2000; New England BioLabs) at room temperature. Immunoblots were visualized by Pierce ECL Western Blotting Substrate (Thermofisher Scientific) according to the manufacturer’s protocol, and the chemiluminescent signals were captured using CCD camera-based imager, Amersham Imager 600 system (GE Healthcare Life Sciences). Relative integrated density values were calculated by Fiji ImageJ 1.53c (National Institutes of Health) and GraphPad Prism 9.4.1.

#### Flow cytometric assay for cell surface differentiation markers

OCI-AML2 (passage range of p7 to p9) and OCI-AML3 (passage range of p2 to p4) cells were seeded in 6-well plates at a density of 4 x 10^5^/2 mL media per well and incubated with escalating doses of ara-C and idarubicin in 17:1 ratio for 24 h. 1 μM olaparib pretreatment was done 24 h prior to ara-C/Idarubicin treatment. Drug-treated cells were centrifugated at 500 xg for 5 min, and the pellet was washed twice with PBS supplemented with 2% FBS. Primary antibodies Biotin anti-mouse/human CD11b antibody (BioLegend) and BUV496 Mouse Anti-Human CD14 antibody (BD Biosciences) were diluted in 2% FBS/PBS to their appropriate concentrations and cells were incubated on ice in the dark with 100 μL of the solution for 25 min. Cells were washed with 2% FBS/PBS, and cells treated with the primary antibody Biotin anti-mouse/human CD11b (BioLegend) were incubated on ice in the dark in 100 μL of 2% FBS/PBS containing the secondary antibody Streptavidin APC-Cy7 (BD Biosciences) for 25 min. All conditions were washed and stained for live and dead cells with the LIVE/DEAD fixable green dead cell stain kit (Thermo Fisher Scientific) according to the instructions of the manufacturer. Cells were transferred to FACS tubes, analyzed by LSR II Fortessa (BD Biosciences), and the results were evaluated with the FlowJo software (version 10.0.8, FlowJo LLC).

### Quantification and statistical analysis

#### Confocal microscopy

Confocal images of AML cell lines in Figures 1G and 2A and 2C, as well as primary AML blasts in Figures 4A–4C and Data S1, S3, S4, and S5 were captured with a Leica SP5 Mid UV-VIS or TCS Leica SP8 Multiphoton (63× oil immersion objective, NA 1.4). Confocal images of AML cell lines in Figures 3A–3D and Figures S3A–S3D, PBMCs in DataSet S2 as well as CD34^+^ HSPCs in Figure S4D were captured with a Leica SP8 LSM using Leica HC PL CS2 63X/1.4 NA oil objective (#506350). The images were collected using a Leica HyD detector with emission detection windows set between 413nm–565nm (Gain at 50). Hoechst 33342 excitation was performed using a Coherent Cameleon Vision 2 multiphoton laser, wavelength at 720 nM, power at 2.3 W (3.0%). All images were analyzed with LAS AF 2.6.0 (Leica Microsystems) and ImageJ 1.47c (National Institutes of Health).

#### Flow cytometry analysis

Flow cytometry was used to quantify olaparib rescue by measuring % viability of ara-C and idarubicin treated AML blasts and PBMC with or without olaparib pre-treatment. Primary AML blast cells and PBMC from healthy donors were labeled in suspension and analyzed by LSR II Fortessa (BD Biosciences), and the results were evaluated with the FlowJo software (version 10.0.8, FlowJo LLC). Forward scatter from the appropriate laser was used as the trigger signal for an event. A dual parameter dot plot of forward and side scatter was used to identify intact cells from debris. Forward scatter and side scatter height and width were used to identify single cells and exclude doublets. Sidelight scattering and CD45 immunostaining were used to analytically distinguish AML cells from other types of cells. Bound dye conjugated antibody was excited with the appropriate wavelength laser and emission was detected by a photomultiplier tube with a corresponding band-pass filter (log peak). LIVE/DEAD fixable green dead cell stain (Thermo Fisher Scientific) was excited with the 488 nm laser and its emission detected by a photomultiplier tube (PMT) with a corresponding band-pass filter (log peak). Data was also collected on all other available detectors for reference. FACSDiva software was used for event acquisition and analysis. Results were then evaluated with the FlowJo software (version 10.0.8, FlowJo LLC).

#### Survival analysis

All Kaplan-Meier survival estimates in Figures 5A–5E and S6 and S7 were generated using the product-limit method of Kaplan and Meier in GraphPad Prism. Kaplan-Meier survival estimates were compared through univariate analysis by a Mantel-Cox test and log rank hazard ratios (Tables 1 and 2; Figures 5A–5E; S6 and S7). Multivariate analysis was done by Cox proportional hazard model and four well-established risk factors as co-variates: age, gender, genetic risk group, and blast load in the bone marrow (Table 2; Figure 5A).

#### mRNA expression analysis of parthanatos- and apoptosis-associated genes

mRNA expression data from clinical trials organized by the Haemato Oncology Foundation for Adults in the Netherlands (GSE6891)^60^ were analyzed, as well as RNAseq analysis of AML samples from the Cancer Genome Atlas (TCGA) Research Network taken from the Cancer Genome Atlas data portal (https://tcga-data.nci.nih.gov/tcga). Boxplots with whiskers (25–75% interquartile range) illustrate relative mRNA levels and comparisons between AML FAB groups M1 & M2 vs. M4 & M5 were tested using nonparametric Mann-Whitney U test. The nonparametric Kruskal-Wallis test was used to compare boxplots in Figure 5F. All results were plotted, and statistical tests were carried out with GraphPad Prism versions 8 and 9. P-values <0.05 are reported as statistically significant and are depicted as follows throughout the manuscript: ∗p < 0.05, ∗∗p < 0.01, ∗∗∗p < 0.001, ∗∗∗∗p < 0.0001. This information can also be found in the figure legends. Statistical tests used as well as value and definition of n can also be found in the figure legends.

#### Chi-Square test

To determine the correlation between the presence/absence of toxicity rescue by olaparib and the presence/absence of DNA ring morphologies after 24 h incubations with ara-C/Idarubicin (Figure 4D), a chi-Squared test was done in a 4x2 contingency table with the presence or absence of four outcomes: ring (+)/olaparib rescue (+); ring (−)/olaparib rescue (−); ring (+)/olaparib rescue (−); and ring (−)/olaparib rescue (+). Statistical analysis was done on GraphPad Prism 9.4.1.

## Data Availability

De-identified raw and processed patient data is available in Document S1. Figures S1–S7, Table S2. Profiles of patients with AML FAB subtypes M4 and M5 in this study, related to Figures 4 and 5A and Data S4 and S5, Table S3. Profiles of patients with AML FAB subtypes M1 and M2 in this study (n = 8) and summary of parthanatos features in their primary samples, related to Figure 4 and are listed in the key resources table. This paper also analyzes existing, publicly available data. The accession numbers for the datasets are listed in the key resources table. This paper does not report original code. Any additional information required to reanalyze the data reported in this paper is available from the lead contact Nathan W. Luedtke upon request.

## Supplemental information

Document S1. Figures S1–S7

Table S1. Summary of parthanatos features in PBMCs from healthy human donors, related to Figure 4 and Data S1 To determine the % of living cells, cells were pre-treated with olaparib (+Olap, 1 μM, 24 h) or carrier only (-Olap), followed by a 17:1 mixture of ara-C (5 μM) + idarubicin (0.3 μM) for 24 h. Cells were then treated with a live/dead fixable stain, fixed, stained with DAPI and analyzed by flow cytometry or microscopy.

Table S2. Profiles of patients with AML FAB subtypes M4 and M5 in this study, related to Figures 4 and 5A and Data S4 and S5 Profile of all AML patients (n = 39) including their Overall survival (OS) and event-free survival (EFS) is shown. The % of living AML cancer cells is determined by isolating primary AML blast cells from each patient in the study and cells were treated *in vitro* with a variable concentration of ara-C (1–30 μM) in a fixed 17:1 ratio with idarubicin (0.06–1.8 μM) following pre-treatment with olaparib (+Olap, 1 μM, 24 h) or carrier only (-Olap). Cells were then treated with a live/dead fixable stain, fixed, stained with a CD45 antibody and DAPI. The blasts were distinguished from other cells by flow cytometry using side light scattering (SS+) and CD45 immunostaining (CD45^+^). Estimated EC50 values are given in terms of ara-C concentrations. The % values are normalized to the number of living cells in samples receiving no ara-C or idarubicin.

Table S3. Profiles of patients with AML FAB subtypes M1 and M2 in this study (n = 8) and summary of parthanatos features in their primary samples, related to Figure 4 Ring-shaped DNA fragmentation, and % of cells rescued by pre-treatment with olaparib (1 μM, 24 h) and treatment with ara-C (5 μM) + idarubicin (0.3 μM) were determined by confocal microscopy and flow cytometry, respectively.

Data S1. Flow cytometry and microscopy analyses of PBMCs from 10 healthy donors exhibiting two parthanatos features, related to Figure 4

Data S2. Microscopy analyses of PBMCs from 5 healthy donors upon addition of ara-C, idarubicin, a mixture of ara-C and idarubicin, or AT-101, related to Figures 2 and 4

Data S3. Microscopy analyses of PBMCs from 8 patients with AML (M4/M5) taken immediately before and during 7+3 induction chemotherapy with ara-C and idarubicin, related to Figure 4

Data S4. Flow cytometry and microscopy analyses of 18 AML patient samples exhibiting two parthanatos features, related to Figures 4 and 5A

Data S5. Flow cytometry and microscopy analyses of 21 AML patient samples exhibiting zero or one parthanatos feature, related to Figures 4 and 5A

Document S2. Article plus supplemental information
