# Supplementary material for: PARP-1 improves leukemia outcomes by inducing parthanatos during chemotherapy
Source: Cell Rep Med. 2023 Sep 7;4(9):101191. doi: 10.1016/j.xcrm.2023.101191 (PMC10518631; doi:10.1016/j.xcrm.2023.101191)
Supplement: Document S1. Figures S1–S7 [file mmc1.pdf]

Cell Reports Medicine, Volume *4*

Supplemental information

PARP-1 improves leukemia outcomes

by inducing parthanatos during chemotherapy

Bruktawit Maru, Alessandra Messikommer, Linhui Huang, Katja Seipel, Olivia Kovecses, Peter J.M. Valk, Alexandre P.A. Theocharides, Francois E. Mercier, Thomas Pabst, Maureen McKeague, and Nathan W. Luedtke

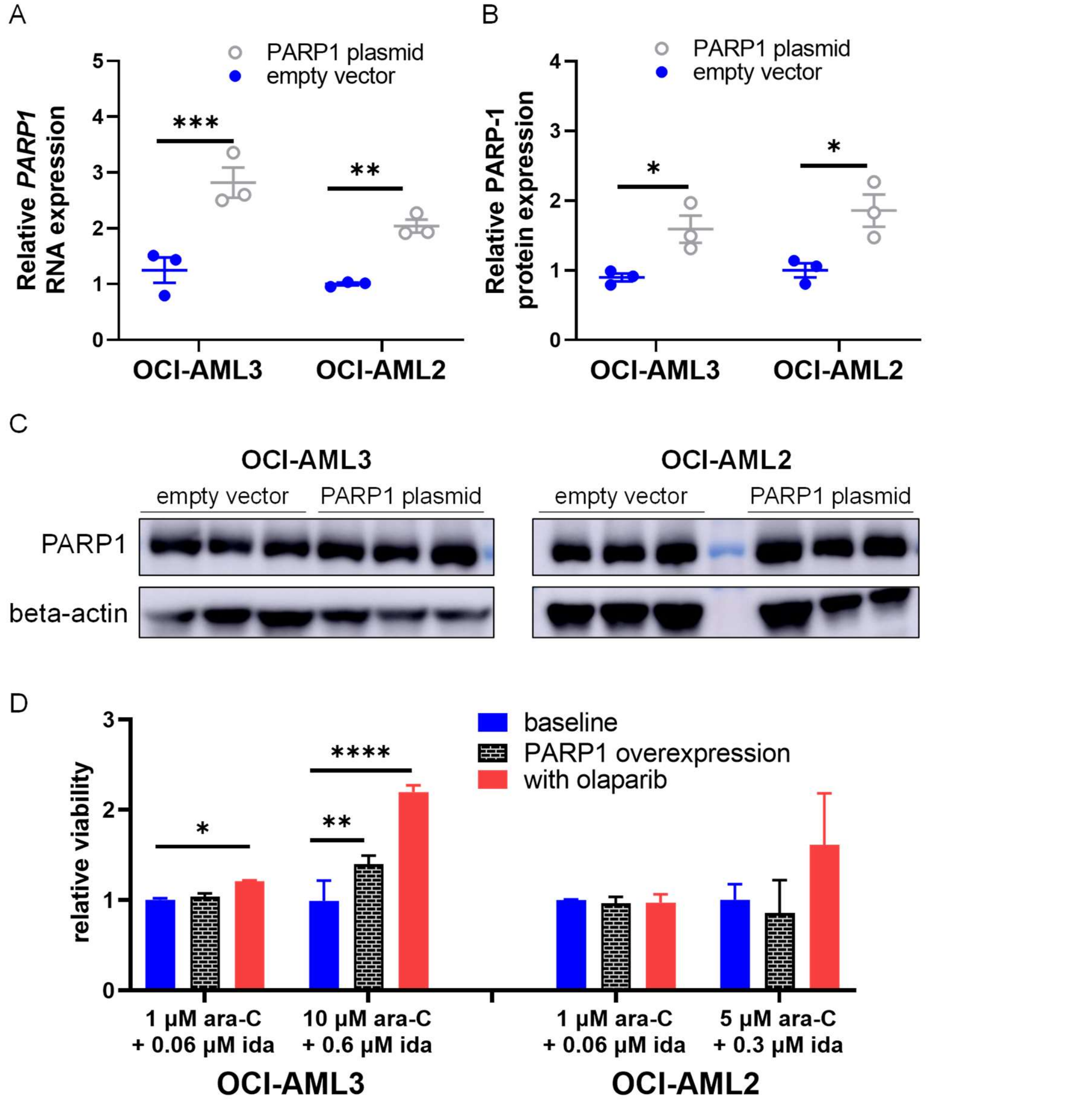

Supplemental Figure S1, related to Figure 2H: overexpression of PARP-1 and changes in drug sensitivity. **A)** Relative *PARP1* mRNA expression in OCI-AML2 and OCI-AML3 cells measured using qRT-PCR 24 h after PARP1 plasmid transfection; n = 3 biological replicates. **B)** Relative PARP-1 protein expression measured using Western blots 72 h after PARP1 plasmid transfection; n = 3 biological replicates. **C)** Western blot analysis of relative PARP1 protein expression 72 h after PARP1 plasmid transfection; n = 3 biological replicates. **A-B)** data represented as mean ± SEM; Statistical tests done using two-tailed student t-test, *p<0.05 **p<0.002 ***p<0.0002; Western blots normalized to beta-actin. **D)** Relative viability (# live cells in each experimental / control sample at the same drug concentration) of OCI-AML3 and OCI-AML2 cells receiving pre-treatment with a PARP-1 overexpressing plasmid for 72 h and/or 1 µM of olaparib for 24 h prior to addition of ara-C and idarubicin for 24 h. Data represented as mean ± SEM; Statistical tests were conducted using 2-way ANOVA with Tukey's multiple comparison test, *p<0.03, **p<0.002, ****p<0.0001. n = 3 technical replicates of 2 biological replicates.

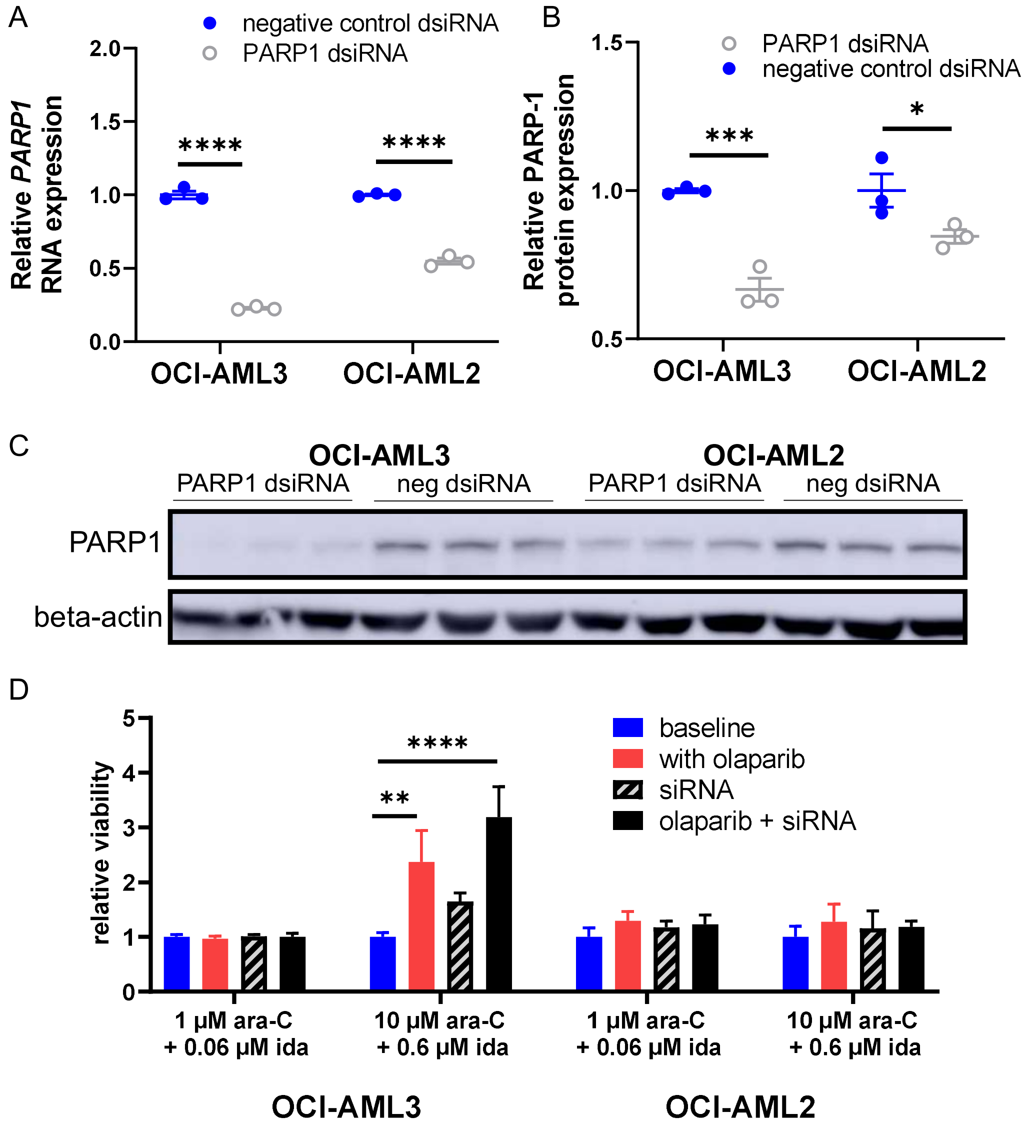

Supplemental Figure S2, related to Figure 2I: knockdown of PARP-1 and changes in drug sensitivity. A) Relative *PARP1* mRNA expression in OCI-AML2 and OCI-AML3 cells measured using qRT-PCR 24 h after siRNA transfection; n = 3 biological replicates. **B)** Relative PARP-1 protein expression measured using Western blots 72 h after siRNA transfection; n = 3 biological replicates. **C)** Western blot analysis of relative PARP1 protein expression 72 h after siRNA transfection; n = 3 biological replicates. **A-B)** Data represented as mean ± SEM; Statistical tests done using two-tailed student t-test, *p<0.05 ***p<0.0002 ****p<0.0001; Western blots normalized to beta-actin. **D)** Relative viability (# live cells in each experimental/control sample at same drug concentration) of OCI-AML3 and OCI-AML2 cells receiving pre-treatment with siRNA for 72 h and/or 1 µM of olaparib for 24 h prior to addition of ara-C and idarubicin for 24 h. Data represented as mean ± SEM; Statistical tests were conducted using 2-way ANOVA with Tukey's multiple comparison test, **p<0.002, ****p<0.0001. n = 3 technical replicates of 2 biological replicates.

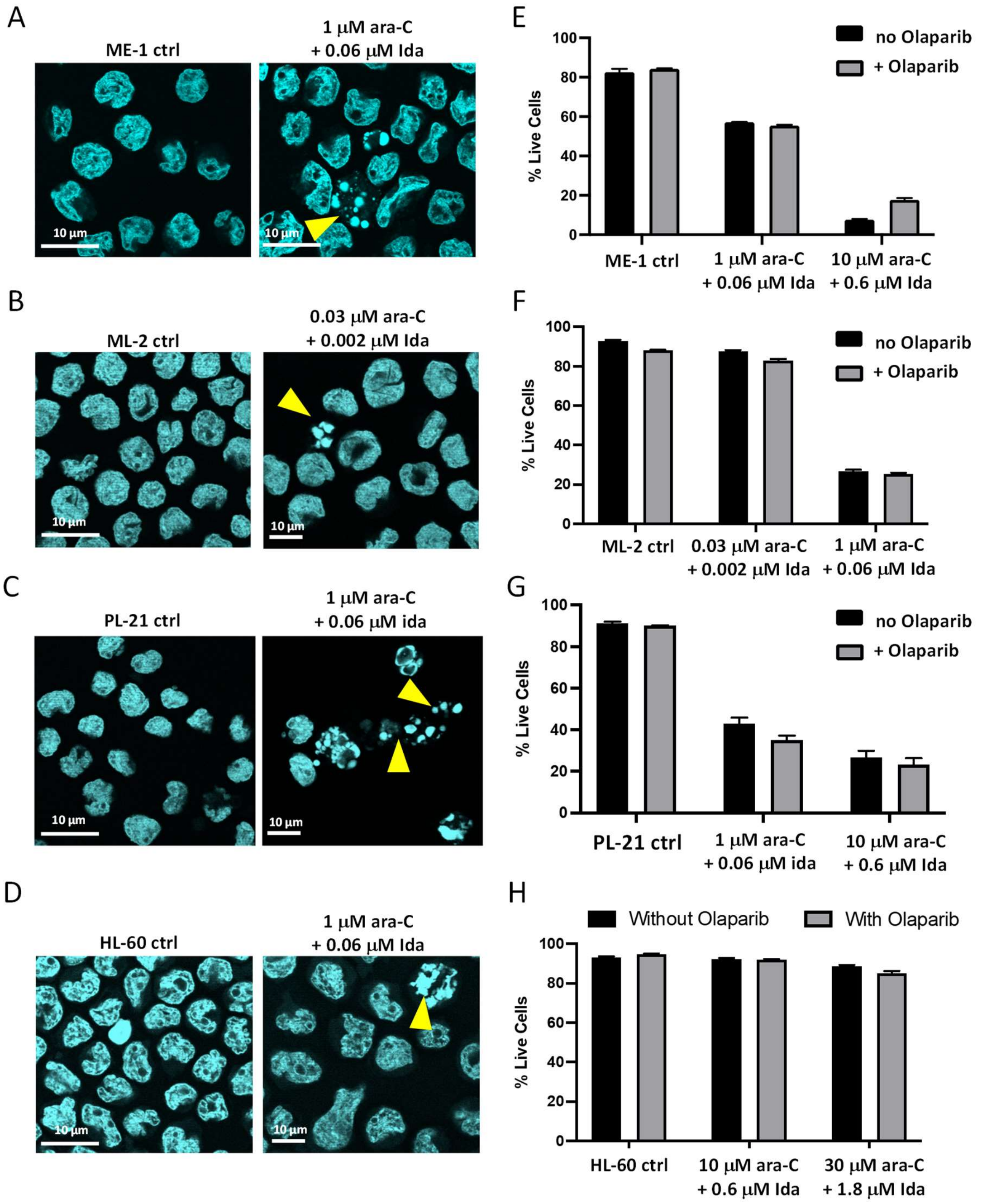

Supplemental Figure S3, related to Figure 3: analysis of four additional AML cell lines for parthanatos features following treatment with ara-C and idarubicin. A-D) Nuclear fragmentation pattern analysis using confocal microscopy 8 h after addition of ara-C and idarubicin: **A)** ME-1 (FAB M4); **B)** ML-2 (FAB M4); **C)** PL-21 (FAB M3); **D)** HL-60 (FAB M2). Red arrowheads indicate ring-shaped nuclear fragmentation. Yellow arrowheads indicate globular nuclear fragmentation. **E-H)** % live cells quantified by flow cytometry after 1 μM olaparib pre-treatment for 24 h followed by ara-C and idarubicin for 24 h: **E)** ME-1 (FAB M4); **F)** ML-2 (FAB M4); **G)** PL-21 (FAB M3); **H)** HL-60 (FAB M2). Statistical analysis was conducted using a 2-way ANOVA with Sidak multiple comparisons test, data represented as mean ± SD for n = 3 biological replicates *p<0.03, **p<0.002, ***p<0.0002, ****p<0.0001.

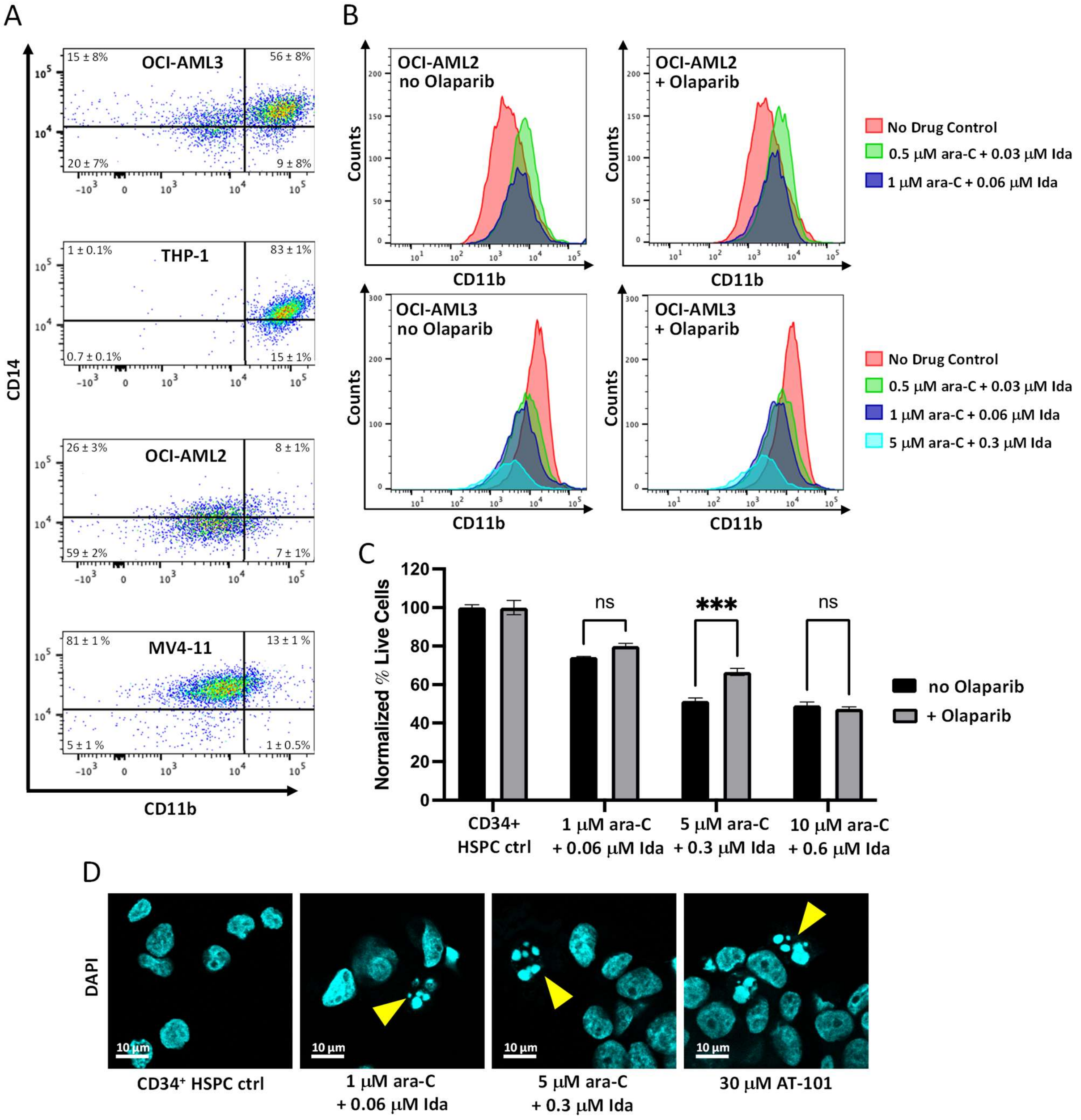

Supplemental Figure S4, related to Figures 3 and 4: immunofluorescent staining of four cell lines and absence of parthanatos features in CD34$^+$ hematopoietic stem cells (HSPCs). A) Analysis of CD14 and CD11b antigen immunofluorescent staining of four cell lines using flow cytometry. Data for one biological replicate shown with average +/- standard deviation values given for n = 3 biological replicates. **B)** Changes in CD11b immunofluorescent staining of OCI-AML2 and OCI-AML3 cells following a 24 h treatment with ara-C and idarubicin with or without pretreatment with 1 μM of olaparib for 24 h. Flow cytometry data were gated for live cells of one representivative biological replicate is shown for n = 3 collected. **C)** The % of living CD34$^+$ HSPCs quantified by flow cytometry following a 24 h treatment with ara-C and idarubicin with or without pretreatment with 1 μM of olaparib for 24 h. The statistical analysis was done with a 2-way ANOVA with Sidak multiple comparisons test, data represented as mean ± SD for n = 2 biological replicates ***$P < 0.0002$. **D)** Nuclear fragmentation pattern analysis of CD34$^+$ HSPCs treated with ara-C and idarubicin for 24 h. Yellow arrowheads indicate globular nuclear fragmentation patterns.

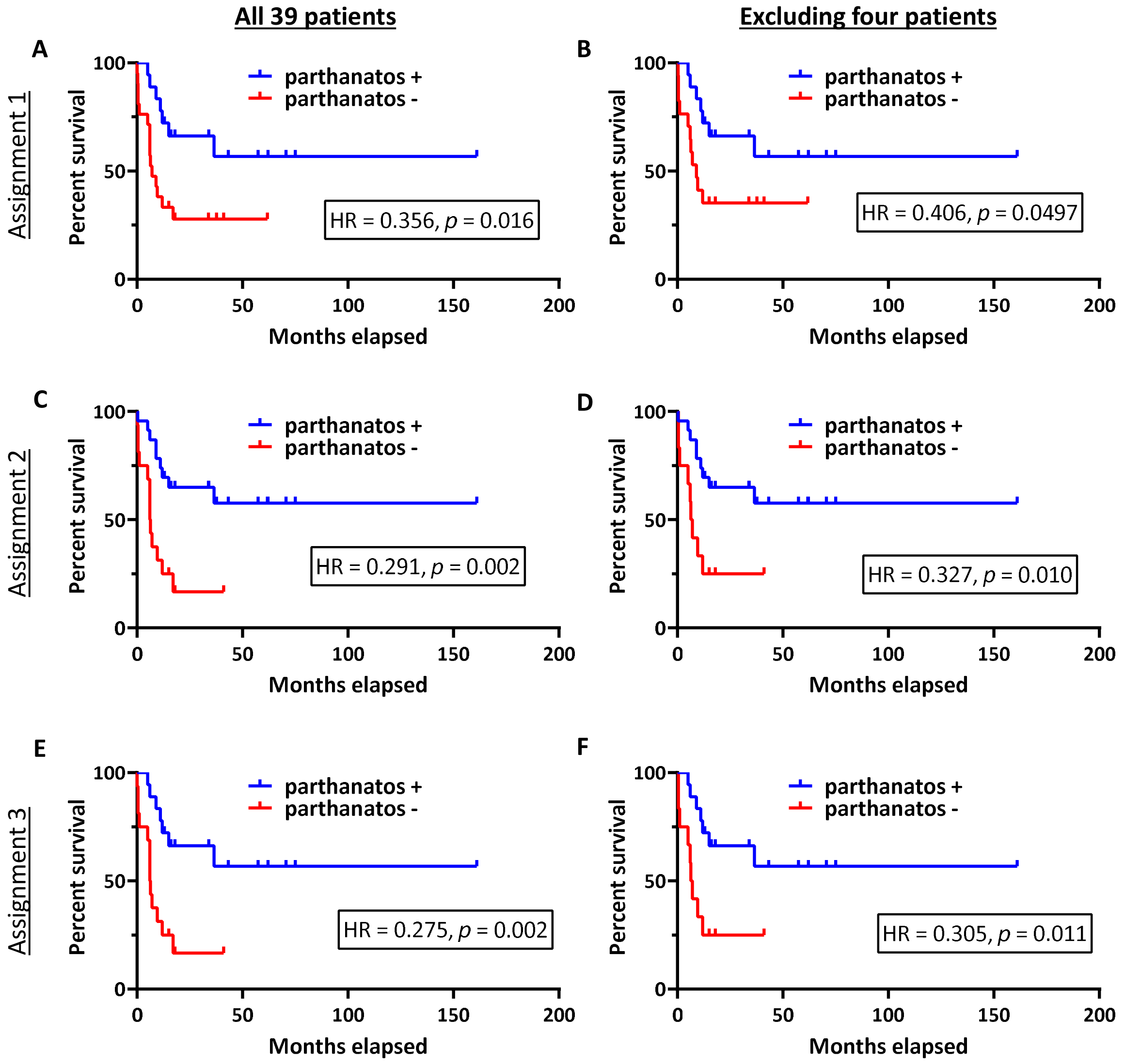

Supplemental Figure S5, related to Figure 5A and Tables 1-2: overall % survival (OS) of the parthanatos positive (+) versus negative (-) groups. A) Kaplan-Meier OS curves comparing +/- groups using Assignment 1: samples exhibiting both parthanatos features (n = 18) versus one and zero features (n = 21). **B)** Kaplan-Meier OS curves comparing +/- groups using Assignment 1 and excluding four patients who did not receive curative chemotherapy with ara-C. **C)** Kaplan-Meier OS curves comparing +/- groups using Assignment 2: samples exhibiting one and both parthanatos features (n = 23) versus zero features (n = 16). **D)** Kaplan-Meier OS curves comparing +/- groups using Assignment 2 and excluding four patients who did not receive curative chemotherapy with ara-C. **E)** Kaplan-Meier OS curves comparing +/- groups using Assignment 3: samples exhibiting both parthanatos features (n = 18) versus zero features (n = 16). **F)** Kaplan-Meier OS curves comparing +/- groups using Assignment 3 and excluding four patients who did not receive curative chemotherapy with ara-C.

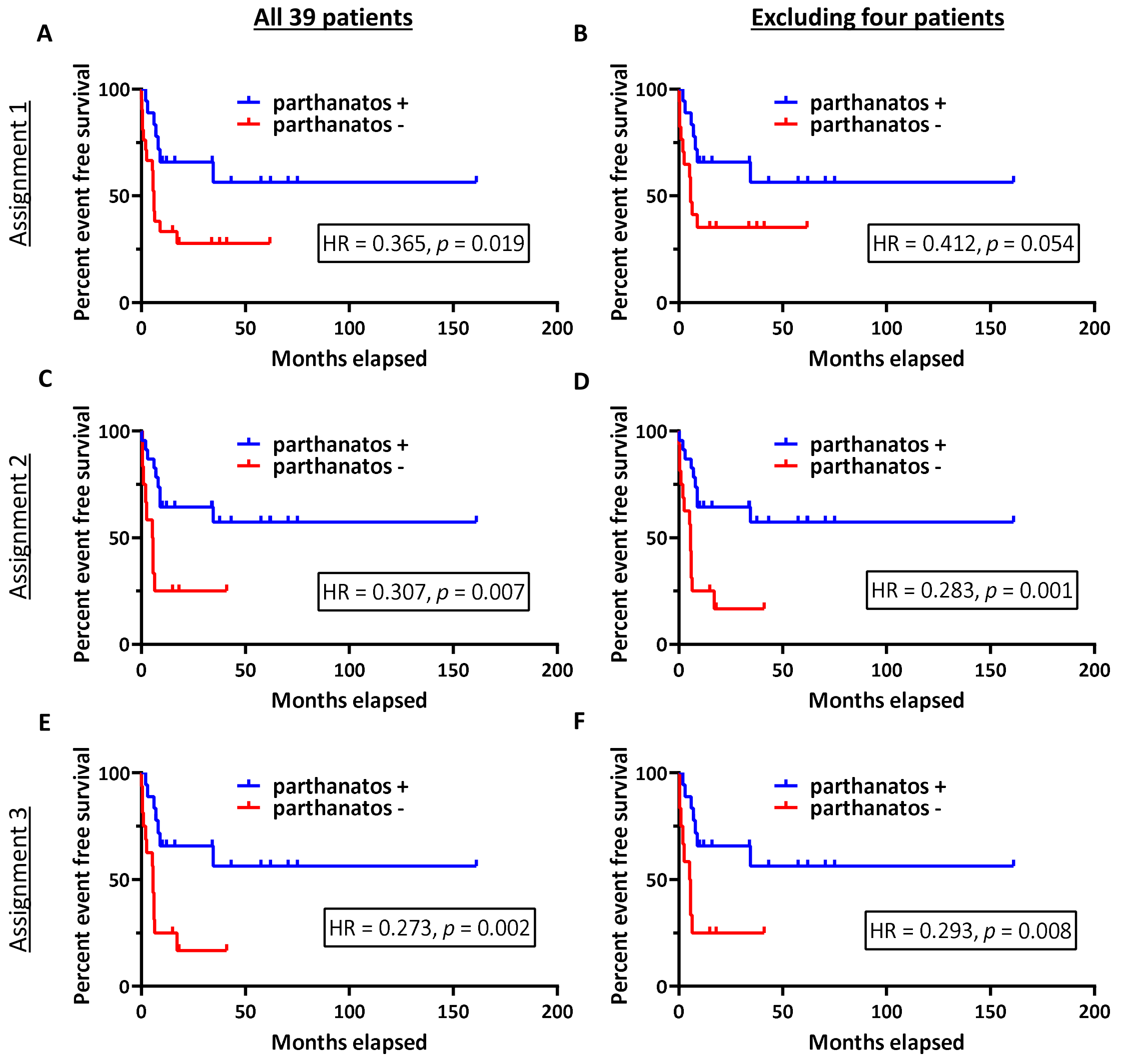

Supplemental Figure S6, related to Figure 5A and Table 1: event-free % survival (EFS) of the parthanatos positive (+) versus negative (-) groups. A) Kaplan-Meier EFS curves comparing +/- groups using Assignment 1: samples exhibiting both parthanatos features (n = 18) versus one and zero features (n = 21). **B)** Kaplan-Meier EFS curves comparing +/- groups using Assignment 1 and excluding four patients who did not receive curative chemotherapy with ara-C. **C)** Kaplan-Meier EFS curves comparing +/- groups using Assignment 2: samples exhibiting one and both parthanatos features (n = 23) versus zero features (n = 16). **D)** Kaplan-Meier EFS curves comparing +/- groups using Assignment 2 and excluding four patients who did not receive curative chemotherapy with ara-C. **E)** Kaplan-Meier EFS curves comparing +/- groups using Assignment 3: samples exhibiting both parthanatos features (n = 18) versus zero features (n = 16). **F)** Kaplan-Meier EFS curves comparing +/- groups using Assignment 3 and excluding four patients who did not receive curative chemotherapy with ara-C.

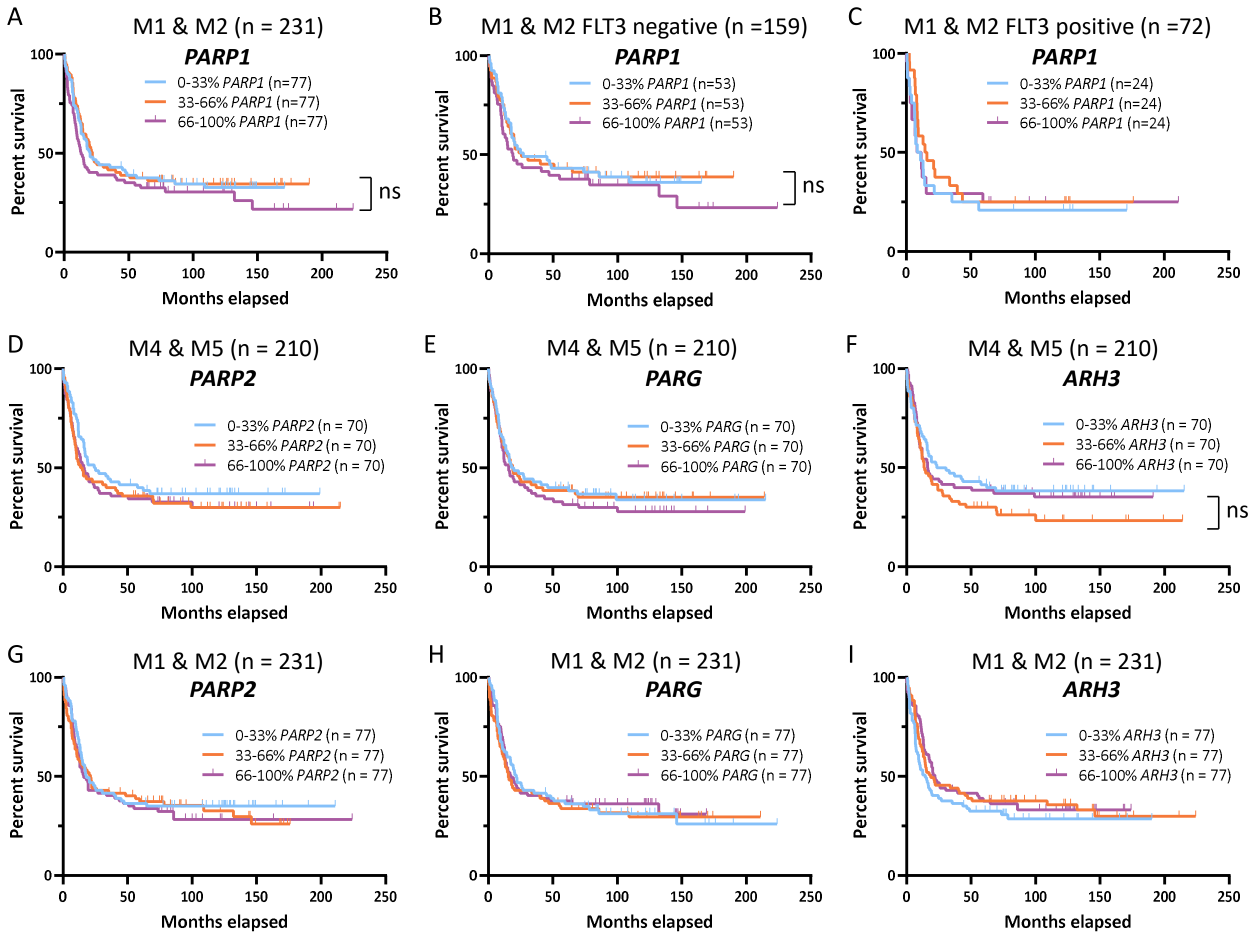

Supplemental Figure S7, related to Figure 5: analysis of mRNA expression in samples from AML patients receiving curative treatment with ara-C and idarubicin. mRNA expression data was taken from clinical trials organized by the Haemato Oncology Foundation for Adults (GSE6891, Walter et al., 2015). Patients were grouped into three equal groups according to the relative mRNA expression of each gene (low, 0-33%; medium, 33-66%; and high, 66-100%). Overall % survivals (OS) of each group were plotted using Kaplan-Meier survival estimates (Mantel-Cox log-rank and Mantel-Haenszel tests). **A)** *PARP1* (M1 & M2); **B)** *PARP1* (M1 & M2 FLT3 negative); **C)** *PARP1* (M1 & M2 FLT3 positive). **D)** *PARP2* (M4 & M5); **E)** *PARG* (M4 & M5); **F)** *ARH3* (M4 & M5); **G)** *PARP2* (M1 & M2); **H)** *PARG* (M1 & M2); **I)** *ARH3* (M1 & M2). ns = no statistically significant trends were observed.
