# Supplementary material for: PARP-1 improves leukemia outcomes by inducing parthanatos during chemotherapy
Source: Cell Rep Med. 2023 Sep 7;4(9):101191. doi: 10.1016/j.xcrm.2023.101191 (PMC10518631; doi:10.1016/j.xcrm.2023.101191)
Supplement: Data S1. Flow cytometry and microscopy analyses of PBMCs from 10 healthy donors exhibiting two parthanatos features, related to Figure 4 [file mmc5.pdf]

Supplementary Data Set 1: Flow cytometry and microscopy analyses of PBMCs from 10 healthy donors exhibiting two parthanatos features

a)

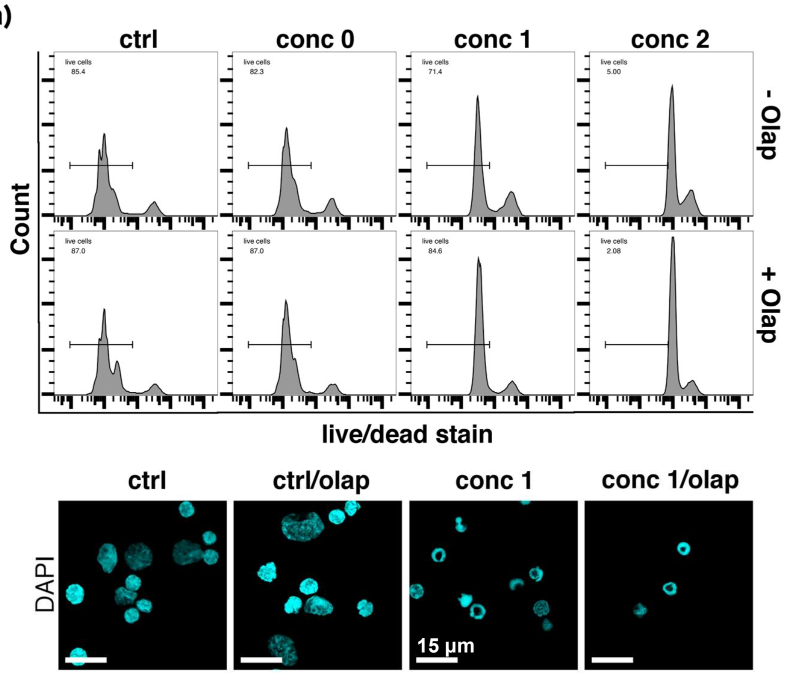

b)

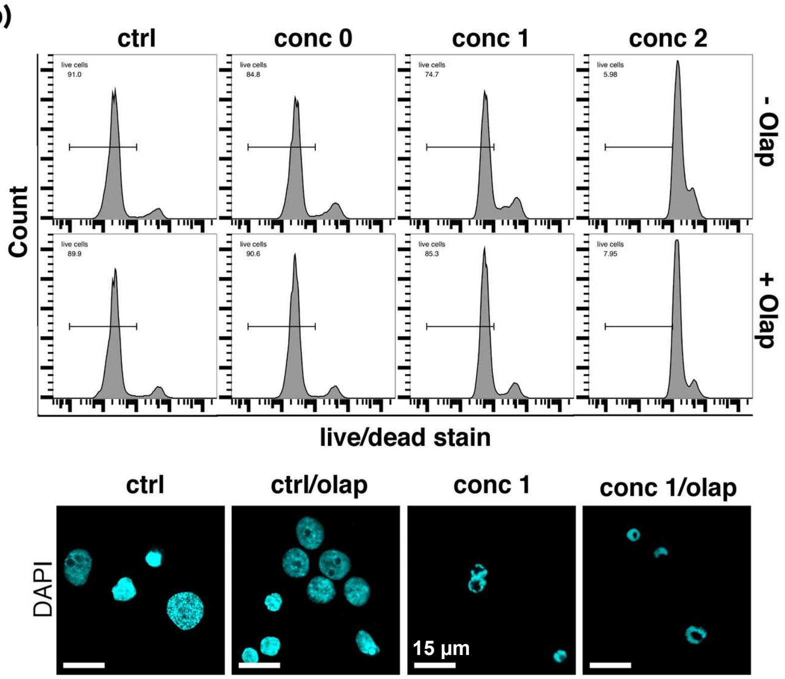

Parthanatos features in PBMCs from healthy donors according to toxicity rescue by Olaparib (Olap) and the presence of ring-shaped nuclei examined by DAPI staining. Primary cells: **a)** Human donor 1 (1951); **b)** Human donor 2 (1953). Pretreatment: 1 µM Olaparib o/n; drug treatment: 24 h. Conc 0: 1 µM ara-C + 0.06 µM ida, conc 1: 5 µM ara-C + 0.3 µM ida, conc 2: 15 µM ara-C + 0.9 µM ida.

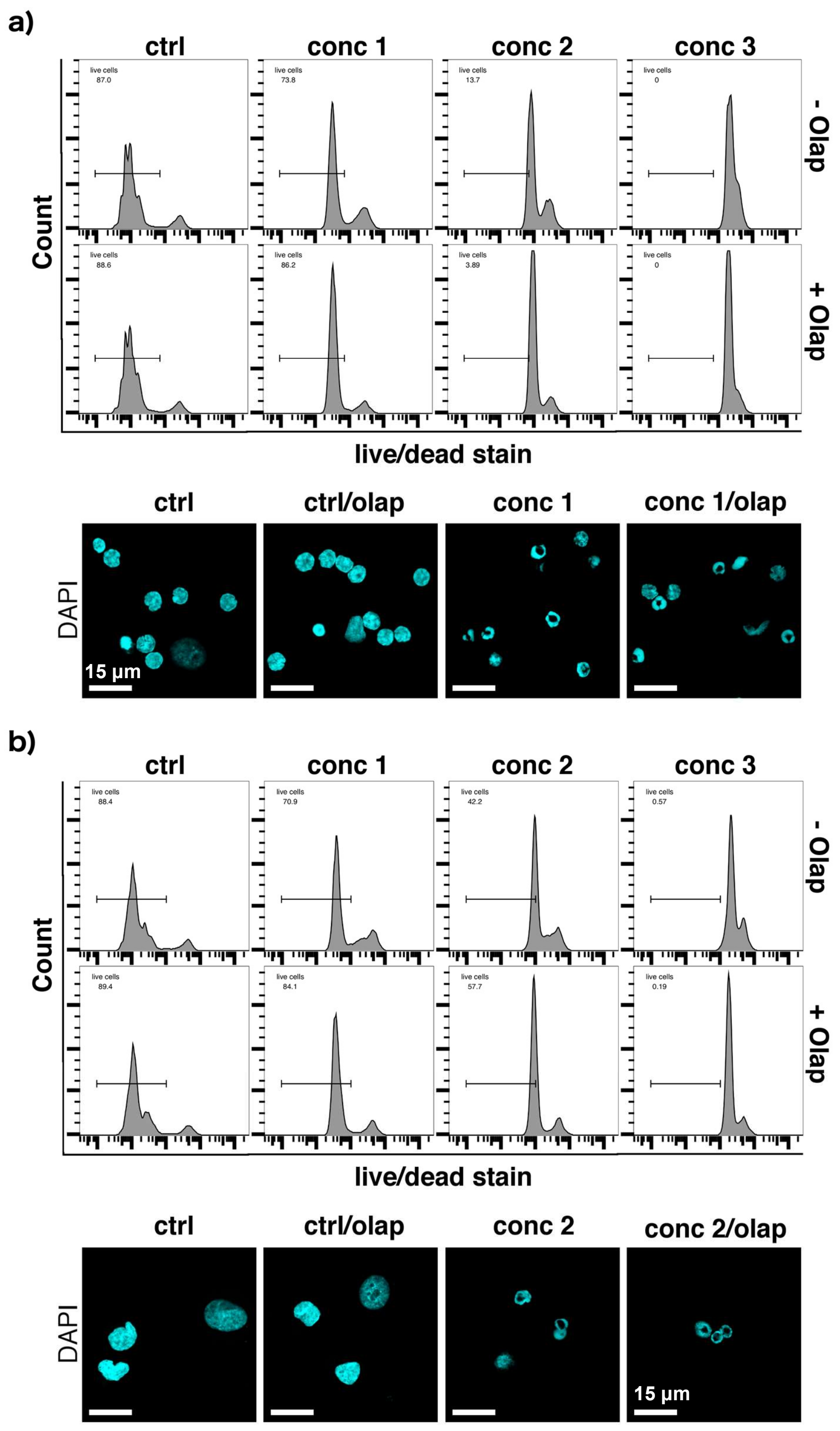

Parthanatos features in PBMCs from healthy donors according to toxicity rescue by Olaparib (Olap) and the presence of ring-shaped nuclei examined by DAPI staining. Primary cells: **a)** 3 / 1960 and **b)** 4 / 1970. Pretreatment: 1 µM Olaparib o/n; drug treatment: 24 h. Conc 1: 5 µM ara-C + 0.3 µM ida, conc 2: 15 µM ara-C+ 0.9 µM ida, conc 3: 30 µM ara-C + 1.8 µM ida.

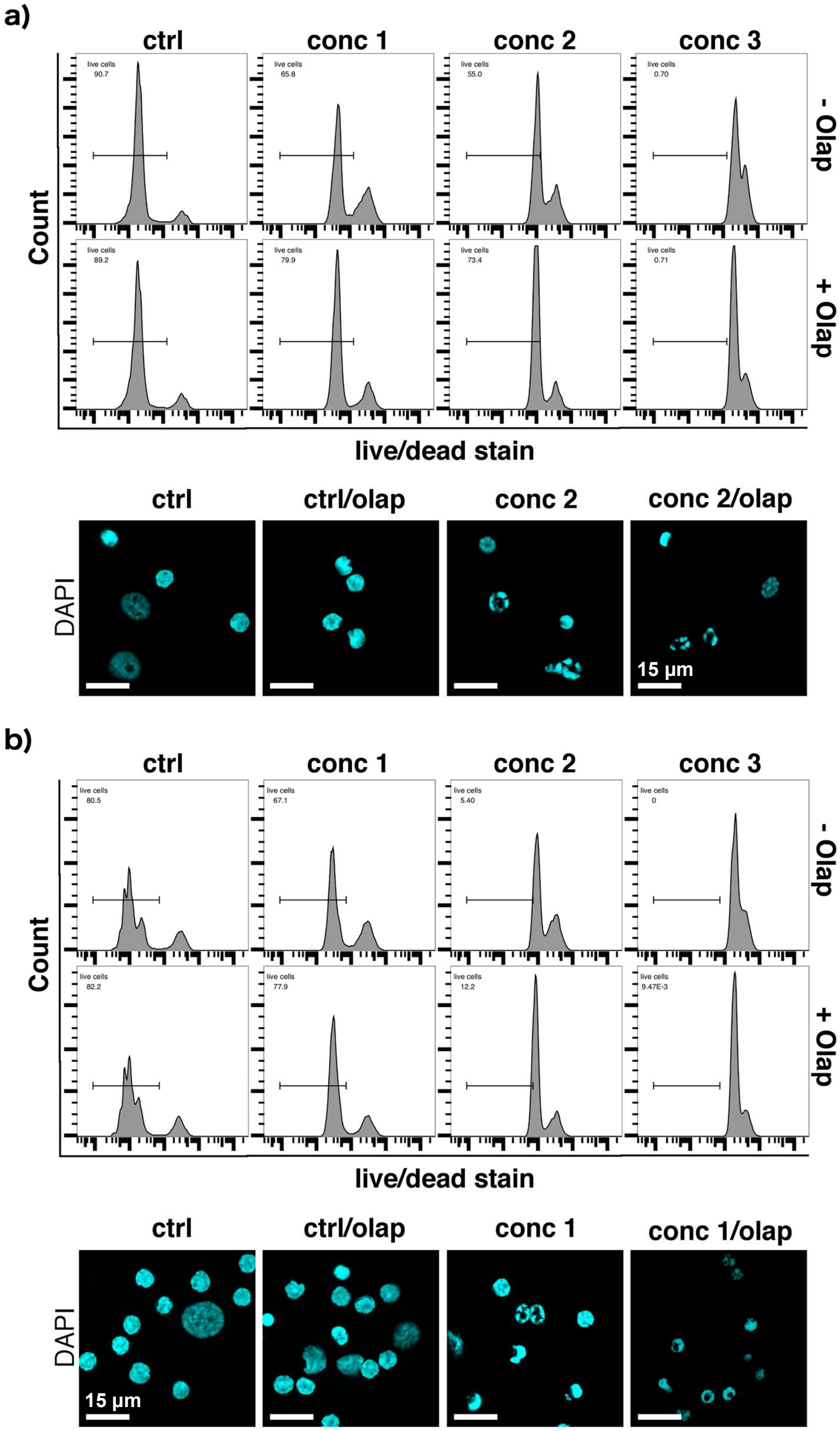

Parthanatos features in PBMCs from healthy donors according to toxicity rescue by Olaparib (Olap) and the presence of ring-shaped nuclei examined by DAPI staining. Primary cells: **a)** 5 / 1971 and **b)** 6 / 1973. Pretreatment: 1 µM Olaparib o/n; drug treatment: 24 h. Conc 1: 5 µM ara-C + 0.3 µM ida, conc 2: 15 µM ara-C + 0.9 µM ida, conc 3: 30 µM ara-C + 1.8 µM ida.

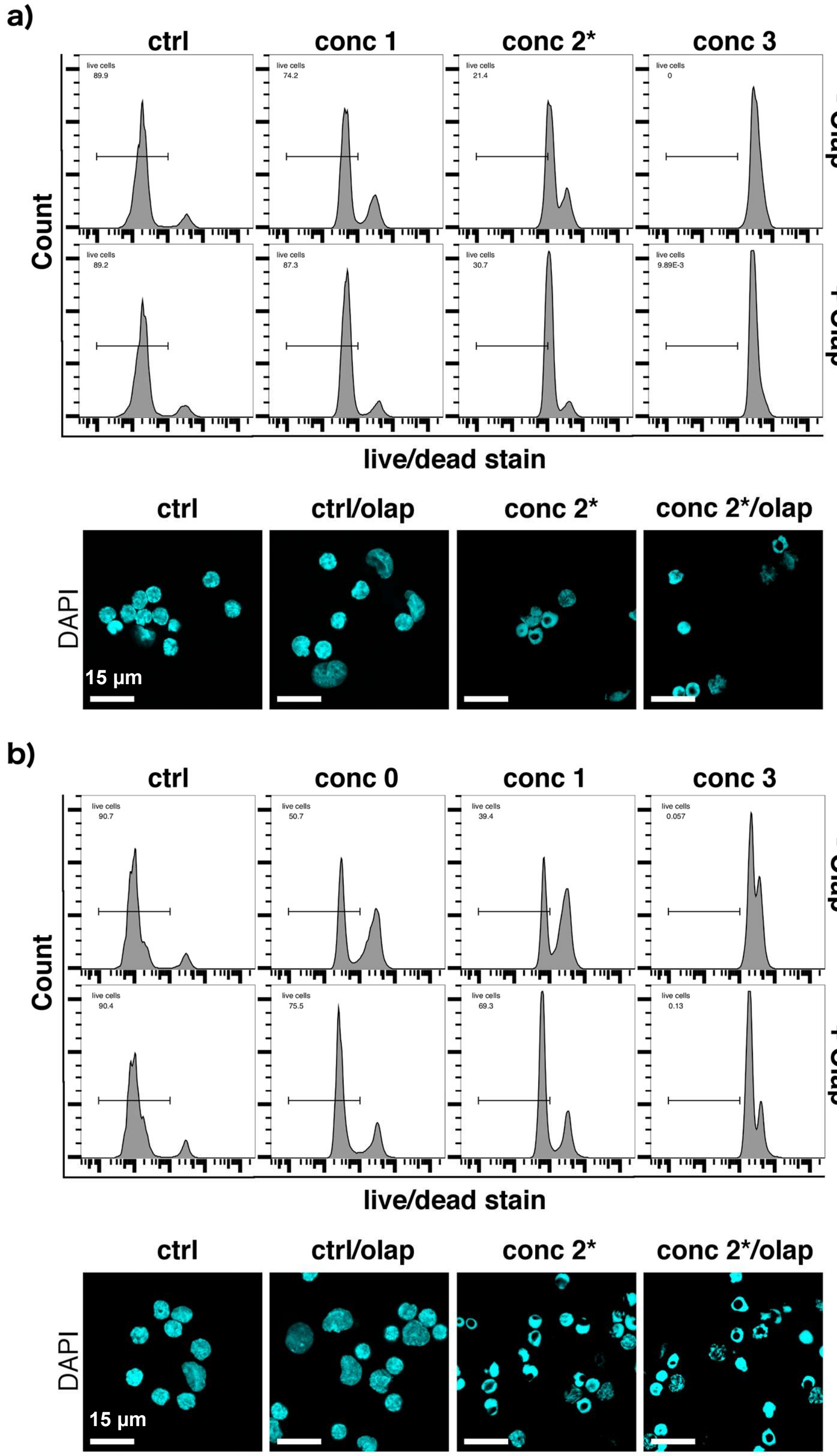

Parthanatos features in PBMCs from healthy donors according to toxicity rescue by Olaparib (Olap) and the presence of ring-shaped nuclei examined by DAPI staining. Primary cells: **a)** 7 / 1982 and **b)** 8 / 1986. Pretreatment: 1 µM Olaparib o/n; drug treatment: 24 h. Conc 0: 1 µM ara-C + 0.06 µM ida, conc 1: 5 µM ara-C + 0.3 µM ida, conc 2*: 10 µM ara-C + 0.6 µM ida, conc 3: 30 µM ara-C + 1.8 µM ida.

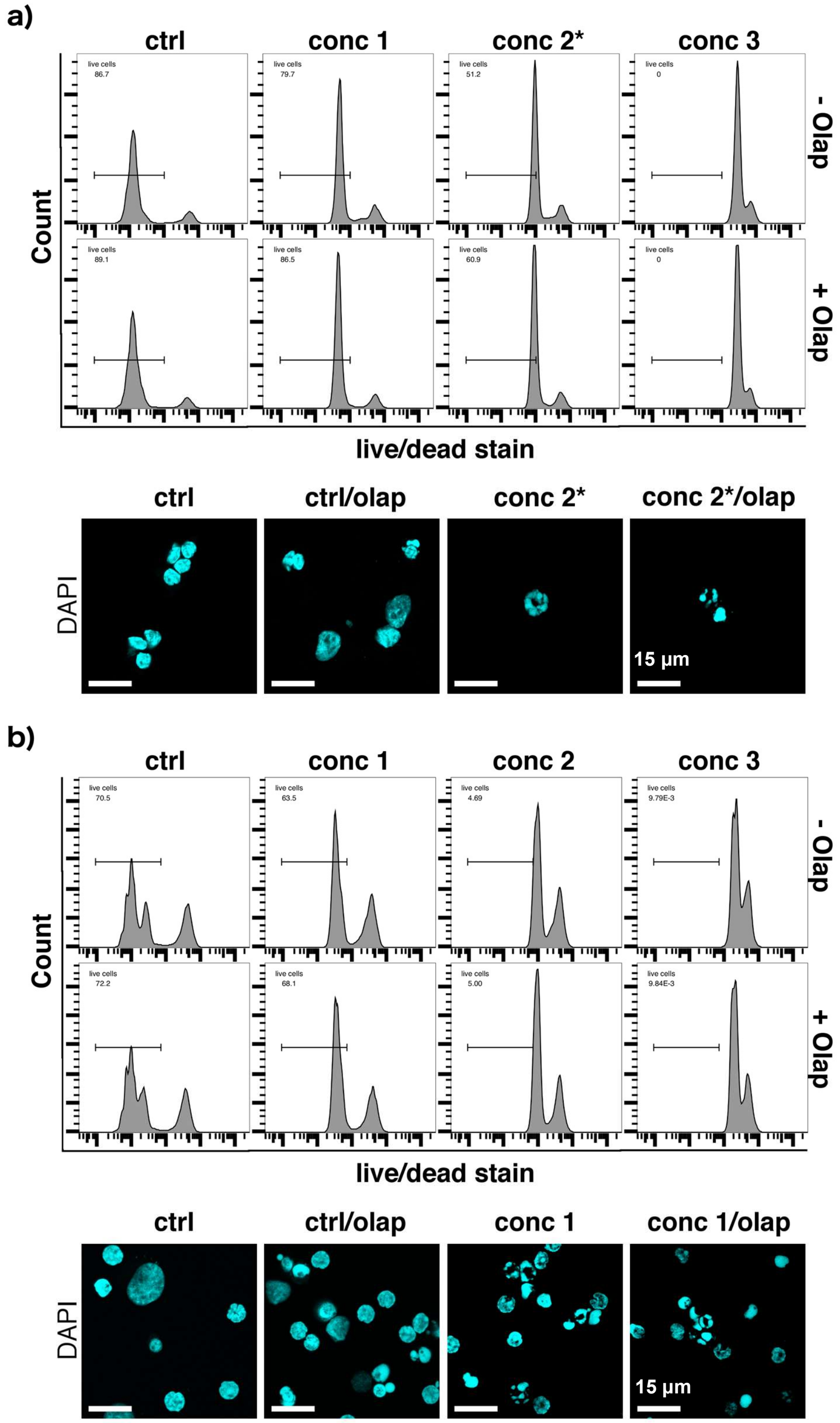

Parthanatos features in PBMCs from healthy donors according to toxicity rescue by Olaparib (Olap) and the presence of ring-shaped nuclei examined by DAPI staining. Primary cells: **a)** 9 / 1990 and **b)** 10 / E. Pretreatment: 1 µM Olaparib o/n; drug treatment: 24 h. Conc 1: 5 µM ara-C + 0.3 µM ida, conc 2*: 10 µM ara-C + 0.6 µM ida, conc 2: 15 µM ara-C + 0.9 µM ida, conc 3: 30 µM ara-C + 1.8 µM ida.
