# Supplementary material for: PARP-1 improves leukemia outcomes by inducing parthanatos during chemotherapy
Source: Cell Rep Med. 2023 Sep 7;4(9):101191. doi: 10.1016/j.xcrm.2023.101191 (PMC10518631; doi:10.1016/j.xcrm.2023.101191)
Supplement: Data S2. Microscopy analyses of PBMCs from 5 healthy donors upon addition of ara-C, idarubicin, a mixture of ara-C and idarubicin, or AT-101, related to Figures 2 and 4 [file mmc6.pdf]

Supplementary Data Set 2: Microscopy analyses of PBMCs from 5 healthy donors upon addition of ara-C, idarubicin, a mixture of ara-C/idarubicin, or AT-101.

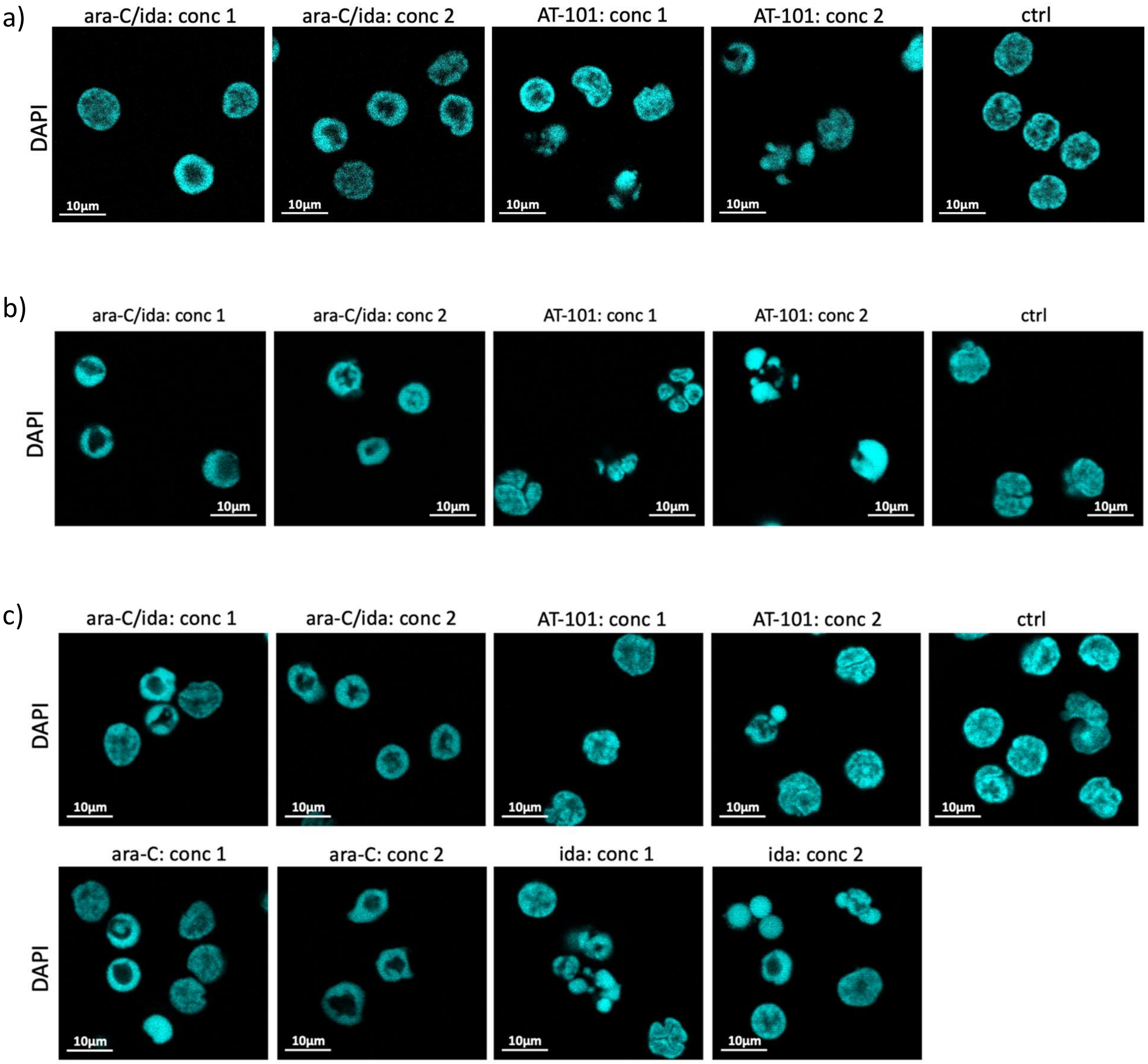

PBMCs from healthy donors upon ex vivo treatment with cytarabine and/or idarubicin, or AT-101. **a)** HB m 1951, **b)** UL f 1948 **c)** SM f 1954. Drug treatment: 24 h. **ara-C/ida conc 1**: 5 µM ara-C + 0.3 µM ida; **ara-C/ida conc 2**: 15 µM ara-C + 0.9 µM ida; **ara-C conc 1**: 5 µM ara-C; **ara-C conc 2:** 15 µM ara-C; **ida conc 1:** 0.3 µM ida; **ida conc 2:** 0.9 µM ida; **AT-101 conc 1**: 10 µM AT-101; **AT-101 conc 2**: 30 µM AT-101. Following fixation and DAPI staining, cells we subjected to Cytospin prior to imaging.

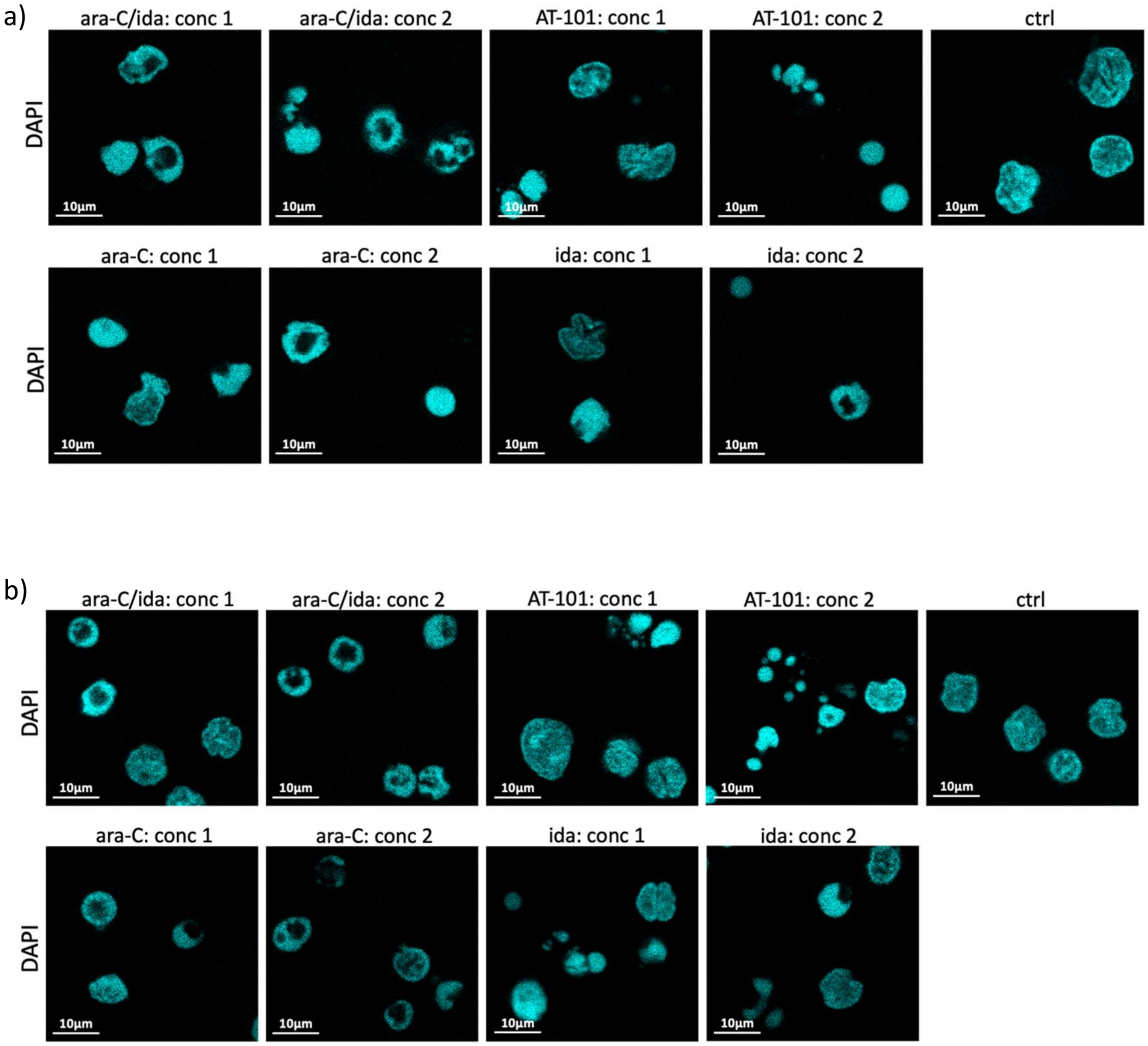

PBMCs from healthy donors upon ex vivo treatment with cytarabine and/or idarubicin, or AT-101. **a)** KA m 1958, **b)** MB m 1958. Drug treatment: 24 h. **ara-C/ida conc 1**: 5 µM ara-C + 0.3 µM ida; **ara-C/ida conc 2**: 15 µM ara-C + 0.9 µM ida; **ara-C conc 1**: 5 µM ara-C; **ara-C conc 2:** 15 µM ara-C; **ida conc 1:** 0.3 µM ida; **ida conc 2:** 0.9 µM ida; **AT-101 conc 1**: 10 µM AT-101; **AT-101 conc 2**: 30 µM AT-101. Following fixation and DAPI staining, cells we subjected to Cytospin prior to imaging.
