# Supplementary material for: PARP-1 improves leukemia outcomes by inducing parthanatos during chemotherapy
Source: Cell Rep Med. 2023 Sep 7;4(9):101191. doi: 10.1016/j.xcrm.2023.101191 (PMC10518631; doi:10.1016/j.xcrm.2023.101191)
Supplement: Data S3. Microscopy analyses of PBMCs from 8 patients with AML (M4/M5) taken immediately before and during 7+3 induction chemotherapy with ara-C and idarubicin, related to Figure 4 [file mmc7.pdf]

Supplementary Data Set 3: Microscopy analyses of PBMCs from 8 AML patients taken immediately before and during 7 + 3 induction chemotherapy with ara-C and idarubicin.

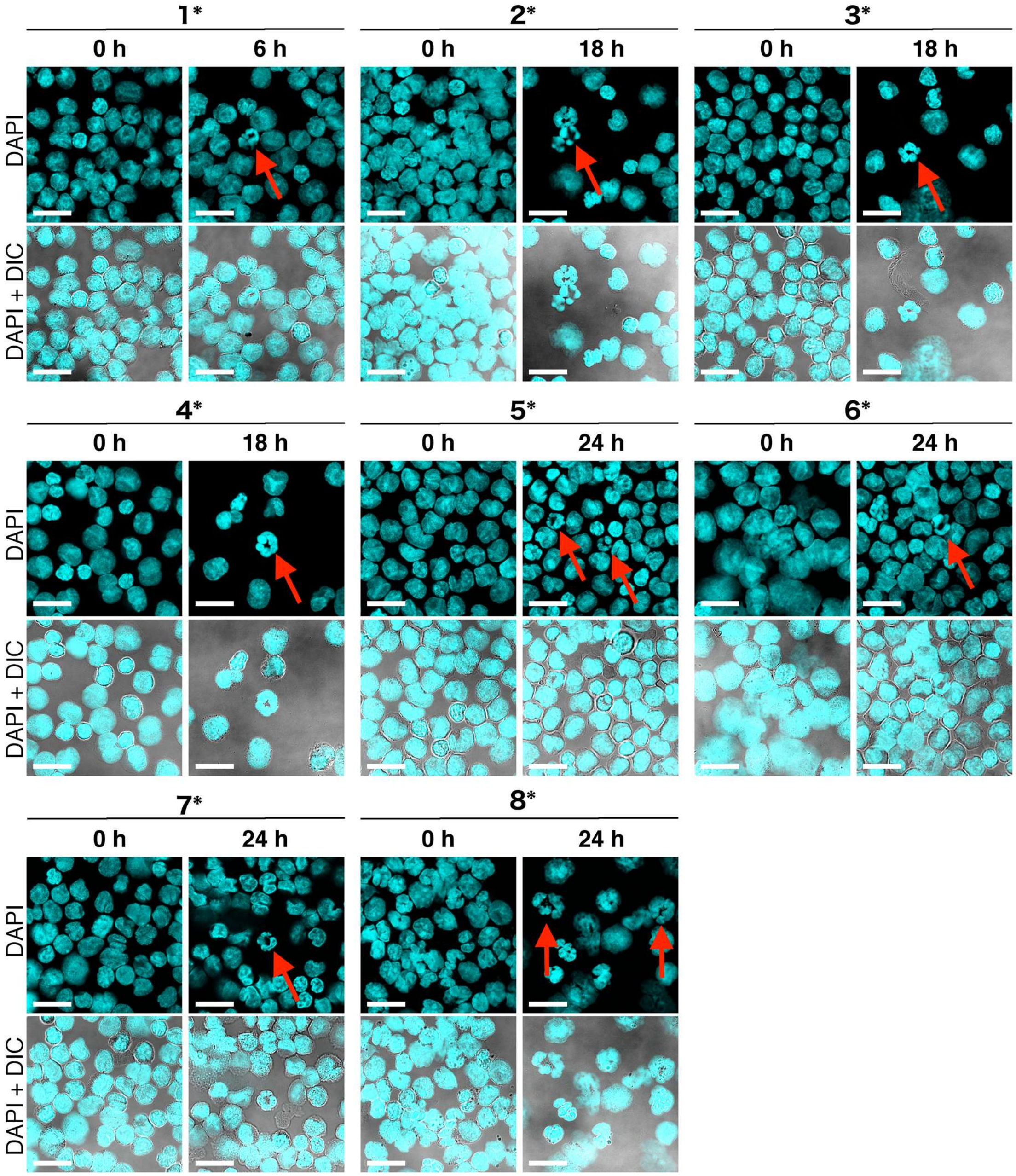

DAPI staining of monocytes from AML patients collected immediately before or 6 – 24 hours after the onset of "7 + 3" induction chemotherapy with cytarabine (200 mg/m^2/d) and idarubicin (12 mg/m^2/d). **1*** (patient ID# 17-008, 93% blasts, FAB M4eo, 6 h); **2*** (patient ID# 17-024, 52% blasts, FAB M4eo, 18 h); **3*** (patient ID# 17-038, 38% blasts, FAB M4, 24 h); **4*** (patient ID# 17-040, 15% blasts, FAB M4, 24 h); **5*** (patient ID# 17-064, 1% blasts, FAB M5, 24 h); **6*** (patient ID# 17-068, 26% blasts, FAB M4, 24 h); **7*** (patient ID# 17-110, 47% blasts, MDS-AML, 24 h); **8*** (patient ID# 17-115, FAB M5, 24 h). Scale bar: 15 µm.
