# Supplementary material for: PARP-1 improves leukemia outcomes by inducing parthanatos during chemotherapy
Source: Cell Rep Med. 2023 Sep 7;4(9):101191. doi: 10.1016/j.xcrm.2023.101191 (PMC10518631; doi:10.1016/j.xcrm.2023.101191)
Supplement: Data S4. Flow cytometry and microscopy analyses of 18 AML patient samples exhibiting two parthanatos features, related to Figures 4 and 5A [file mmc8.pdf]

Supplementary Data Set 4: Flow cytometry and microscopy analyses of 18 AML patient samples exhibiting two parthanatos features.

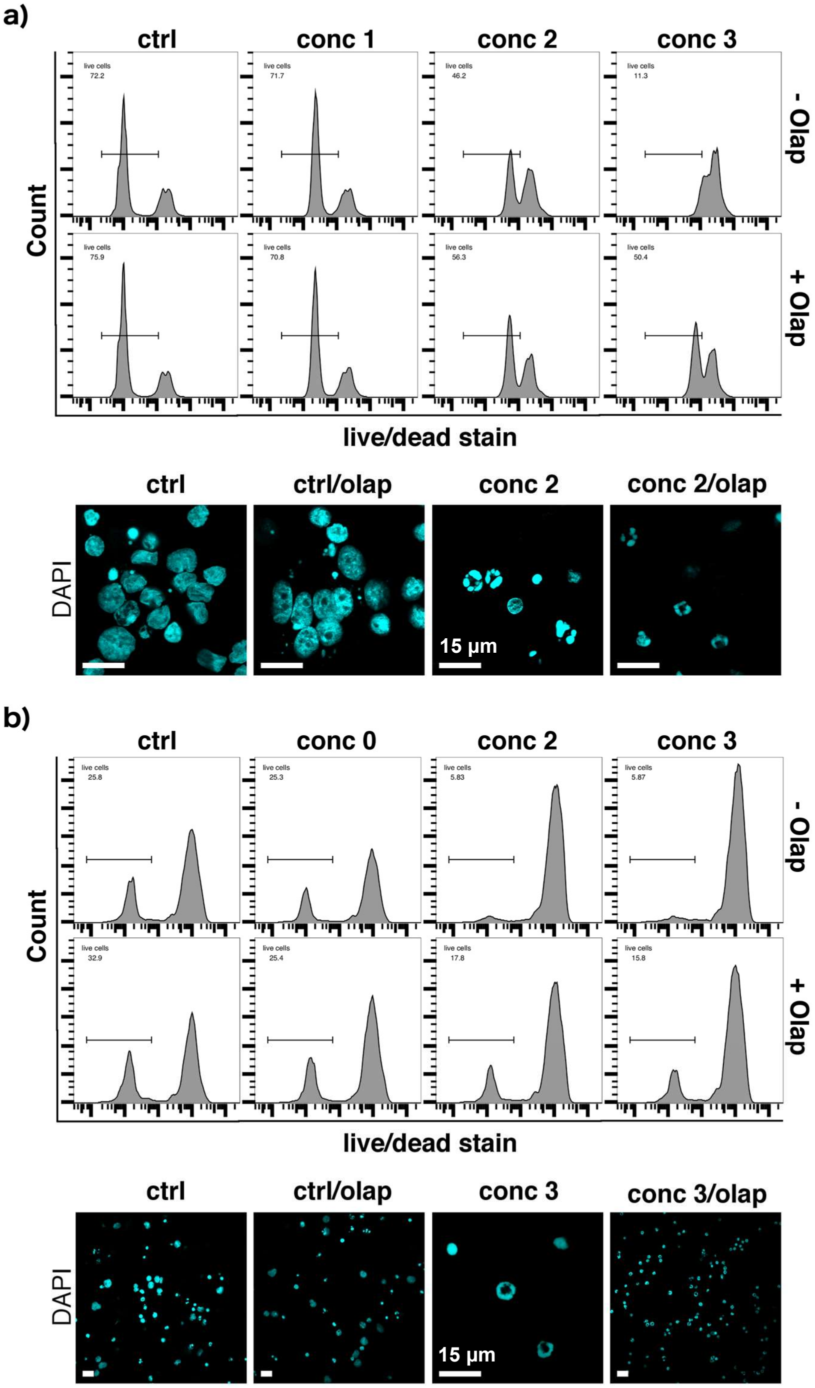

Parthanatos features in primary cells from AML donors according to toxicity rescue by Olaparib (Olap) and the presence of ring-shaped nuclei examined by DAPI staining. **a)** 1 / 04-032 and **b)** 2 / 04-045. Pretreatment: 1 µM Olaparib o/n; drug treatment: 24 h. Conc 0: 1 µM ara-C + 0.06 µM ida, conc 1: 5 µM ara-C + 0.3 µM ida, conc 2: 15 µM ara-C + 0.9 µM ida, conc 3: 30 µM ara-C + 1.8 µM ida.

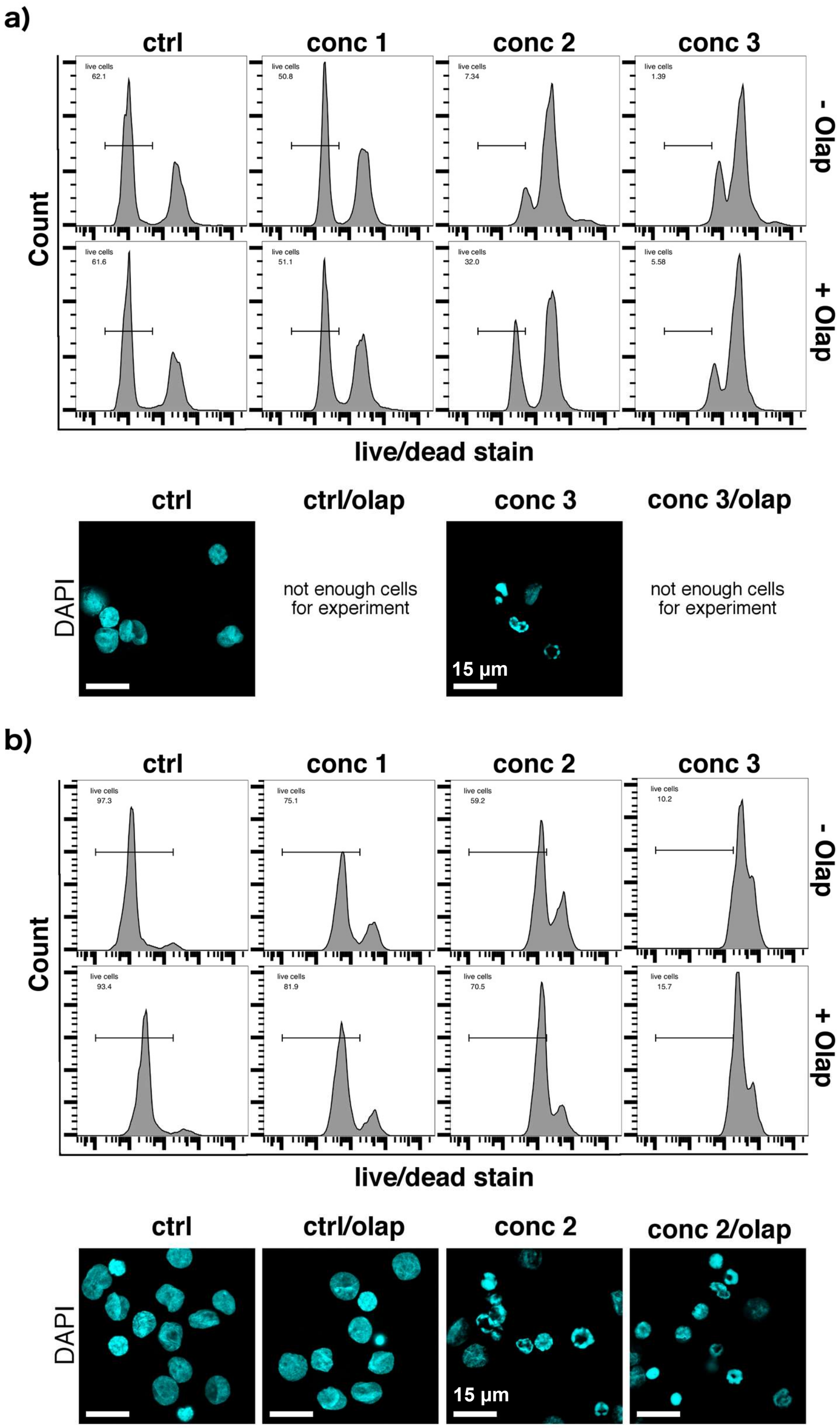

Parthanatos features in primary cells from AML donors according to toxicity rescue by Olaparib (Olap) and the presence of ring-shaped nuclei examined by DAPI staining. **a)** 3 / 05-002 and **b)** 4 / 15-084. Pretreatment: 1 µM Olaparib o/n; drug treatment: 24 h. Conc 1: 5 µM ara-C + 0.3 µM ida, conc 2: 15 µM ara-C + 0.9 µM ida, conc 3: 30 µM ara-C + 1.8 µM ida.

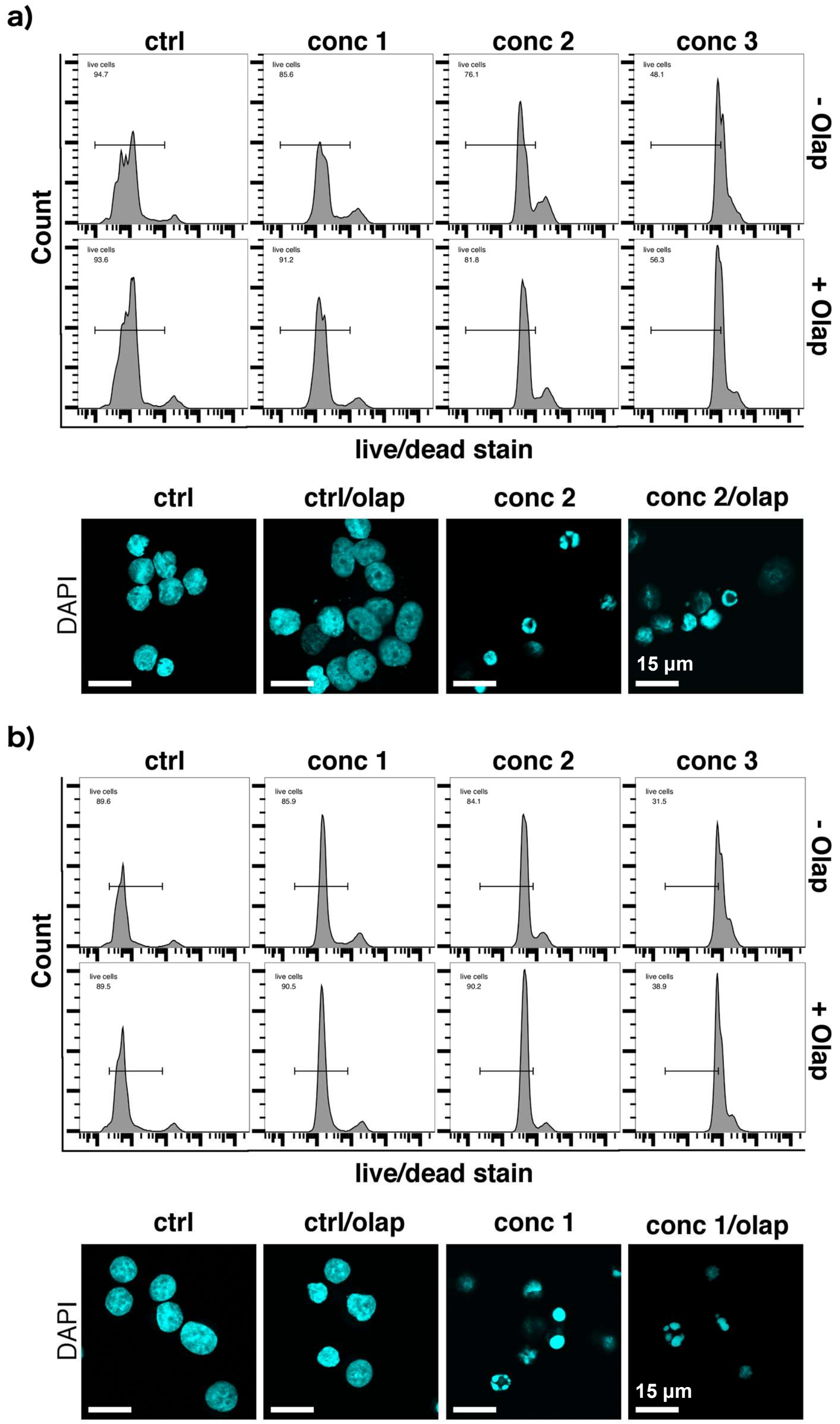

Parthanatos features in primary cells from AML donors according to toxicity rescue by Olaparib (Olap) and the presence of ring-shaped nuclei examined by DAPI staining. **a)** 5 /15-105 and **b)** 6 / 16-092. Pretreatment: 1 µM Olaparib o/n; drug treatment: 24 h. Conc 1: 5 µM ara-C + 0.3 µM ida, conc 2: 15 µM ara-C + 0.9 µM ida, conc 3: 30 µM ara-C + 1.8 µM ida.

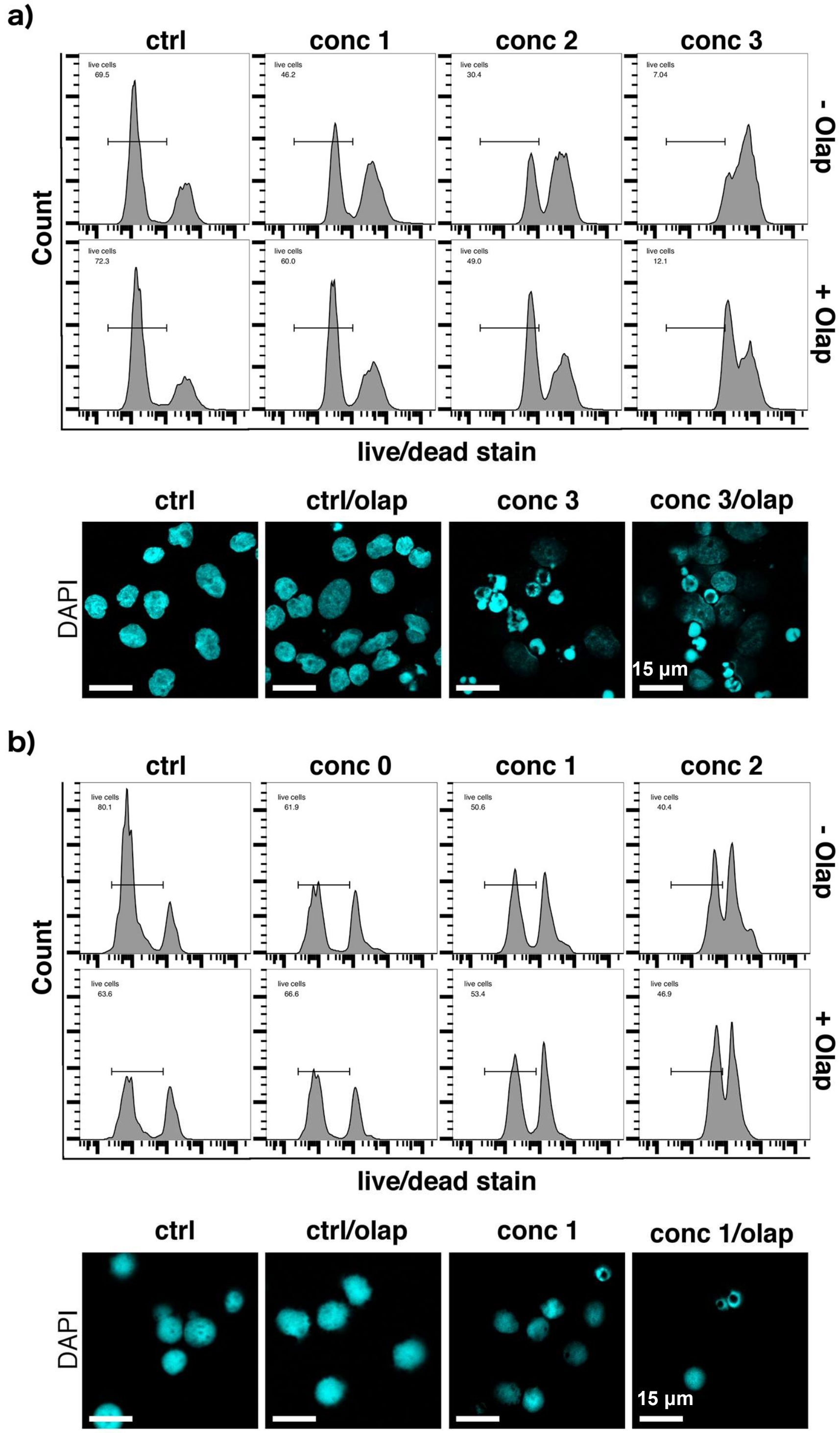

Parthanatos features in primary cells from AML donors according to toxicity rescue by Olaparib (Olap) and the presence of ring-shaped nuclei examined by DAPI staining. **a)** 7 / 17-008 and **b)** 8 / 17-016. Pretreatment: 1 µM Olaparib o/n; drug treatment: 24 h. Conc 0: 1 µM ara-C + 0.06 µM ida, conc 1: 5 µM ara-C + 0.3 µM ida, conc 2: 15 µM ara-C + 0.9 µM ida, conc 3: 30 µM ara-C + 1.8 µM ida.

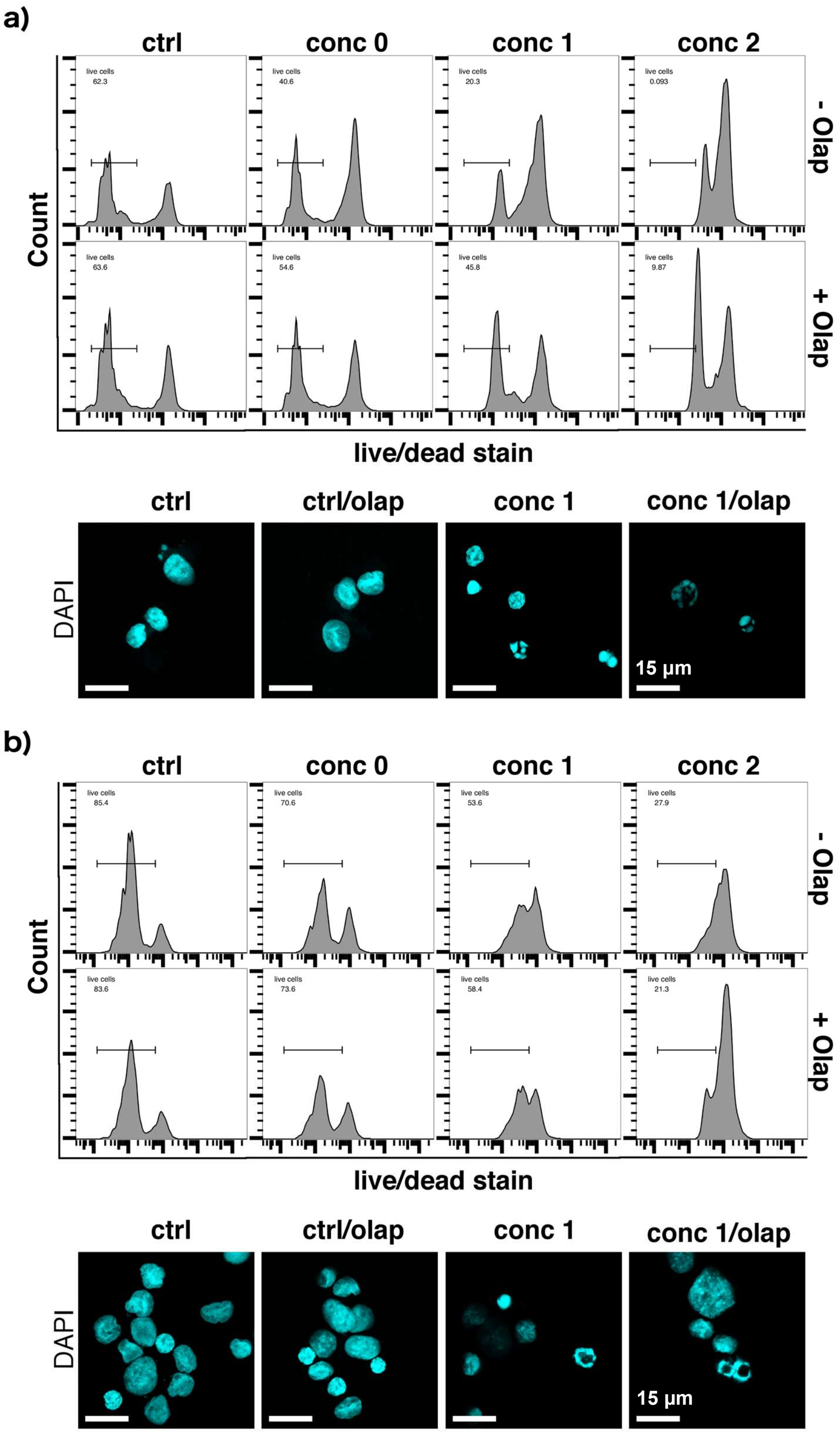

Parthanatos features in primary cells from AML donors according to toxicity rescue by Olaparib (Olap) and the presence of ring-shaped nuclei examined by DAPI staining. **a)** 9 / 17-036 and **b)** 10 / 17-063. Pretreatment: 1 µM Olaparib o/n; drug treatment: 24 h. Conc 0: 1 µM ara-C + 0.06 µM ida, conc 1: 5 µM ara-C + 0.3 µM ida, conc 2: 15 µM ara-C + 0.9 µM ida.

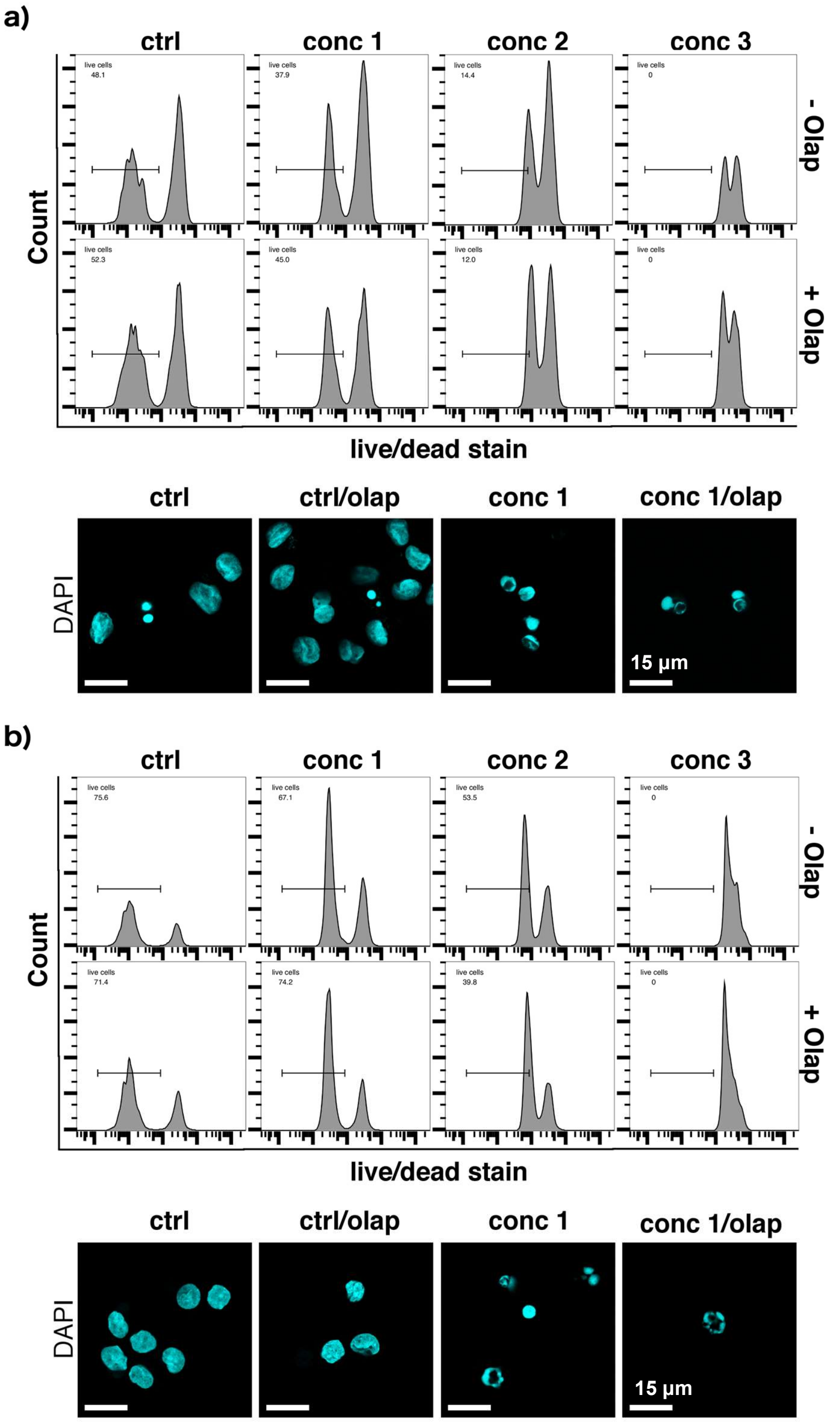

Parthanatos features in primary cells from AML donors according to toxicity rescue by Olaparib (Olap) and the presence of ring-shaped nuclei examined by DAPI staining. **a)** 11* / PID 145 and **b)** 12* / PID 160. Pretreatment: 1 µM Olaparib o/n; drug treatment: 24 h. Conc 1: 5 µM ara-C + 0.3 µM ida, conc 2: 15 µM ara-C + 0.9 µM ida, conc 3: 30 µM ara-C + 1.8 µM ida. *Bone marrow isolates.

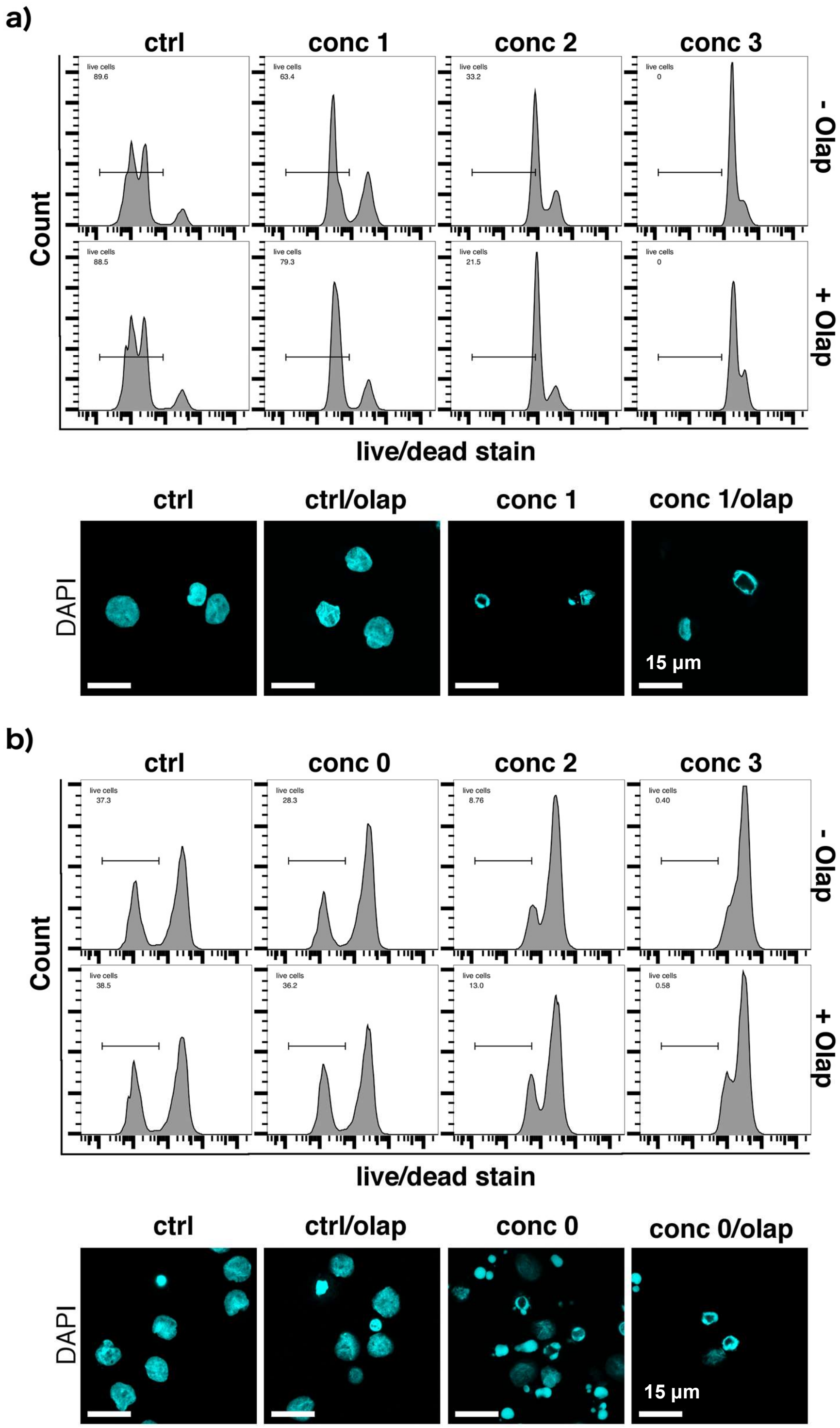

Parthanatos features in primary cells from AML donors according to toxicity rescue by Olaparib (Olap) and the presence of ring-shaped nuclei examined by DAPI staining. **a)** 13* / PID 198 and **b)** 14 / PID 752. Pretreatment: 1 µM Olaparib o/n; drug treatment: 24 h. Conc 0: 1 µM ara-C + 0.06 µM ida, conc 1: 5 µM ara-C + 0.3 µM ida, conc 2: 15 µM ara-C + 0.9 µM ida, conc 3: 30 µM ara-C + 1.8 µM ida. *Bone marrow isolates.

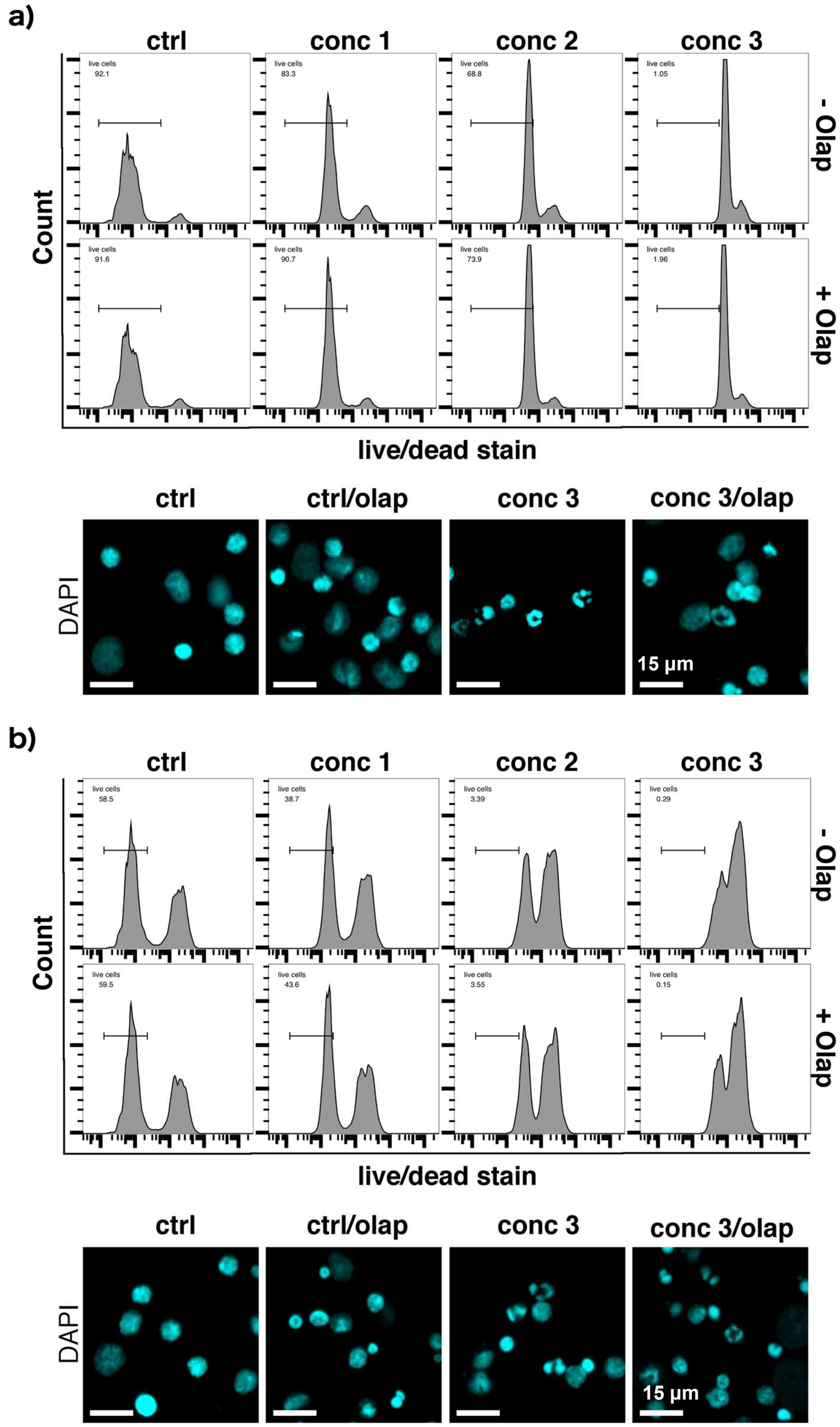

Parthanatos features in primary cells from AML donors according to toxicity rescue by Olaparib (Olap) and the presence of ring-shaped nuclei examined by DAPI staining. **a)** 15 / PID 52 and **b)** 16 / PID 528. Pretreatment: 1 µM Olaparib o/n; drug treatment: 24 h. Conc 1: 5 µM ara-C + 0.3 µM ida, conc 2: 15 µM ara-C + 0.9 µM ida, conc 3: 30 µM ara-C +1.8 µM ida.

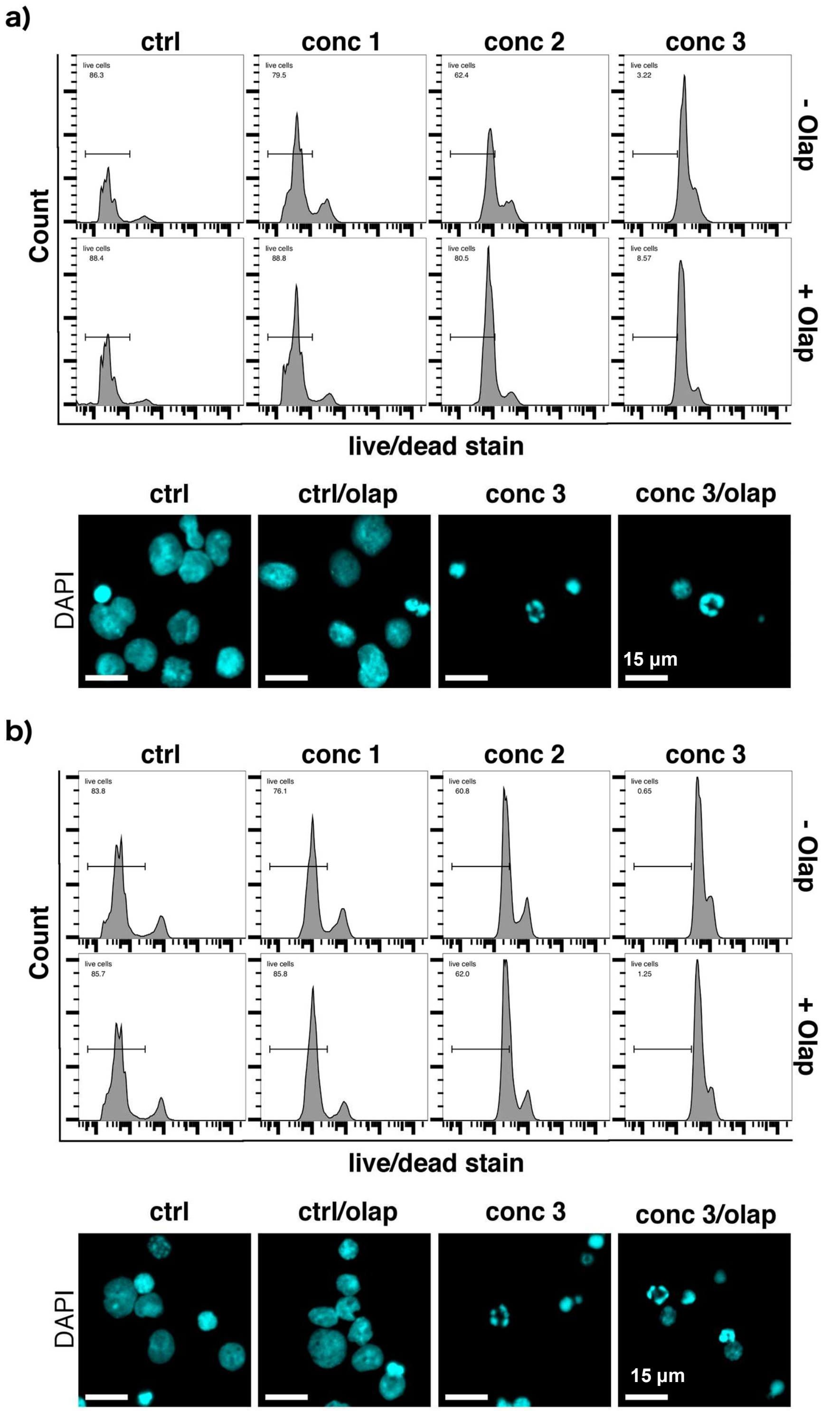

Parthanatos features in primary cells from AML donors according to toxicity rescue by Olaparib (Olap) and the presence of ring-shaped nuclei examined by DAPI staining. **a)** 17 / PID 242 and **b)** 18 / PID 127. Pretreatment: 1 µM Olaparib o/n; drug treatment: 24 h. Conc 1: 5 µM ara-C + 0.3 µM ida, conc 2: 15 µM ara-C + 0.9 µM ida, conc 3: 30 µM ara-C + 1.8 µM ida.
