# Supplementary material for: PARP-1 improves leukemia outcomes by inducing parthanatos during chemotherapy
Source: Cell Rep Med. 2023 Sep 7;4(9):101191. doi: 10.1016/j.xcrm.2023.101191 (PMC10518631; doi:10.1016/j.xcrm.2023.101191)
Supplement: Data S5. Flow cytometry and microscopy analyses of 21 AML patient samples exhibiting zero or one parthanatos feature, related to Figures 4 and 5A [file mmc9.pdf]

Supplementary Data Set 5: Flow cytometry and microscopy analyses of 21 AML patient samples exhibiting zero or one parthanatos features.

a)

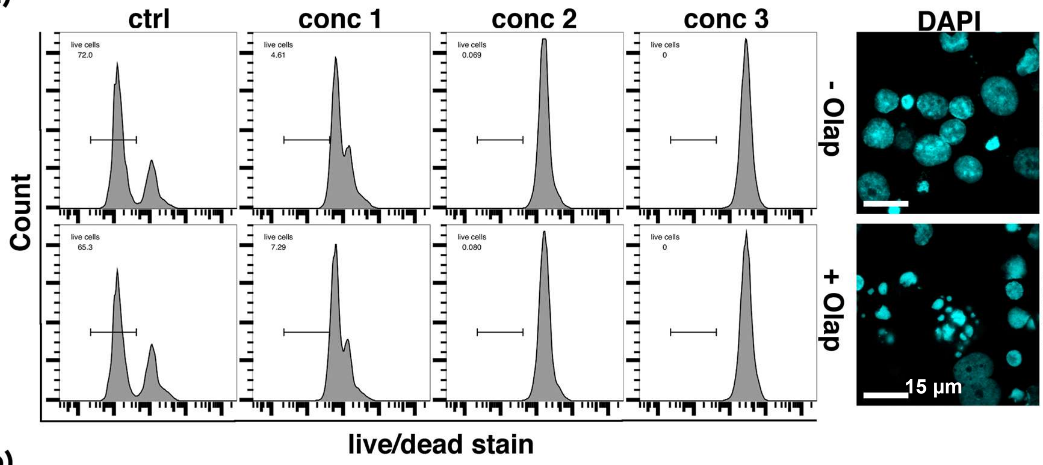

b)

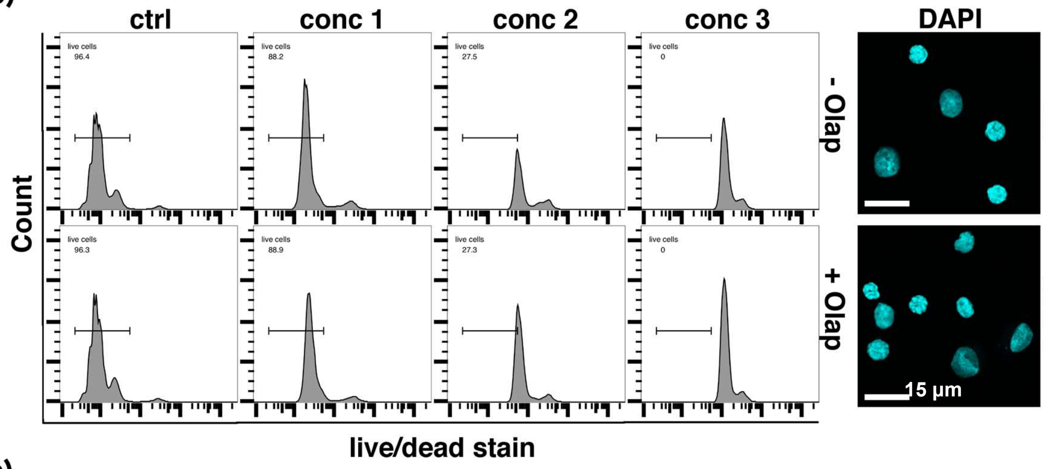

c)

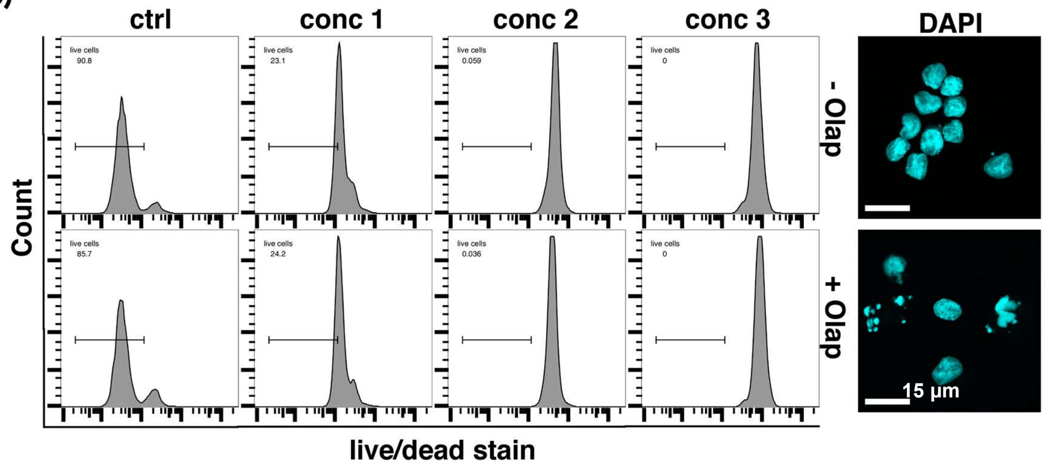

No apparent parthanatos features in primary cells from AML donors according to toxicity rescue by Olaparib (Olap) and the presence of ring-shaped nuclei examined by DAPI staining. **a)** 19 / 04-015, **b)** 20 / 15-119 and **c)** 21 / 15-130. Pretreatment: 1 µM Olaparib o/n; drug treatment: 24 h. Conc 0: 1 µM ara-C + 0.06 µM ida, conc 1: 5 µM ara-C + 0.3 µM ida, conc 2: 15 µM ara-C + 0.9 µM ida, conc 3: 30 µM ara-C + 1.8 µM ida.

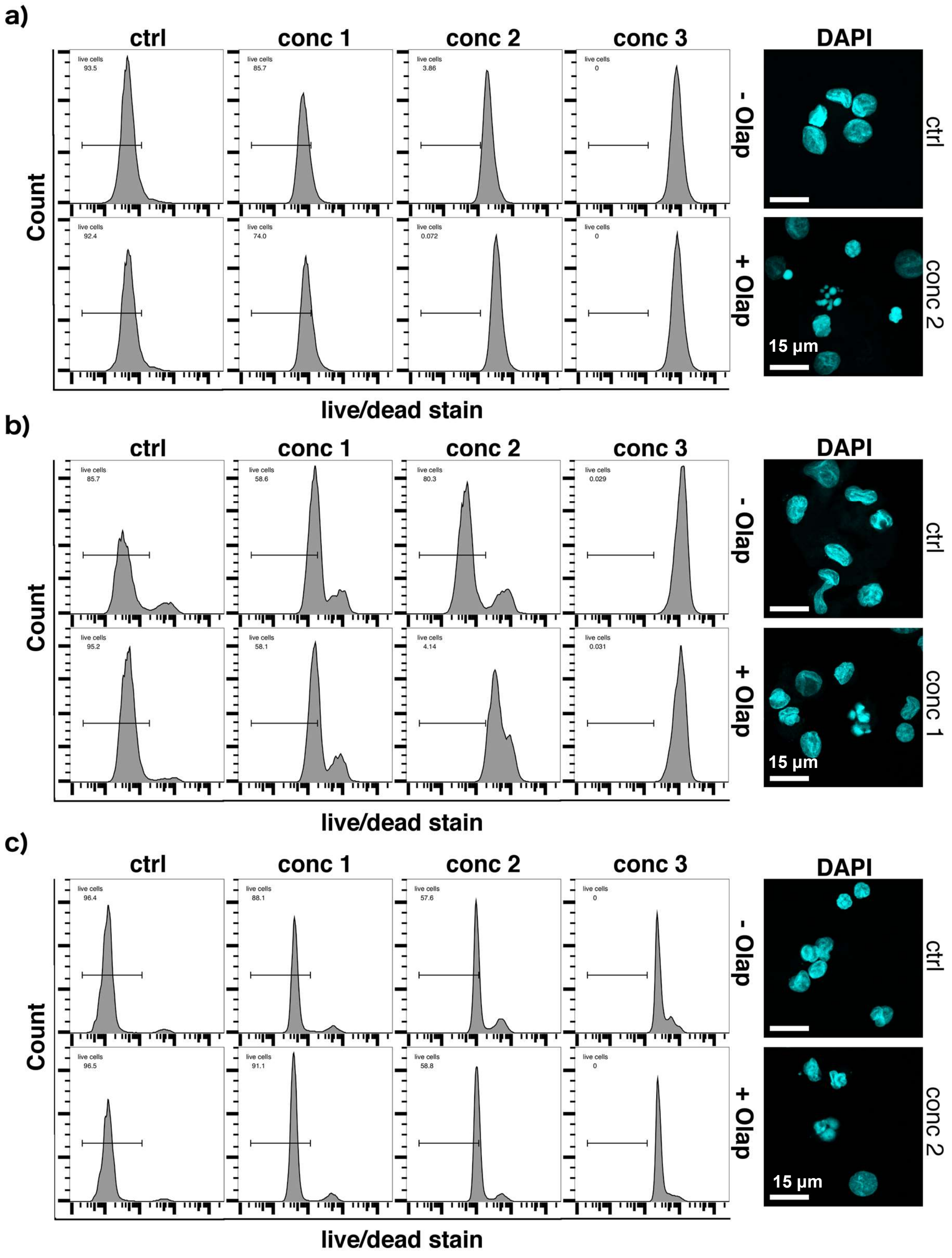

No apparent parthanatos features in primary cells from AML donors according to toxicity rescue by Olaparib (Olap) and the presence of ring-shaped nuclei examined by DAPI staining. **a)** 22 / 16-007, **b)** 23 / 16-062 and **c)** 24 / 16-068. Pretreatment: 1 µM Olaparib o/n; drug treatment: 24 h. Conc 1: 5 µM ara-C + 0.3 µM ida, conc 2: 15 µM ara-C + 0.9 µM ida, conc 3: 30 µM ara-C + 1.8 µM ida.

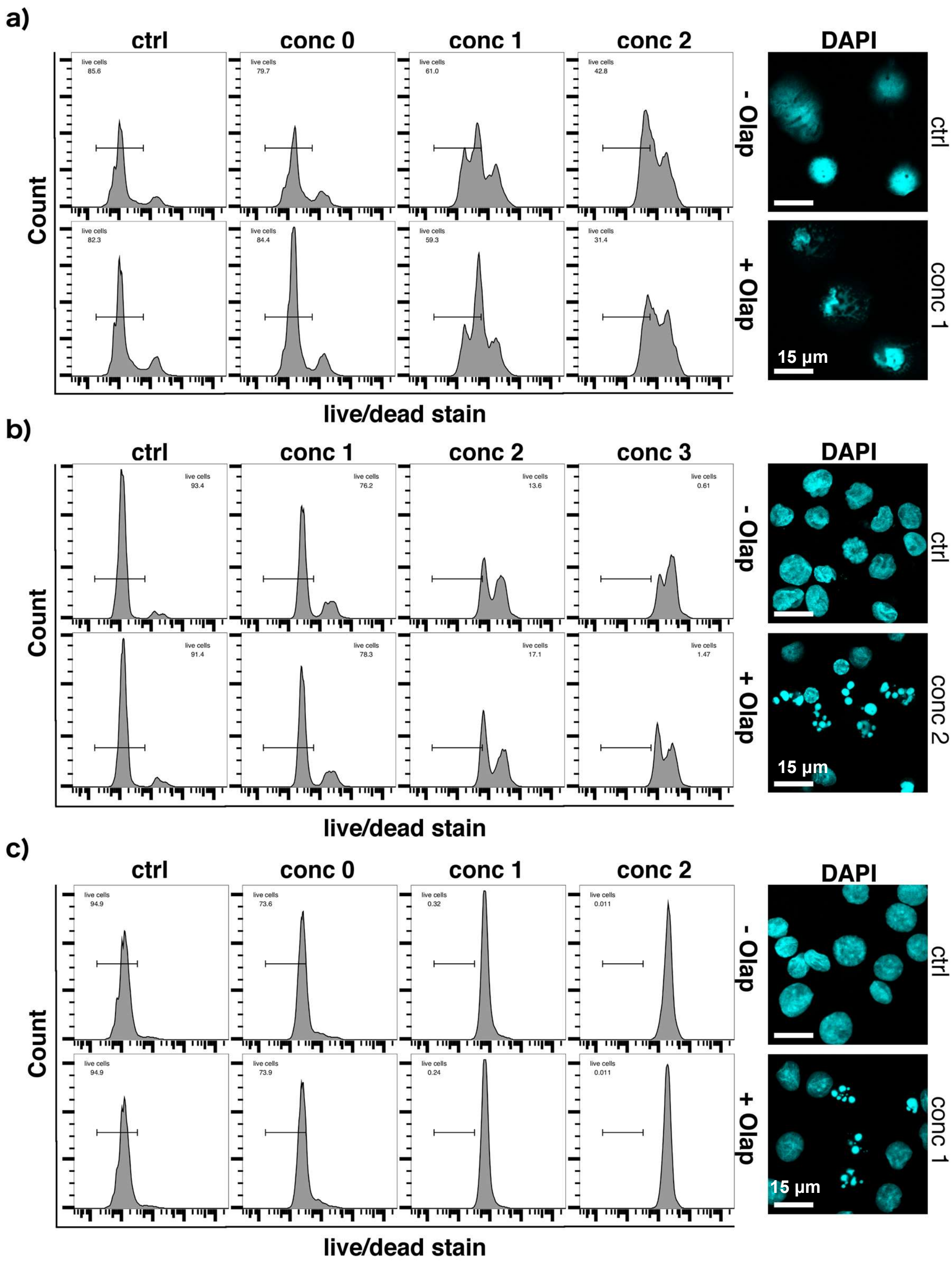

No apparent parthanatos features in primary cells from AML donors according to toxicity rescue by Olaparib (Olap) and the presence of ring-shaped nuclei examined by DAPI staining. **a)** 25 / 17-003, **b)** 26 / 17-024 and **c)** 27* / 17-039. Pretreatment: 1 µM Olaparib o/n; drug treatment: 24 h. Conc 0: 1 µM ara-C + 0.06 µM ida, conc 1: 5 µM ara-C + 0.3 µM ida, conc 2: 15 µM ara-C + 0.9 µM ida, conc 3: 30 µM ara-C + 1.8 µM ida. *Bone marrow isolate.

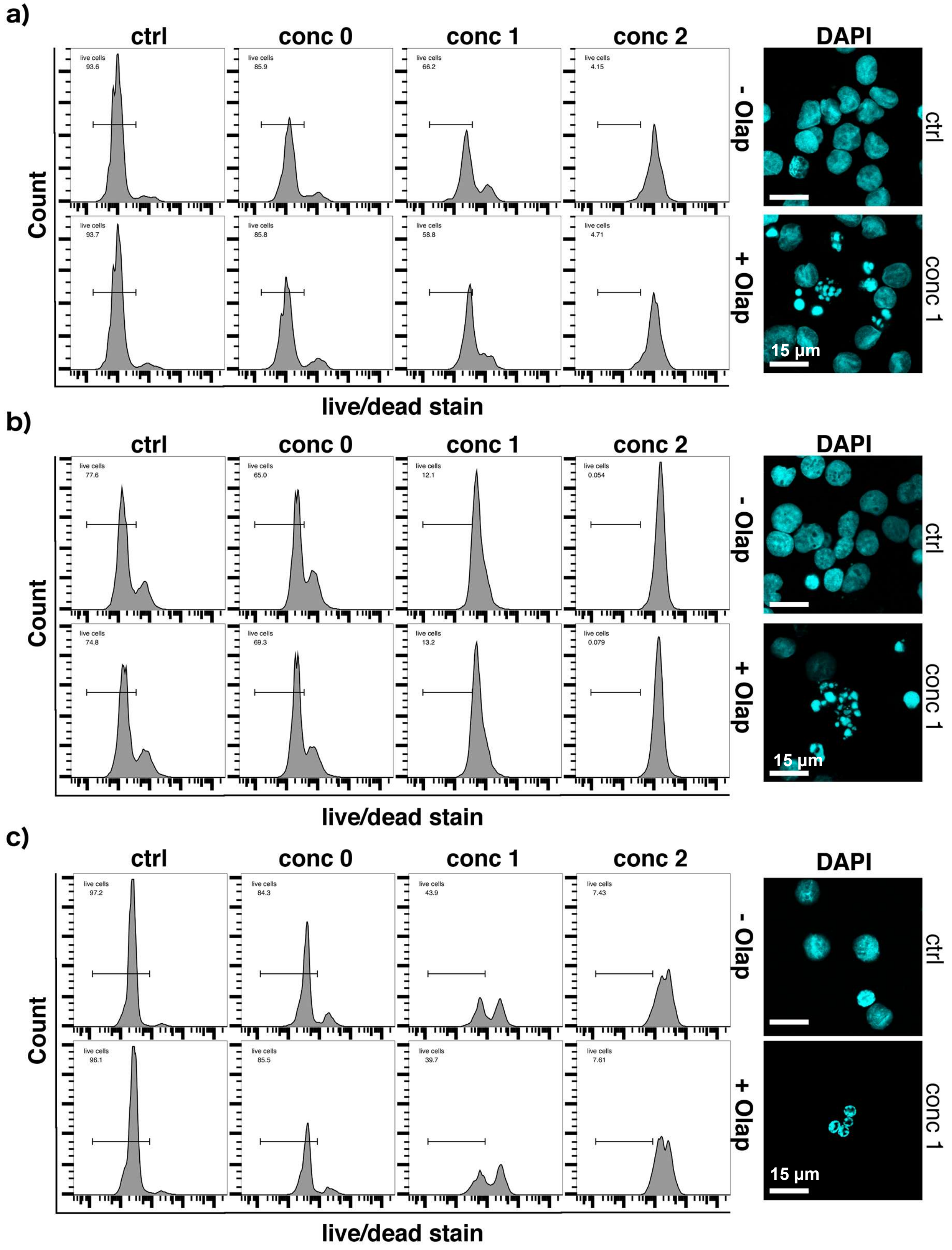

Zero (**a**,**b**) or one (**c**) apparent parthanatos feature in primary cells from AML donors according to toxicity rescue by Olaparib (Olap) and the presence of ring-shaped nuclei examined by DAPI staining. **a)** 28 / 17-045, **b)** 29* / 17-014 and **c)** 30* / PID 624. Pretreatment: 1 µM Olaparib o/n; drug treatment: 24 h. Conc 0: 1 µM ara-C + 0.06 µM ida, conc 1: 5 µM ara-C + 0.3 µM ida, conc 2: 15 µM ara-C + 0.9 µM ida. *Bone marrow isolates.

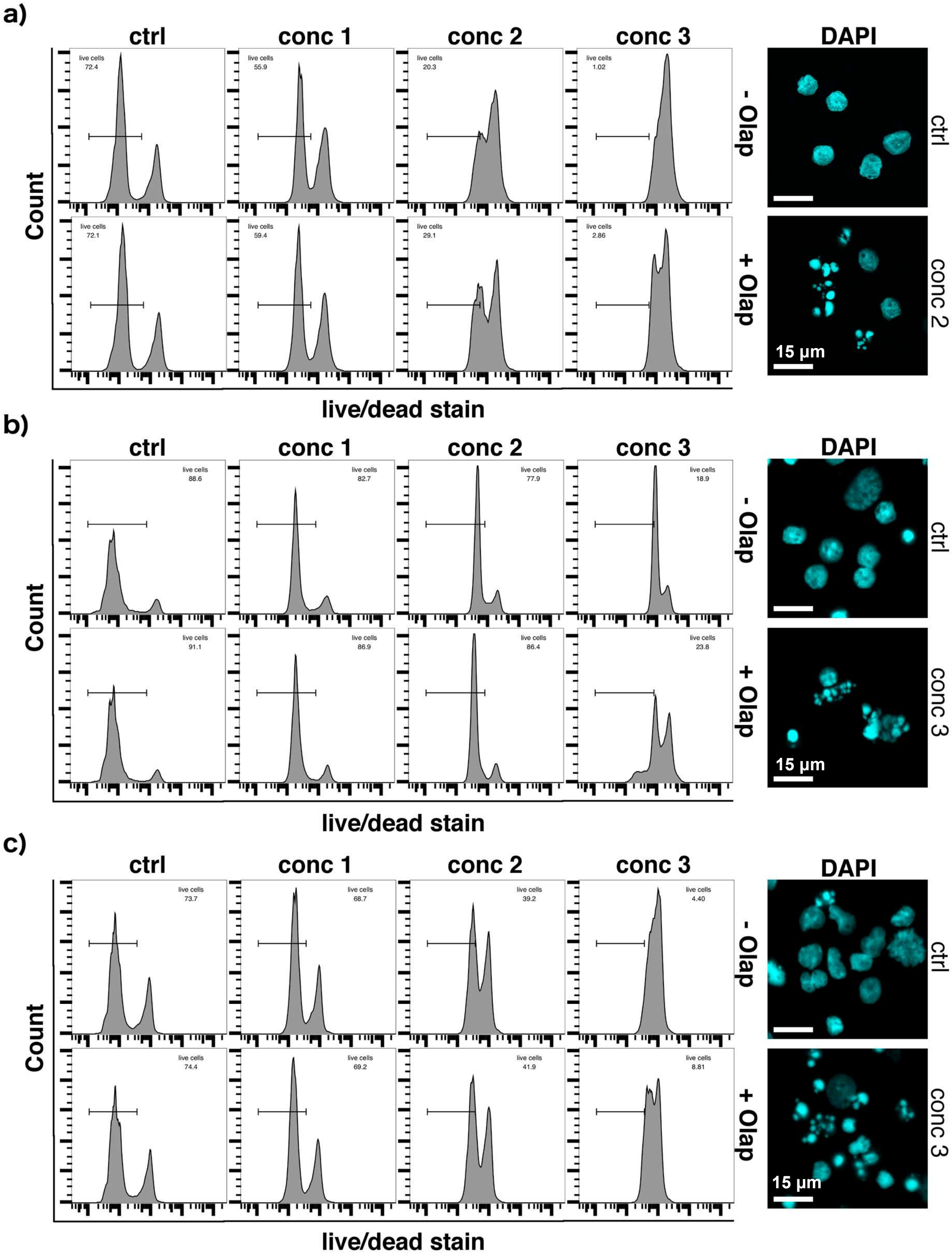

One (**a,b**) or zero (**c**) apparent parthanatos features in primary cells from AML donors according to toxicity rescue by Olaparib (Olap) and the presence of ring-shaped nuclei examined by DAPI staining. **a)** 31 / PID 625, **b)** 32 / PID 766 and **c)** 33 / PID 154. Pretreatment: 1 µM Olaparib o/n; drug treatment: 24 h. Conc 1: 5 µM ara-C + 0.3 µM ida, conc 2: 15 µM ara-C + 0.9 µM ida, conc 3: 30 µM ara-C + 1.8 µM ida.

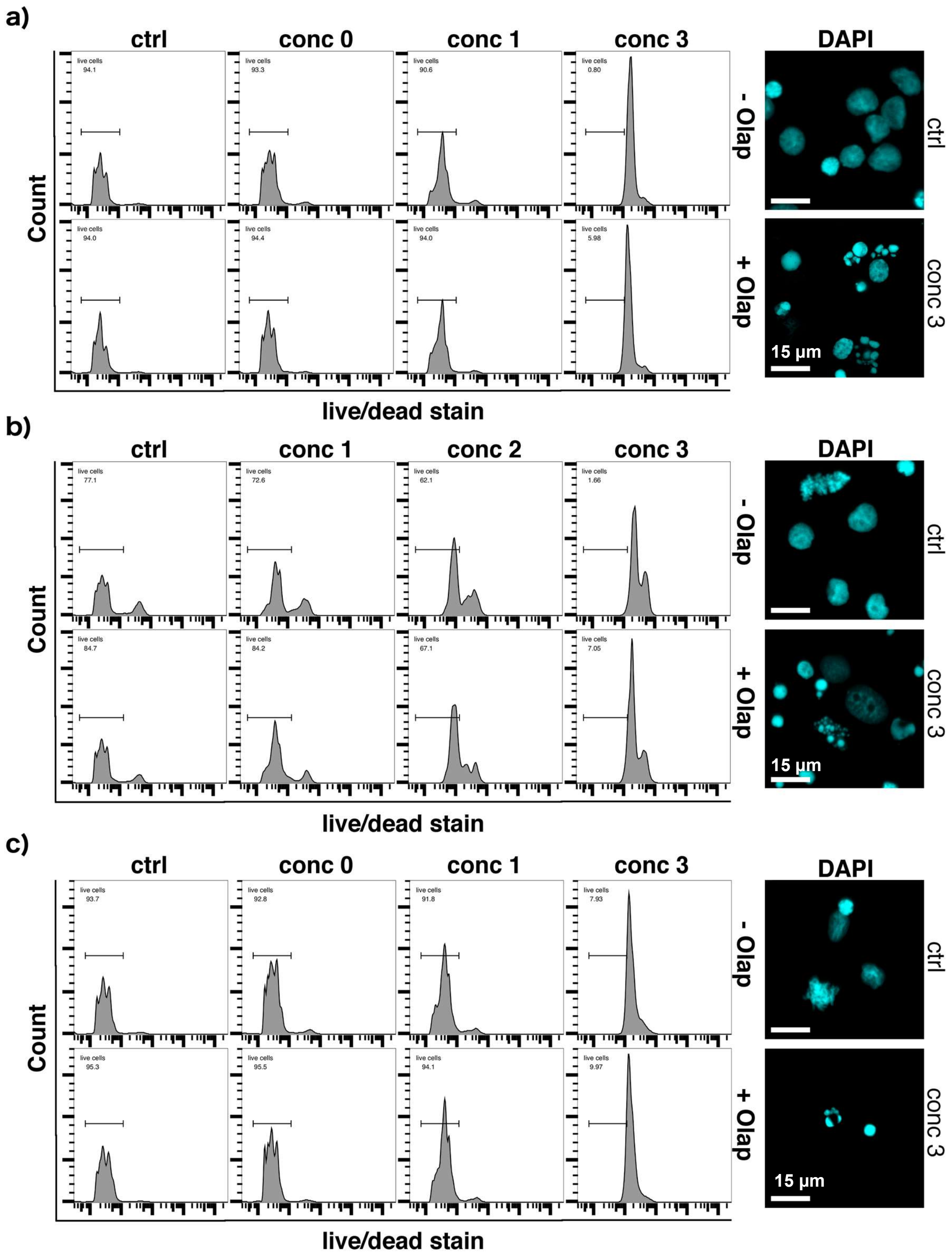

Zero (**a**) or one (**b**,**c**) apparent parthanatos feature in primary cells from AML donors according to toxicity rescue by Olaparib (Olap) and the presence of ring-shaped nuclei examined by DAPI staining. **a)** 34 / PID 218, **b)** 35 / PID 469 and **c)** 36 / PID 103. Pretreatment: 1 µM Olaparib o/n; drug treatment: 24 h. Conc 0: 1 µM ara-C + 0.06 µM ida, conc 1: 5 µM ara-C + 0.3 µM ida, conc 3: 30 µM ara-C + 1.8 µM ida.

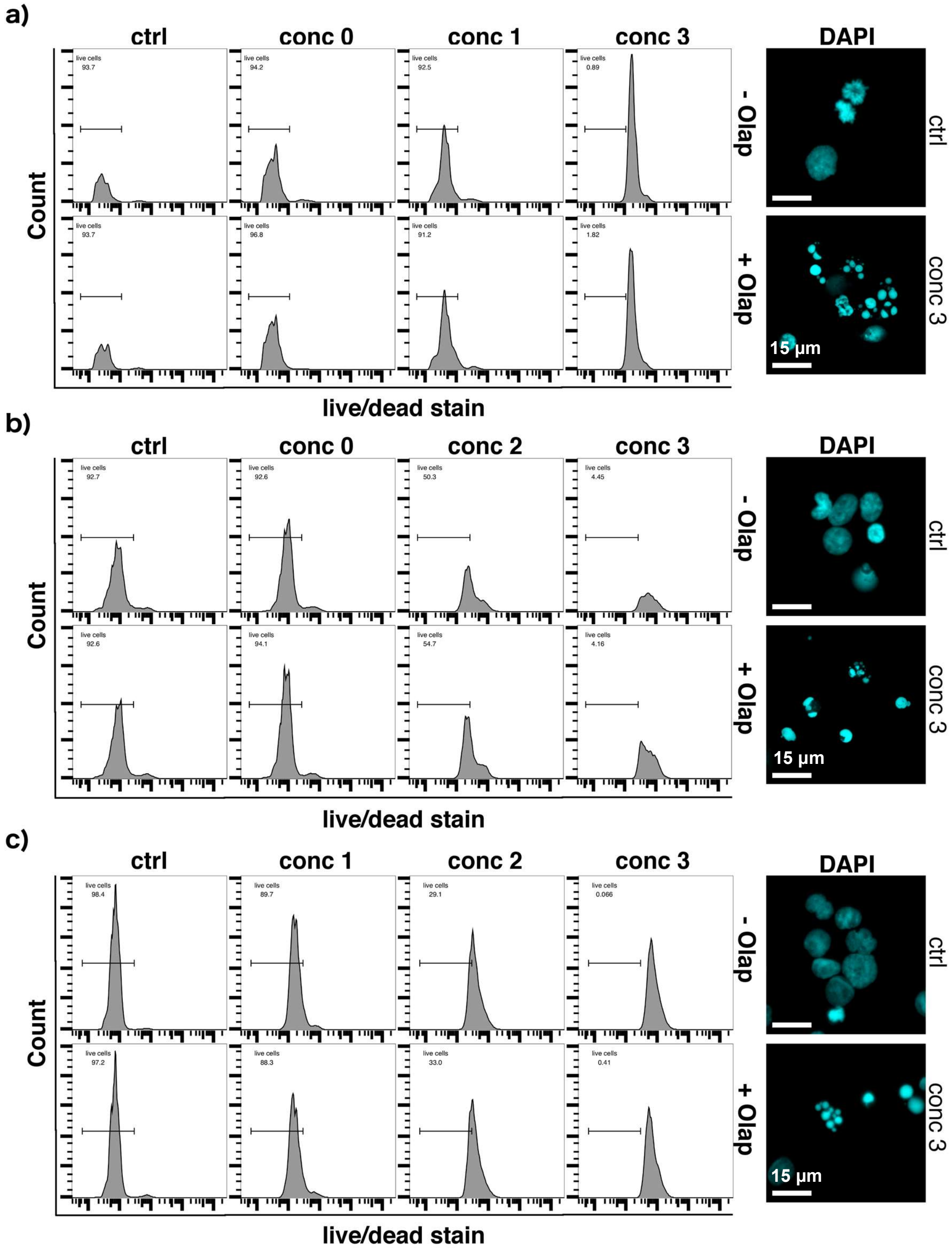

No apparent parthanatos features in primary cells from AML donors according to toxicity rescue by Olaparib (Olap) and the presence of ring-shaped nuclei examined by DAPI staining. **a)** 37/ PID 176, **b)** 38 / PID 171 and **c)** 39 / PID 517. Pretreatment: 1 µM Olaparib o/n; drug treatment: 24 h. Conc 0: 1 µM ara-C + 0.06 µM ida, conc 1: 5 µM ara-C + 0.3 µM ida, conc 2: 15 µM ara-C + 0.9 µM ida, conc 3: 30 µM ara-C + 1.8 µM ida.
